# Mitofusin-mediated contacts between mitochondria and peroxisomes regulate mitochondrial fusion

**DOI:** 10.1371/journal.pbio.3002602

**Published:** 2024-04-26

**Authors:** Cynthia Alsayyah, Manish K. Singh, Maria Angeles Morcillo-Parra, Laetitia Cavellini, Nadav Shai, Christine Schmitt, Maya Schuldiner, Einat Zalckvar, Adeline Mallet, Naïma Belgareh-Touzé, Christophe Zimmer, Mickaël M. Cohen

**Affiliations:** 1 Laboratoire de Biologie Moléculaire et Cellulaire des Eucaryotes, Sorbonne Université, CNRS, UMR8226, Institut de Biologie Physico-Chimique, Paris, France; 2 Institut Pasteur, Université Paris Cité, Imaging and Modeling Unit, F-75015 Paris, France; 3 Department of Molecular Genetics, Weizmann Institute of Science, Rehovot, Israel; 4 Ultrastructural BioImaging Core Facility, C2RT, Institut Pasteur, Université Paris Cité, Paris, France; 5 Rudolf Virchow Center for Integrative and Translational Bioimaging, University of Würzburg, Würzburg, Germany; University of Geneva Faculty of Science: Universite de Geneve Faculte des Sciences, SWITZERLAND

## Abstract

Mitofusins are large GTPases that trigger fusion of mitochondrial outer membranes. Similarly to the human mitofusin Mfn2, which also tethers mitochondria to the endoplasmic reticulum (ER), the yeast mitofusin Fzo1 stimulates contacts between Peroxisomes and Mitochondria when overexpressed. Yet, the physiological significance and function of these “PerMit” contacts remain unknown. Here, we demonstrate that Fzo1 naturally localizes to peroxisomes and promotes PerMit contacts in physiological conditions. These contacts are regulated through co-modulation of Fzo1 levels by the ubiquitin–proteasome system (UPS) and by the desaturation status of fatty acids (FAs). Contacts decrease under low FA desaturation but reach a maximum during high FA desaturation. High-throughput genetic screening combined with high-resolution cellular imaging reveal that Fzo1-mediated PerMit contacts favor the transit of peroxisomal citrate into mitochondria. In turn, citrate enters the TCA cycle to stimulate the mitochondrial membrane potential and maintain efficient mitochondrial fusion upon high FA desaturation. These findings thus unravel a mechanism by which inter-organelle contacts safeguard mitochondrial fusion.

## Abstract 2

The yeast mitofusin Fzo1 naturally localizes to peroxisomes to promote their tethering with mitochondria, but the physiological relevance of this contact is unclear. Here, the authors show that these Fzo1-mediated PerMit contacts are required to maintain mitochondrial fusion as fatty acid desaturation increases.

## Introduction

In vivo, mitochondria assemble as a tubular reticulum in physical contact with endomembrane systems such as the endoplasmic reticulum (ER), the vacuolar/lysosomal compartment, the plasma membrane, and the peroxisomes [1]. The morphology of the mitochondrial reticulum is maintained by an equilibrium between fission and fusion of its tubules. Mitochondrial fission as well as fusion of mitochondrial outer and inner membranes is all mediated by large GTPases of the dynamin-related proteins (DRPs) super-family [2]. Mitofusins, a subclass of DRPs integral to mitochondrial outer membranes, auto-oligomerize in *cis* (on the same membrane) and in *trans* (from opposite membranes) in a GTPase domain-dependent manner to trigger the tethering and the homotypic fusion of outer membranes [3]. This process is mediated by 2 distinct mitofusins (Mfn1 and Mfn2) in mammalian cells and a single one (Fzo1) in the yeast *Saccharomyces cerevisiae* (from hereon simply yeast) [4–7]. Notably, Mfn2 also localizes to ER membranes from where it can interact with mitochondrial Mfn1 and Mfn2, thereby promoting tethering between the ER and mitochondria [8]. Mfn2 was also found to mediate mitochondrial tethering with other organelles such as melanosomes [9]. Most intriguingly, overexpression of the yeast and mammalian mitofusins were more recently proposed to promote contacts between peroxisomes and mitochondria [10,11], calling for further investigation.

An obvious link between peroxisomes and mitochondria in yeast is beta-oxidation where fatty acids (FAs) enter peroxisomes to be catabolized into acetyl-CoA [12]. Depending on the availability of carbon sources, acetyl-CoA can then either transit to mitochondria to feed the tricarboxylic acid (TCA) cycle or be rerouted between the cytosol and peroxisomes to feed the glyoxylate cycle [13]. However, whether increased contacts between peroxisomes and mitochondria triggered by Fzo1 overexpression could employ these pathways is unknown [10].

Interestingly, Fzo1-dependent fusion of outer membranes is co-regulated by the ubiquitin–proteasome system (UPS) and by FA desaturation [14,15]. In yeast, FA desaturation is triggered by the Δ9-fatty acid desaturase Ole1. When the overall desaturation within cellular membranes decreases, the Rsp5 ubiquitin ligase activates the transcription factors of the *OLE1* gene [16–19]. Likewise, excess desaturation within acyl chains of FAs and phospholipids blocks the capacity of Rsp5 to activate the synthesis of Ole1 [18–21]. Regarding UPS-dependent regulation of mitochondrial fusion, the ubiquitin ligase Mdm30 promotes ubiquitination of Fzo1 during mitochondrial tethering and its subsequent degradation by the proteasome [22–24]. Conversely, the ubiquitin protease Ubp2 antagonizes the Mdm30-mediated ubiquitination of Fzo1 and slows down its degradation [14,25].

We have previously shown that Mdm30 also controls the stability of Ubp2 which is the main antagonist of Rsp5 [26,27], thereby connecting the degradation of Fzo1 with FA desaturation [14]. Upon down-regulation of the OLE1 pathway and low FA desaturation, Mdm30 promotes degradation of Ubp2 leading to un-antagonized and increased Mdm30-mediated turnover of Fzo1 [14]. Similarly, when FA desaturation is high, Ubp2 is stabilized and limits the extension of ubiquitin chains that Mdm30 conjugates to the mitofusin, resulting in stabilization of Fzo1 [14]. Mitochondrial fusion remains efficient when Fzo1 and FA desaturation are both low or when they are both high [14].

The balance between Fzo1 degradation and FA desaturation is essential for efficient mitochondrial fusion. How FA desaturation impacts mitochondrial fusion remains nonetheless obscure. In this regard, the evidence that overexpression of Fzo1 or its stabilization in the absence of Mdm30 promotes physical contacts between mitochondria and peroxisomes [10] offers interesting perspectives. Yet, the detection of Fzo1 on peroxisomes requires to be confirmed. Similarly, the physiological significance, the regulation and the function of Fzo1-mediated PerMit contacts remains unexplored. Transfer of material from peroxisomes to mitochondria can be expected, but the nature of this material, the conditions under which its transfer occurs, and the purpose of this transfer remain to be discovered.

Here, we demonstrate that Fzo1 naturally localizes to peroxisomes to associate with mitochondrial Fzo1. We find that this peroxisomal localization of Fzo1 is regulated by Mdm30 and FA desaturation. Our data indicate that Fzo1 accumulates on peroxisomes when FA desaturation increases to facilitate the transfer of peroxisomal citrate to mitochondria. In turn, citrate feeds the TCA cycle thereby maintaining the mitochondrial membrane potential and stimulating efficient mitochondrial fusion.

## Results

### Fzo1 naturally localizes to peroxisomes

Strong overexpression of Fzo1 in *WT* cells or its natural stabilization in *mdm30Δ* cells favor contacts between mitochondria and peroxisomes [10]. We aimed at confirming this possibility but also asked whether such peroxisomal function of Fzo1 could have physiological implications in WT cells.

**Fig 1:**
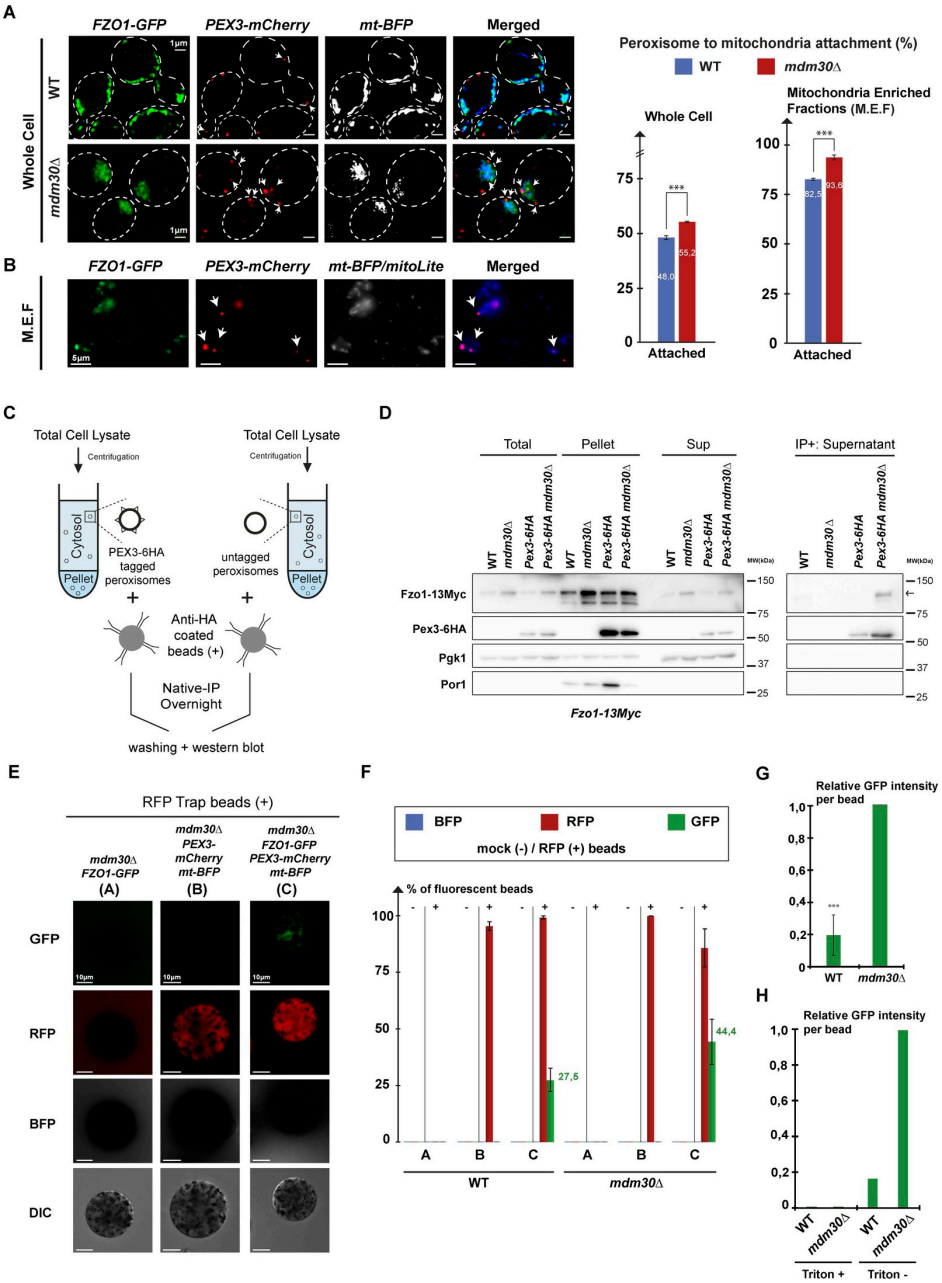
Fzo1 naturally localizes to peroxisomes. **(A and B)** Imaging of (A) whole cells analyzed by SIM or (B) their corresponding MEFs by conventional fluorescence microscopy. *WT* and *mdm30Δ* cells were genomically labeled with indicated fluorescent proteins (*MCY1675* and *MCY1677* cured from the *MDM30* shuffle plasmid). MEFs were additionally stained with mitolite blue to increase mitochondrial staining. Signals for GFP, mCherry, and BFP are shown in green, red, and gray (non-merged) or blue (merged), respectively. Scale bars for whole cells (delimited in white) or MEFs correspond to 1 or 5 μm, respectively. Graphs on the right depict the percentage of peroxisomal attachment (mCherry signals) to mitochondria (GFP/BFP signals) in *WT* (blue bars) and *mdm30Δ* (red bars) whole cells (left) or MEFs (right). White arrows indicate peroxisomes in contact with mitochondria. Error bars represent the SEM (standard error of the mean) from 3 independent experiments. ***P < 0.005 (one-way analysis of variance (ANOVA)). **(C and D)** Native Immuno-Precipitation of peroxisomes from *WT* and *mdm30Δ* cells genomically labeled for both *PEX3-6HA* and *FZO1-13MYC* (*MCY1488 and MCY1490*) or *FZO1-13MYC* only (*MCY1119 and MCY1131*). Left scheme (C): Cells were processed for fractionation assays to yield whole cell (Total), cytosol (Sup), and membrane (Pellet) fractions. Cytosol fractions were then split in 2 halves and incubated O.N. with anti-HA coated (IP+) beads in the absence of detergent to pull-down Pex3-6HA native peroxisomes specifically. (D) Western blots (WB): After washing, all fractions (Total, Pellet, Sup, and IP+) were processed for western blotting with anti-Myc, anti-HA, anti-PGK, and anti-Porin. Molecular weights in kDa are indicated on the right. The arrow on the right indicates Fzo1-13Myc. **(E and F)** Same experiment as in (C and D) but with *WT* and *mdm30Δ* cells genomically labeled for *PEX3-mCherry*, *FZO1-GFP*, and *mt-BFP* (*MCY1667*, *MCY1591*, *MCY1675* and *MCY1673*, *MCY1597*, *MCY1677* cured from the *MDM30* shuffle plasmid), mock (−) or RFP (+) Trap beads and analysis of beads with DIC or fluorescence microscopy of GFP (green), RFP (red), or BFP (gray); see also S2A Fig. Scale bars for RFP Trap beads in (E) correspond to 10 μm. The corresponding mock control is shown in S2B Fig. The readouts for *WT* conditions were identical to the *mdm30Δ* beads that are shown. The graph in (F) depicts the percentage of beads with RFP (red), GFP (green), or BFP (blue) fluorescence. **(G)** Average GFP fluorescence intensity per GFP positive beads from F in *WT* as compared to *mdm30Δ* conditions. **(H)** Same as (G) with GFP positive beads from S2C in the absence (Triton-) or in the presence of detergent (Triton+). Error bars represent the SEM from 3 independent experiments. Underlying data for quantifications can be found in S1 Data. Uncropped scans are depicted in S1 Raw Images. BFP, blue fluorescent protein; DIC, differential interference contrast; GFP, green fluorescent protein; MEF, mitochondrial-enriched fraction; RFP, red fluorescent protein; SIM, structured illumination microscopy.

As expected, peroxisomes labeled with the peroxisomal membrane protein Pex3-mCherry and mitochondria labeled with both fully functional Fzo1-GFP (green fluorescent protein; S1A Fig) and mito-BFP (blue fluorescent protein) showed a 7% increase in mutual contacts (as defined in Material and methods) in *mdm30Δ* as compared to *WT* whole cells (Fig 1A). In mitochondrial-enriched fractions, the total of peroxisomes in contact with mitochondria increased overall (Fig 1B). This is because the cytosolic supernatant containing free peroxisomes was discarded during preparation of the membrane fraction. Yet, this analysis of PerMit contacts in vitro confirmed the in vivo results seen in *WT* and *mdm30Δ* cells, thus ruling out the contribution of the cytoskeleton and the confinement of intracellular organelles but confirming bona fide contacts between mitochondria and peroxisomes in vivo.

Fzo1 accumulates in the cytosolic fraction of *mdm30Δ* cells that is devoid of mitochondria [10]. This extra-mitochondrial localization of Fzo1 correlates with the increased PerMit contacts seen in the absence of Mdm30 (Fig 1A and 1B) which suggests that Fzo1 could localize on peroxisomes. Consistent with this, immuno-electron microscopy (iEM) analysis on WT cells grown in Oleate-containing media (S1B Fig) allowed detecting Fzo1 on mitochondria (S1C Fig) but also on circular structures corresponding to peroxisomes (S1D and S1E Fig). Yet, iEM only detects peroxisomes that proliferate in Oleate-containing media (S1B Fig) where *mdm30Δ* cells do not grow (S4A Fig). We thus designed a specific protocol to circumvent this limitation. *WT* and *mdm30Δ* cells where *FZO1* and *PEX3* are respectively labeled with 13-Myc and 6-HA epitopes at their genomic loci, were grown in Dextrose-containing media and subjected to fractionation assays to separate the cytosolic supernatants from the membrane pellets (Fig 1C). As expected, the supernatants were positive for cytosolic PGK but negative for the mitochondrial Porin (Fig 1D; Total, Pellet, Sup fractions). The peroxisomal protein Pex3-6HA was detected in both the membrane and cytosol fractions (Fig 1D; compare Sup and Pellet fractions). Consistent with previous findings [10], we also detected a significant signal of Fzo1-13Myc in the supernatant of *mdm30Δ* cells and to a lesser extent but more surprisingly in the supernatant of WT cells (Fig 1D; Sup fraction). We reasoned that immunoprecipitation of cytosolic Pex3-6HA in the absence of detergent could pull-down native peroxisomes that are not bound to membranes and could be probed for the presence of Fzo1-13Myc (Fig 1C). This led to detection of a significant and specific Myc signal on presumably native peroxisomes from *mdm30Δ* cells (Fig 1D; IP+, anti-myc).

It was important confirming this observation with a distinct readout. We thus repeated the same protocol of native peroxisome immunoprecipitation but with cells where mitochondria were labeled with mito-BFP and in which either *PEX3* or *FZO1* or both were genomically labeled with mCherry and GFP, respectively. Following incubation with *WT* and *mdm30Δ* cells supernatants, Mock and red fluorescent protein (RFP) trap-coated beads were analyzed by fluorescence microscopy (S2A Fig). RFP traps have an established capacity to bind mCherry specifically. Consistent with this, the vast majority of beads coated with RFP traps were decorated with a specific mCherry signal upon incubation with Pex3-mCherry supernatants (RFP line/columns B and C on Fig 1E and 1F, and S2B Fig). These mCherry positive beads were negative for BFP labeling (BFP line/columns A, B, and C on Fig 1E and 1F, and S2B Fig), confirming that immunoprecipitated peroxisomes are not bound to mitochondria. However, among RFP beads incubated with Pex3-mCherry/Fzo1-GFP/mito-BFP supernatants, 27% were positive for GFP labeling when the supernatant came from WT cells and this GFP staining reached 45% when the supernatant came from *mdm30Δ* cells (GFP line/columns B and C on Fig 1E and 1F). Quantification of the GFP fluorescence intensity on RFP-positive beads revealed that peroxisomal Fzo1-GFP decreases by 80% in WT as compared to *mdm30Δ* cells (Fig 1G). Importantly, these specific GFP signals were totally washed away upon incubation of the beads with detergent-containing buffers (Fig 1H and S2C Fig). These results confirm that Fzo1 localizes to peroxisomes in *mdm30Δ* cells but also on peroxisomes from *WT* cells.

### Fzo1 is a natural PerMit tether in WT cells

**Fig 2:**
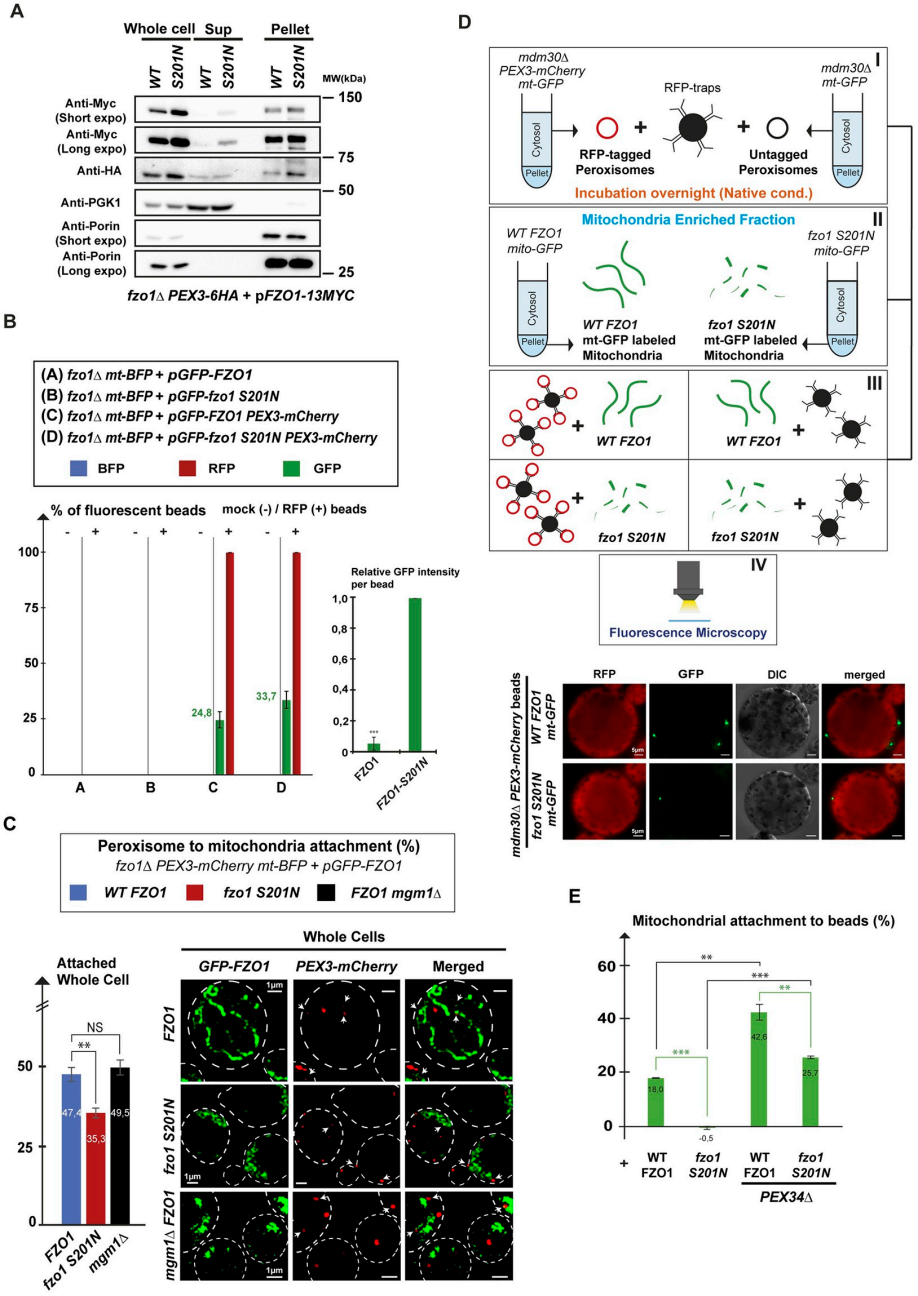
Fzo1 is a natural PerMit tether in WT cells. **(A)** *WT* and *FZO1-S201N* cells labeled for *PEX3-6HA*, and *FZO1-13MYC* (*MCY1779* and *MCY1780*) were processed for fractionation assays to yield whole cell (Total), cytosol (Sup), and membrane (Pellet) fractions that were analyzed by western blotting with anti-Myc, anti-HA, anti-PGK, and anti-Porin. Short and long exposures of anti-Myc and anti-Porin WB are shown. Molecular weights in kDa are indicated on the right. **(B)** Native IP of peroxisomes from the cytosolic fraction of *WT* and *FZO1-S201N* cells labeled for *mt-BFP* and *FZO1-GFP* only or for both *PEX3-mCherry* and *FZO1-GFP* (*MCY1802*, *MCY1803*, *MCY1804*, *MCY1805*) with mock (−) or RFP (+) Trap beads. After analysis by fluorescence microscopy, the left graph depicts the percentage of beads with BFP (blue), mCherry (red), or GFP (green) fluorescence. The readouts for this experiment were identical to those from *mdm30Δ* conditions shown in Fig 1E, S2A and S2B Fig. The right graph depicts the average GFP fluorescence intensity per GFP positive beads in *WT* as compared to *mdm30Δ* conditions. Error bars represent the SEM from 3 independent experiments. **(C)** Fluorescence microscopy of Whole Cells and Percentage of attachment of peroxisomes (mCherry signals) to mitochondria (GFP/BFP signals) in *WT* (blue bars), *FZO1-S201N* (red bars), and *mgm1Δ* (black bars) strains labeled for both *PEX3-mCherry* and *FZO1-GFP* (*MCY1771*, *MCY1772*, and *MCY2112*). White arrows indicate peroxisomes in contact with mitochondria. Error bars represent the SEM from 3 independent experiments. **P < 0.05 (one-way analysis of variance (ANOVA)). NS, not significant. **(D)** Ex vivo PerMit contact assays. Cytosolic fractions of *mdm30Δ* mito-*GFP* cells either genomically labeled or unlabeled for *PEX3-mCherry* (*MCY1842* and *MCY1847* cured from the *MDM30* shuffle plasmid) were incubated with RFP Trap beads overnight and verified by fluorescence microscopy (see S3A Fig) after washing (Panel I). In parallel, diluted membrane fractions from *WT FZO1* or *FZO1-S201N* cells genomically labeled with *mito-GFP* (*MCY1843* transformed with *pRS314-FZO1* (*MC250*) or *pRS314-FZO1-S201N* (*MC544*)) were prepared (Panel II) and incubated overnight with mCherry positive or negative beads from Panel I (Panel III). After washing, mCherry positive or negative beads (see S3B Fig) were analyzed by DIC and fluorescence microscopy (Panel IV). **(E)** Analysis of ex vivo PerMit contact assays. The graph depicts the percentage of specific mitochondrial attachment to beads as compared to all beads analyzed (at least 100) upon incubation with *WT FZO1* or *FZO1-S201N* mitochondria purified from WT or *pex34Δ* cells. Error bars represent the SEM from 3 independent experiments. **P < 0.05, ***P < 0.005 (one-way analysis of variance (ANOVA)). Underlying data for quantifications can be found in S1 Data. Uncropped scans are depicted in S1 Raw Images. BFP, blue fluorescent protein; DIC, differential interference contrast; GFP, green fluorescent protein; RFP, red fluorescent protein.

Its peroxisomal localization in WT cells suggests that Fzo1 likely tethers mitochondria to peroxisomes in physiological conditions. Abolishing the membrane tethering capacity of Fzo1 may thereby decrease PerMit contacts as compared to WT cells. In this regard, mutations in the GTPase domain not only block oligomerization properties of Fzo1 but also inhibit its binding to Mdm30 [23,24,28]. The GTPase mutant Fzo1 S201N is thus stabilized even in the presence of Mdm30 [23,24]. Consistent with this, we observed that the level of extra-mitochondrial Fzo1 S201N is increased as compared to WT Fzo1 in cytosolic supernatants obtained from fractionation of *MDM30* positive cells (Fig 2A). Moreover, immunoprecipitation of native peroxisomes from cytosolic fractions confirmed that the amount of Fzo1 at peroxisomes also increases by more than 80% upon mutation of the GTPase domain (Fig 2B). This led to analyze PerMit contacts in *WT* as compared to *FZO1 S201N* whole cells labeled with Pex3-mCherry and GFP-Fzo1 (Fig 2C). Strikingly, mutation of the GTPase domain decreased contacts by about 12% (Fig 2C) which was confirmed in mitochondrial enriched fractions with a decrease of about 7% (S2D Fig). Confirming that this decrease is not caused by abrogation of mitochondrial fusion and aberrant mitochondrial morphology, inactivation of *MGM1*, the GTPase involved in inner membrane fusion, did not affect PerMit contacts (Fig 2C). These results indicate that when Fzo1 is not functional, its capacity to mediate PerMit contacts is affected. Fzo1 is involved in 7% to 12% of all PerMit contacts in WT cells confirming that Fzo1 is a natural tether between peroxisomes and mitochondria.

Similar to mitochondrial tethering, PerMit contacts mediated by Fzo1 require integrity of its GTPase domain. This strongly suggests that peroxisomal Fzo1 may connect with mitochondrial Fzo1 to trigger PerMit tethering. We reasoned that this could be tested by evaluating the capacity of peroxisomes from *mdm30Δ* cells with increased amounts of WT Fzo1 to bind mitochondria purified from either *WT* or *FZO1 S201N* cells. We therefore incubated RFP traps in native conditions with the cytosolic fractions of *mt-GFP PEX3-mCherry mdm30Δ* cells or *mt-GFP mdm30Δ* cells (Fig 2D, Panel I). As expected, RFP-trap beads were decorated with mCherry only after incubation with PEX3-mCherry supernatants (S3A Fig). Most importantly, GFP signals were not detected on the beads (S3A Fig) which further confirmed that peroxisomes purified from cytosolic fractions are not bound to mitochondria (Fig 1E). Subsequently, we incubated mCherry-positive and negative RFP traps with mitochondrial-enriched fractions from *mt-GFP* or *mt-GFP FZO1 S201N* cells that were prepared extemporaneously (Fig 2D, Panels II and III). After washing steps, fluorescence microscopy analysis allowed detecting nonspecific GFP signals on Pex3-mCherry negative beads (S3B Fig) that were subtracted from specific GFP signals seen on Pex3-mCherry positive beads (Fig 2D, bottom). Upon incubation with *WT FZO1* mitochondria, the ratio of specific GFP staining over the total number of mCherry positive beads was of 18% (Fig 2E). In contrast, specific GFP staining of the beads was totally abolished upon incubation with *FZO1 S201N* mitochondria (Fig 2E). These results indicate that the PerMit tethering mediated by WT peroxisomal Fzo1 is abolished upon mutation of the GTPase domain on mitochondrial Fzo1. They also suggest that all PerMit contacts that are formed ex vivo are mediated by Fzo1. To explain this counter-intuitive result, we reasoned that all PerMit tethers that are distinct from Fzo1, including the peroxisomal membrane protein Pex34 [10], may already saturate their respective mitochondrial acceptor sites in WT mitochondrial enriched fractions. If true, this should leave mitochondrial Fzo1 as the only available PerMit tether in ex vivo PerMit contact assays. We thus repeated the experiment with mitochondrial enriched fractions prepared from *pex34Δ* cells. This increased overall specific GFP staining by 25% but maintained a 17% decrease of mitochondria-peroxisome attachment upon mutation of the Fzo1 GTPase domain (Fig 2E). Peroxisomal Fzo1 thus associates with mitochondrial Fzo1 to promote PerMit tethering.

### Fzo1-mediated PerMit contacts are regulated by FA desaturation

**Fig 3:**
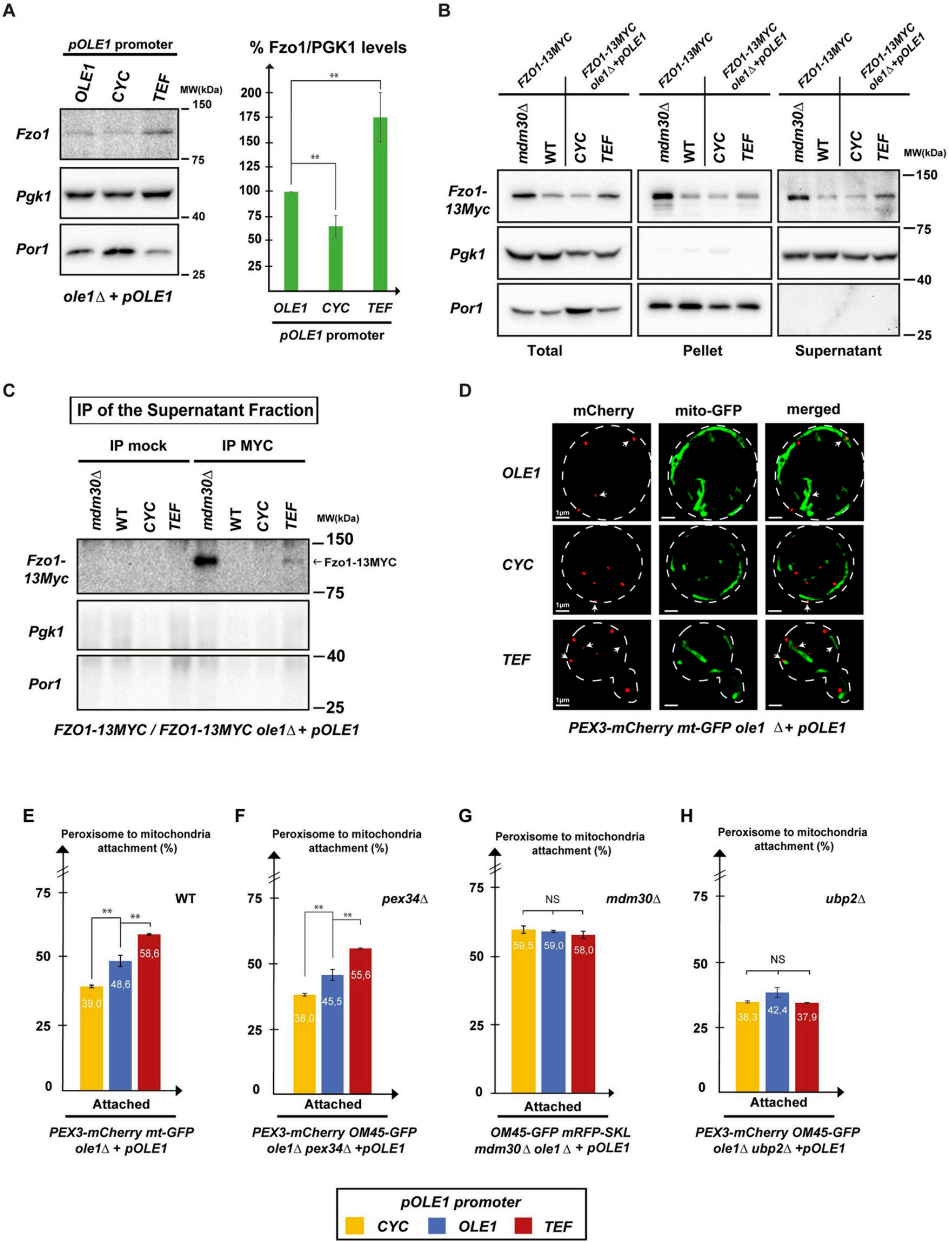
Fzo1-mediated PerMit contacts are regulated by FA desaturation. **(A)** Whole cell extracts of *ole1Δ* strains shuffled with *OLE1*, *CYC*, or *TEF pOLE1* plasmids (*MCY1861*, *MCY1863*, *MCY1865*) were processed for western blotting with anti-Fzo1, anti-PGK, and anti-Porin. Molecular weights in kDa are indicated on the right. The Fzo1/PGK1 ratio in each condition was then quantified relative to the *OLE1* promoter condition. Error bars represent the SEM from 3 independent experiments. **P < 0.05 (one-way analysis of variance (ANOVA)). **(B and C)** *WT*, *mdm30Δ*, and *ole1Δ* cells shuffled with *CYC* or *TEF pOLE1* plasmids, genomically labeled for *FZO1-13MYC* (*MCY1488*, *MCY1490*, and *MCY1785* transformed with *pRS414-CYC-OLE1* (*MC540*) or *pRS414-TEF-OLE1* (*MC541*)) were processed for fractionation assays to yield Total (whole cell), Supernatant (cytosol + free peroxisomes or vesicules), and Pellet (membrane) fractions (B). Supernatant fractions were subsequently processed for denaturating IPs with anti-Myc or mock beads (C). All fractions and IPs were analyzed by western blotting with anti-Myc, anti-PGK1, and anti-Porin. Molecular weights in kDa are indicated on the right of the blots. **(D)** *ole1Δ* strains genomically labeled for *PEX3-mCherry* and *mito-GFP* were shuffled with *OLE1*, *CYC*, or *TEF pOLE1* plasmids (*MCY1861*, *MCY1863*, *MCY1865*) and processed for whole cells imaging by SIM. White arrows indicate peroxisomes in contact with mitochondria. Scale bars for whole cells (delimited in white) correspond to 1 μm. **(E)** The graph depicts the percentage of attachment of peroxisomes (mCherry signals) to mitochondria (GFP signals) in *CYC-OLE1* (yellow bars), *OLE1* (blue bars), and *TEF-OLE1* (red bars) cells from (D). Error bars represent the SEM from 3 independent experiments. **P < 0.05 (one-way analysis of variance (ANOVA)). NS, not significant. **(F, G, and H)** Same as (E) but in cells inactivated for *PEX34* (F), *MDM30* (G), or *UBP2* (H) (*MCY2101*, *MCY1959*, or *MCY2091* shuffled with *pRS414-CYC-OLE1* (*MC540*) or *pRS414-TEF-OLE1* (*MC541*) or *pRS414-OLE1-OLE1* (*MC543*)). Underlying data for quantifications can be found in S1 Data. Uncropped scans are depicted in S1 Raw Images. FA, fatty acid; GFP, green fluorescent protein; SIM, structured illumination microscopy.

We have previously demonstrated that increased FA desaturation induces a natural stabilization of Fzo1 by slowing down its Mdm30-dependent degradation [14]. In this context, increased unsaturated fatty acids (UFAs) should stabilize Fzo1 which may induce its physiological accumulation on peroxisomes. However, the addition of extracellular UFAs promotes complex feedback loops on the yeast FA desaturase Ole1, which results in decreased production of endogenous UFAs [18,21,29]. To bypass this issue, we designed *OLE1* shuffle strains in which Ole1 is expressed under the control of distinct promoters with low (*CYC* or *MET* promoters) to strong (*ADH* or *TEF* promoters) transcription activation (S3C–S3E Fig). As compared to the WT control with the *OLE1* promoter, high expression of Ole1 from the *TEF* promoter induced accumulation of endogenous Fzo1 with concomitant decrease in the mitochondrial Porin (Fig 3A). In contrast, low expression of Ole1 from the *CYC* promoter resulted in decreased levels of Fzo1 with concomitant increase in Porin (Fig 3A). These results reveal that FA desaturation regulates the level of Porin but are also consistent with our previous findings demonstrating that high or low FA desaturation promote stabilization or degradation of Fzo1, respectively [14].

We employed this system to assess the extra-mitochondrial localization of Fzo1 according to the level of Ole1 expression. Fractionation assays with *WT*, *OLE1 CYC*, *OLE1 TEF*, and *mdm30Δ* cells, all genomically tagged with *FZO1-13MYC*, revealed that high Ole1 expression from the *TEF* promoter induces an accumulation of Fzo1-13Myc in the supernatant devoid of mitochondria (Fig 3B). Anti-Myc IPs of supernatants confirmed this increased presence of Fzo1-13Myc in the supernatant fraction of cells overexpressing Ole1 (Fig 3C), suggesting that high FA desaturation promotes an accumulation of Fzo1 on peroxisomes. This led to evaluate PerMit contacts in *OLE1*, *CYC-OLE1*, and *TEF-OLE1* cells (Fig 3D). Modulation of Ole1 expression did not impact the number of peroxisomes per cell (S3F Fig). However, consistent with the increased level of Fzo1 upon Ole1 overexpression, PerMit contacts increased by 10% in *TEF-OLE1* as compared to *OLE1* cells (Fig 3E). Moreover, lower level of Fzo1 in *CYC-OLE1* cells correlated with a 10% decrease in PerMit contacts as compared to the *OLE1* control (Fig 3E). PerMit contacts thus not only increase with the status of FA desaturation but also with the level of Fzo1.

To evaluate the role and specificity of Fzo1 in this response to FA desaturation, PerMit contacts were analyzed in cells lacking either Pex34, Mdm30, or Ubp2. Inactivation of *PEX34* logically induced a faint decrease in all PerMit contacts but had no effect on the responses to FA desaturation (Fig 3F). In contrast, inactivation of *MDM30* resulted in increased PerMit contacts regardless of the expression level of *OLE1* (Fig 3G). Conversely, the absence of *UBP2* also abolished the response to FA desaturation but induced an overall decrease in PerMit contacts at the level seen in the CYC condition (Fig 3H). Taken together, these observations confirm that the responses of PerMit contacts to FA desaturation strictly depend on Fzo1 but not on other PerMit tethers such as Pex34. Most importantly, they indicate that Fzo1-mediated PerMit contacts are naturally regulated by FA desaturation and Mdm30-mediated degradation of Fzo1.

### Fzo1 rescues the respiratory growth of *mdm30Δ* cells

**Fig 4:**
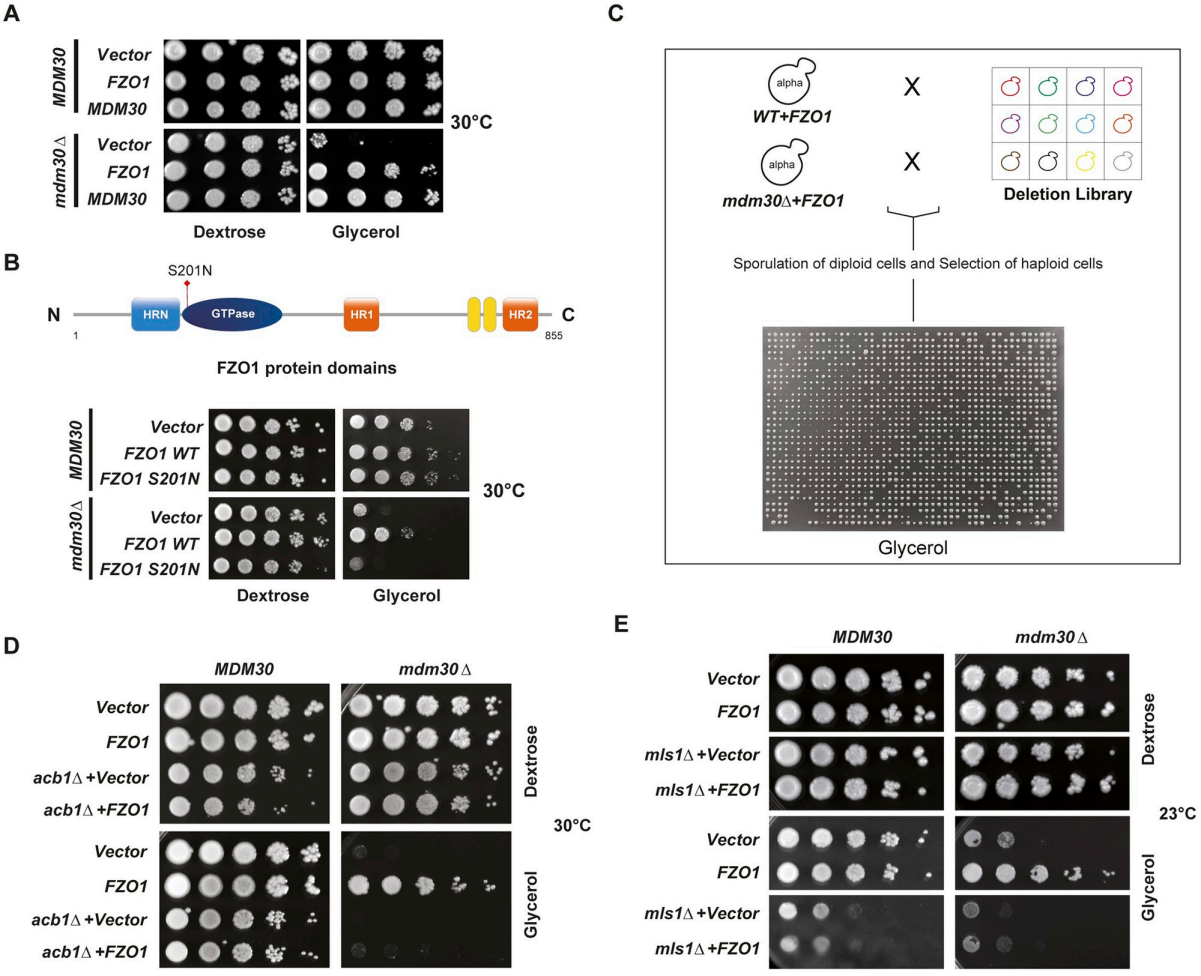
Fzo1 rescues the respiratory growth of *mdm30*Δ cells. **(A)** Dextrose and glycerol spot assays at 30 °C of *MDM30* shuffling strains (*MCY971*) transformed with *pRS314-FZO1* (*MC250*), *pRS314-MDM30* (*MC344*), or an empty vector (*MC219*) and covered by (*MDM30*) or cured from (*mdm30Δ*) the *MDM30* shuffle plasmid. **(B)** Top: Domain organization of Fzo1 (residues 1 to 855 from the N- to the C-terminal extremity) with the GTPase domain (dark blue), the Heptad Repeats HRN (light blue), HR1 and HR2 (orange), the Trans-membrane domains (yellow), and the S201N mutation (red arrow). Bottom: Same as (A) but with *MDM30* shuffling strains (*MCY970*) transformed with *pRS314-FZO1* (*MC250*), *pRS314-FZO1 S201N* (*MC544*), or an empty vector (*MC219*). **(C)** High-throughput screen for deletion candidates that abolish the respiratory rescue of *mdm30Δ* cells by Fzo1. *WT* (*MCY1513*) and *mdm30Δ* (*MCY1528*) cells transformed with *pRS416-FZO1* (*MC322*) were crossed with the deletion library SGA::G418 and resulting diploid cells were sporulated. Following appropriate selection (see Materials and methods), single (*MDM30 xxxΔ*), and double deletion (*mdm30Δ xxxΔ*) haploid cells were grown for 7 days on glycerol containing media. Candidates of interest were characterized as deletions that decreased growth of the double mutant as compared to the single mutant. **(D and E)** Confirmed candidates after the secondary screen. Dextrose and glycerol spot assays at 30 °C (D) and 23 °C (E) of *MDM30* (*MCY970*), *MDM30 acb1Δ* (*MCY1612*), and *MDM30 mls1Δ* (*MCY1649*) shuffling strains transformed with *pRS314-FZO1* (*MC250*) or an empty vector (*MC219*) and covered by (*mdm30Δ + MDM30*) or cured from (*mdm30Δ*) the *MDM30* shuffle plasmid. Full plates are shown in S4C Fig.

With the objective of investigating the function of Fzo1-mediated PerMit contacts, we turned to a genetic approach motivated by an unexpected observation (Fig 4A). The absence of Mdm30 induces decreased respiratory growth on media supplemented with glycerol as the only source of carbon [22,30]. Yet, we serendipitously observed that increased expression of Fzo1 through addition of a plasmid encoding for an extra copy of *FZO1* induces a significant rescue of this phenotype at 30 °C (Fig 4A). This unexpected rescue requires a functional copy of Fzo1 as the *S201N* mutation inhibited the effect of extra Fzo1 (Fig 4B). We also observed that the extra copy rescues the otherwise abolished growth of mdm30 null cells on Oleate media (S4A Fig), suggesting that the extra copy may improve peroxisomal function. The extra copy of Fzo1 may favor the stability of Fzo1-mediated PerMit contacts and enhance their function, which would result in the improved respiratory growth we observed. We thus launched an unbiased genetic screen to seek for genes that participate in the improved respiratory growth of *mdm30Δ* cells by the extra-*FZO1*.

An extra copy of *FZO1* on either a *WT* or *mdm30Δ* background was integrated into a collection of strains representing deletions in the majority of all yeast non-essential genes (Fig 4C). *MDM30* and *mdm30Δ* haploid strains carrying the extra copy of *FZO1* and an additional single deletion were grown for 7 days on glycerol media (that does not allow growth in the absence of a functional respiratory capacity) and positive hits were defined as candidates that maintained growth in the presence but not in the absence of *MDM30* (see Materials and methods). This primary screen provided several hits that were subsequently verified in a secondary screen where deletion candidates were reintroduced in *MDM30* shuffle cells, in the presence or in the absence of the extra copy of *FZO1*. After *MDM30* curing on 5-FOA (see Materials and methods), a true hit was expected to induce strong inhibition of the respiratory rescue of *mdm30Δ* cells by extra-*FZO1* but have more limited effects on *MDM30* positive cells. Besides numerous false positive hits (S4B Fig), this secondary screen yielded only 2 genes, *ACB1* and *MLS1*, that fulfilled the conditions required for a true positive candidate (Fig 4D and 4E, and S4C Fig). *ACB1* encodes a fatty-acyl-CoA binding protein that is involved in the biosynthesis of long-chain fatty acids [31]. Most importantly, Mls1 is the malate synthase enzyme involved in the peroxisomal glyoxylate cycle [32]. Inactivation of either *ACB1* or *MLS1* decrease growth of *MDM30* positive cells on glycerol media but nearly abolishes the respiratory rescue of *mdm30Δ* cells by the extra copy of Fzo1 at 30 or 23 °C, respectively (Fig 4D and 4E, and S4C Fig). These observations resulting from an unbiased genetic screen point again to FAs with *ACB1* and peroxisomes with *MLS1*.

### Mls1 and Icl1 regulate mitochondrial morphology and respiration

**Fig 5:**
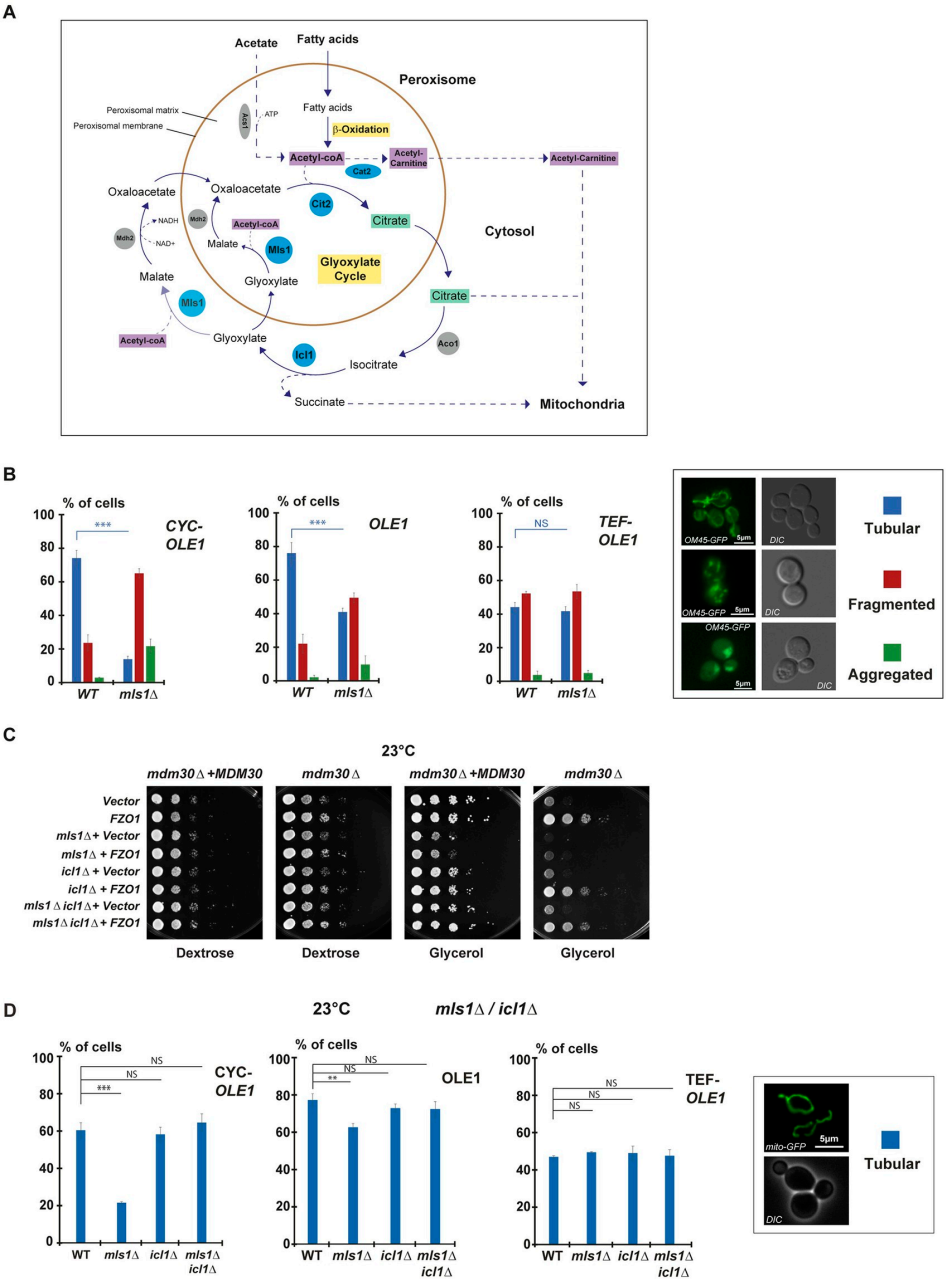
Inactivation of *ICL1* bypasses the effects of *MLS1* inactivation. **(A)** The Glyoxylate cycle in *S*. *cerevisiae*. See main text. All enzymes inactivated in this study are in blue. Beta-oxidation and the glyoxylate cycle are the 2 pathways involved in peroxisomal FA catabolism and are highlighted in yellow. Citrate (highlighted in green) generated by Cit2, Acetyl-CoA (highlighted in purple) in the form of Acetyl-Carnitine generated by Cat2 and Succinate generated by Icl1, can all transit to mitochondria. **(B)** Right: examples of cells genomically labeled for *OM45-GFP* (*MCY1899*) with tubular (blue), fragmented (red) or aggregated (green) mitochondrial networks; scale bar, 5 μm. Left: Percentage of cells with tubular (blue), fragmented (red) or aggregated (green) mitochondria from *OLE1* (*WT*) and *OLE1 mls1Δ* (*mls1Δ*) shuffling strains genomically labeled for *OM45-GFP* and *RFP-SKL* and shuffled with *OLE1*, *CYC*, or *TEF pOLE1* plasmids (*MCY1899*, *MCY1980*, *MCY1987*). Error bars represent the SEM from 3 independent experiments. **P < 0.05, ***P < 0.005 (one-way analysis of variance (ANOVA)). NS, not significant. More than 100 cells were analyzed per sample. **(C)** Dextrose and glycerol spot assays at 23 °C of *MDM30 mls1Δ* (*MCY1649)*, *MDM30 icl1Δ* (*MCY1909*), and *MDM30 mls1Δ icl1Δ* (*MCY1911*) shuffling strains transformed with *pRS314-FZO1* (*MC250*) or an empty vector (*MC219*) and covered by (*mdm30Δ + MDM30*) or cured from (*mdm30Δ*) the *MDM30* shuffle plasmid. **(D)** Right: Examples of cells genomically labeled for *mito-GFP* (*MCY1835*) with tubular mitochondrial networks; scale bar, 5 μm. Left: Percentage of cells with tubular mitochondria at 23 °C from *OLE1* (*WT*), *OLE1 mls1Δ* (*mls1Δ*), *OLE1 icl1Δ* (*icl1Δ*), and *OLE1 mls1Δ icl1Δ* (*mls1Δ icl1Δ*) shuffling strains genomically labeled for *mito-GFP* and shuffled with *OLE1*, *CYC*, or *TEF pOLE1* plasmids (*MCY1835*, *MCY1989*, *MCY2002*, *MCY2003*). Error bars represent the SEM from 3 independent experiments. **P < 0.05, ***P < 0.005 (one-way analysis of variance (ANOVA)). NS, not significant. More than 100 cells were analyzed per sample. Underlying data for quantifications can be found in S1 Data. FA, fatty acid; GFP, green fluorescent protein.

Acb1 is thought to transport newly synthesized acyl-CoA esters to acyl-CoA-consuming processes [31,33]. While such transport may occur through peroxisomes, it remains to be demonstrated for Acb1. This makes the impact of *ACB1* inactivation on respiratory growth hazardous to interpret. Conversely, Mls1 is an established enzyme of the glyoxylate cycle (Fig 5A). Mls1 is either localized in peroxisomes or in the cytosol depending on the carbon source utilized for growth [34]. In either case, the precise function of Mls1 consists in generating Malate from Glyoxylate that is itself generated by Icl1, the isocitrate lyase strictly localized in the cytosol (Fig 5A).

We aimed at understanding the functional link between Mls1, Fzo1-mediated PerMit contacts and mitochondrial homeostasis. Importantly, this link cannot be evaluated by directly inactivating or overexpressing Fzo1 that would inhibit mitochondrial fusion and would consequently be detrimental for all mitochondrial functions. However, modulation of Ole1 expression levels regulate the amount of PerMit contacts mediated by Fzo1 (Fig 3E–3H) which provides an ideal mean to investigate their physiological role. We thus analyzed PerMit contacts and mitochondrial morphology in cells lacking Mls1 upon low, normal, or high expression of Ole1. Inactivation of *MLS1* did not affect the response of PerMit contacts to the status of FA desaturation (S5A Fig). However, significant changes in mitochondrial morphology were observed in *MLS1* negative as compared to *WT* cells, depending on the expression level of *OLE1* (Fig 5B; *CYC*, *OLE1*, *TEF*). During low expression of *OLE1* from the *CYC* promoter (Fig 5B; *CYC*), absence of *MLS1* induced a drastic decrease in the number of cells with tubular mitochondria (75% in *WT* versus 15% in mutant cells) and a concomitant increase in mitochondria that are fragmented. Upon expression of *OLE1* from its own promoter (Fig 5B; *OLE1*), the decrease in tubular mitochondria persisted but to a lesser extent (75% in *WT* versus 40% in mutant cells). Interestingly, overexpression of *OLE1* from the *TEF* promoter (Fig 5B; *TEF*) affected mitochondrial morphology with only 40% of WT cells with tubular networks (compare *WT* cells in *CYC*, *OLE1*, and *TEF* conditions). Yet, the deletion of *MLS1* did no longer induce any decrease in tubular mitochondria (40% in *WT* versus 40% in mutant cells in the *TEF* condition). These results reveal an impact of *MLS1* inactivation on mitochondrial dynamics. However, when FA desaturation and Fzo1-mediated PerMit contacts increase (S5A Fig), the specific impact of *MLS1* deletion on mitochondrial networks decreases.

Inactivation of *MLS1* would be expected to block the production of Malate while inducing accumulation of Glyoxylate and Succinate, both generated by Icl1 (Fig 5A). In turn, the accumulation of Icl1 byproducts, the consumption of Icl1 substrates or the inhibition of Malate synthesis may affect mitochondrial homeostasis. Yet, this does not explain the protection on mitochondrial morphology seen upon high FA desaturation. This led us testing the effect of *ICL1* inactivation on respiratory growth (Fig 5C and S4D Fig) and mitochondrial morphology (Fig 5D and S5B Fig). As opposed to the inactivation of *MLS1*, the absence of *ICL1* did not affect the glycerol growth rescue of *MDM30* null cells by the extra copy of Fzo1 (Fig 5C), indicating that inhibition of Malate production does not affect this rescue (Fig 5A). Most interestingly, inactivation of *ICL1* induced a striking reversion of the effect resulting from *MLS1* deletion at both 23 and 30 °C (Fig 5C and S4D Fig). As expected from glycerol growth assays (Fig 5C), inactivation of *ICL1* also induced a striking reversion of the mitochondrial morphology defects resulting from *MLS1* deletion at both 23 and 30 °C (Fig 5D and S5B Fig). The amount of tubular mitochondrial networks in *icl1Δ* cells at 23 °C was indiscernible from the morphology of mitochondrial networks in *WT* cells but also when *ICL1* was co-inactivated with *MLS1* (*mls1Δ icl1Δ* cells) in *CYC-OLE1*, in *OLE1*, or in *TEF-OLE1* conditions (Fig 5D). These results indicate that Icl1 is causal in the mitochondrial morphology defects seen in the absence of *MLS1* when Fzo1-mediated PerMit contacts decrease. The absence of Icl1 abolishes the effects of *MLS1* inactivation upon low or normal *OLE1* expression, demonstrating that these effects are not a consequence of decreased FA desaturation but are rather caused by the decrease in Fzo1-mediated PerMit contacts. In this context, increased Fzo1-mediated PerMit contacts may explain the protection on mitochondrial morphology seen upon *MLS1* deletion in the *TEF-OLE1* condition (Fig 5B and 5D; *TEF-OLE1*). Consistent with this, co-inactivation of *MLS1* and *ICL1* improved the tubular morphology of *TEF-OLE1* cells at 30 °C (S5B Fig), whereas increased FA desaturation affects mitochondrial morphology in WT cells at both 23 and 30 °C (Fig 5B and 5D, and S5B Fig). Fzo1-mediated PerMit contacts may bypass Icl1 and favor the mitochondrial transfer of early byproducts from the Glyoxylate cycle upstream of Icl1 resulting in maintenance of tubular mitochondrial morphology.

### Synthesis of peroxisomal citrate regulates mitochondrial morphology

**Fig 6:**
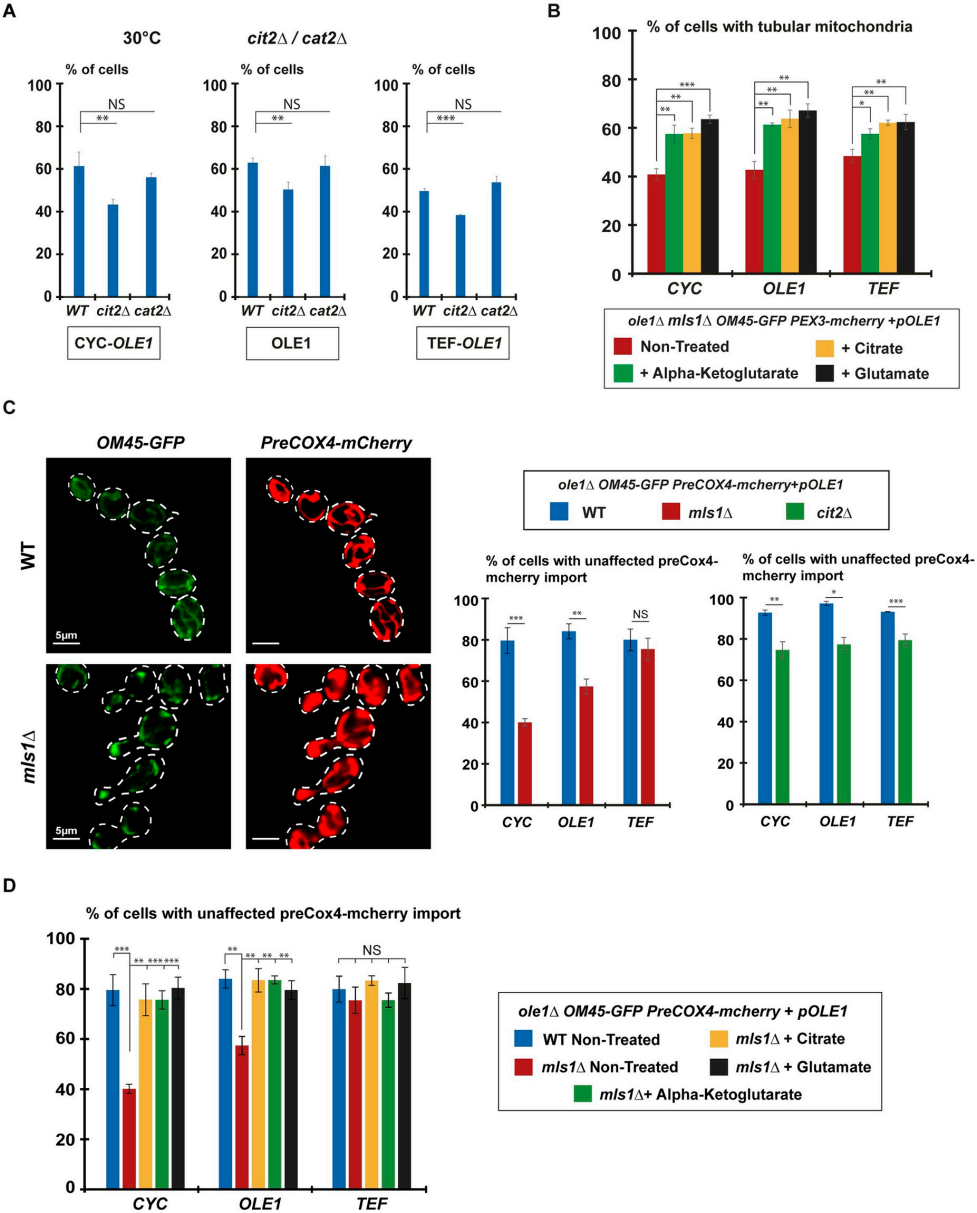
Feeding the TCA cycle rescues mitochondrial morphology and membrane potential in *mls1*Δ cells. **(A)** Percentage of cells with tubular mitochondria at 30 °C from *OLE1* (*WT*), *OLE1 cit2Δ* (*cit2Δ*), and *OLE1 cat2Δ* (*cat2Δ*) shuffling strains genomically labeled for *mito-GFP* and shuffled with *OLE1*, *CYC*, or *TEF pOLE1* plasmids (*MCY1835*, *MCY2023*, *MCY2032*). Error bars represent the SEM from 3 independent experiments. **P < 0.05, ***P < 0.005 (one-way analysis of variance (ANOVA)). NS, not significant. More than 100 cells per sample were analyzed. **(B)** Percentage of cells with tubular mitochondria from *OLE1 mls1Δ* shuffling strains genomically labeled for *OM45-GFP* and shuffled with *OLE1*, *CYC*, or *TEF pOLE1* plasmids (*MCY1987*). Cells were grown in media supplemented with citrate (yellow bars), alpha-ketoglutarate (green bars), or glutamate (black bars) as indicated. Error bars represent the SEM from 3 independent experiments. *P = 0.05, **P < 0.05, ***P < 0.005 (one-way analysis of variance (ANOVA)). More than 100 cells per sample were analyzed. **(C)** Left images: Examples of *WT* (*MCY2080*) and *mls1Δ* (*MCY2084*) cells genomically labeled for *OM45-GFP* (green) and *preCOX4-mCherry* (red) co-labeling the mitochondrial network; scale bar 5 μm. A diffuse preCox4-mCherry signal is present in the cytoplasm of *mls1Δ* cells reflecting defects in mitochondrial import and a decrease in the *ΔΨ*. Right graphs: Percentage of cells with unaffected mitochondrial import of preCox4-mCherry at 30 °C in *OLE1* (*WT*, blue bars), *OLE1 mls1Δ* (*mls1Δ*, red bars), and *OLE1 cit2Δ* (*cit2Δ*, green bars) shuffling strains genomically labeled for *OM45-GFP* and preCOX4-mCherry and shuffled with *OLE1*, *CYC*, or *TEF pOLE1* plasmids (*MCY2080*, *MCY2084*). Error bars represent the SEM from 3 independent experiments. **P < 0.05, ***P < 0.005 (one-way analysis of variance (ANOVA)). NS, not significant. More than 100 cells per sample were analyzed. **(D)** Same as (A) with citrate (yellow bars), alpha-ketoglutarate (green bars), or glutamate (black bars) supplemented to *mls1Δ* cells. Error bars represent the SEM from 3 independent experiments where all samples have been treated at the same time. **P < 0.05, ***P < 0.005 (one-way analysis of variance (ANOVA)). NS, not significant. More than 100 cells per sample were analyzed. Underlying data for quantifications can be found in S1 Data. GFP, green fluorescent protein; TCA, tricarboxylic acid.

In the early steps of the Glyoxylate cycle [35,36], the peroxisomal enzyme Cit2 generates citrate from Acetyl-CoA and Oxalo-acetate (Fig 5A). Citrate is then transported to the cytosol where it is converted into Isocitrate that is subsequently used by Icl1 to generate Glyoxylate and Succinate (Fig 5A). Notably, citrate can also be redirected to mitochondria, similarly to peroxisomal Acetyl-CoA that can exit peroxisomes in the form of Acetyl-Carnitine generated by the peroxisomal enzyme Cat2 (Fig 5A). Increased Fzo1-mediated PerMit contacts may transfer citrate or Acetyl-Carnitine to mitochondria before Icl1 can be reached, which would maintain mitochondrial morphology (Fig 5B and 5D, and S5B Fig). We reasoned that if Fzo1-mediated PerMit contacts allow the transfer of citrate or Acetyl-CoA to mitochondria, the inactivation of *CIT2* or *CAT2* should impact mitochondrial morphology in *CYC-OLE1* and *OLE1* but also in the more robust *TEF-OLE1* condition. While inactivation of *CAT2* had no effect as compared to WT cells, the absence of *CIT2* induced a decrease in tubular mitochondria in *CYC-OLE1* and *OLE1* cells and most remarkably in the *TEF-OLE1* condition (Fig 6A). This implies that citrate enters mitochondria which may require the Ctp1 transporter located in inner membranes [37–40]. Consistent with this, inactivation of *CTP1* induced the same effects as deletion of *CIT2* on the amounts of tubular mitochondria (S5C Fig). Specifically blocking the synthesis of peroxisomal citrate or its transfer to mitochondria therefore likely affects the capacity of Fzo1-mediated PerMit contacts to regulate mitochondrial dynamics upon high FA desaturation as these increased contacts were maintained in the absence of either *CIT2* or *CAT2* (S5D and S5E Fig).

Once transported to mitochondria, citrate may feed the oxidative TCA cycle which would favor maintenance of mitochondrial homeostasis. In support of this model, we observed that addition of exogenous substrates of the TCA cycle, including citrate, alpha-ketoglutarate, or even glutamate that generates alpha-ketoglutarate [41], induced significant rescue of tubular mitochondrial morphology in the absence of *MLS1* upon low, normal and even high *OLE1* expression (Fig 6B). This demonstrates that impaired fueling of the TCA cycle with citrate is the main causality in defective mitochondrial morphology and homeostasis in the absence of Mls1.

### Peroxisomal citrate enters the TCA cycle to stimulate the mitochondrial membrane potential

Feeding the TCA cycle with citrate stimulates the mitochondrial membrane potential [42]. In this context, the impacts of *MLS1* or *CIT2* inactivation on mitochondrial morphology could be caused by perturbations of the *ΔΨ* because of defective citrate import. We addressed this possibility employing the preCox4-mCherry *ΔΨ* marker that is imported into mitochondria in WT cells but accumulates in the cytosol upon perturbations of the *ΔΨ* [43]. This readout allowed verifying that FA desaturation does not influence preCox4-mCherry import in WT cells whether in *CYC-OLE1*, *OLE1*, or in *TEF-OLE1* conditions (Fig 6C; WT, blue bars). However, while inactivation of *MLS1* did not affect preCox4-mCherry import in the *TEF-OLE1* condition, this import decreased by 20% and even 40% in the *OLE1* and *CYC-OLE1* conditions, respectively (Fig 6C; *mls1Δ*, red bars). This response to FA desaturation follows the same profile as that observed for mitochondrial morphology in *mls1Δ* cells, which is caused by Icl1 (Fig 4B and 4D). This indicates that Fzo1-mediated PerMit contacts promote maintenance of the *ΔΨ*, presumably by feeding the mitochondrial TCA cycle with citrate synthesized in peroxisomes. Consistent with this, inactivation of *CIT2* decreased preCox4-mCherry import by 15%, regardless of the level of expression of Ole1 (Fig 6C; *cit2Δ*, green bars). Moreover, addition of exogenous substrates of the TCA cycle induced total rescue of preCox4-mCherry import in *OLE1* and *CYC-OLE1 mls1Δ* cells (Fig 6D), again confirming that fueling of the TCA cycle with citrate is the main causality in defective mitochondrial homeostasis upon *MLS1* inactivation.

### Fzo1-mediated PerMit contacts regulate mitochondrial fusion

**Fig 7:**
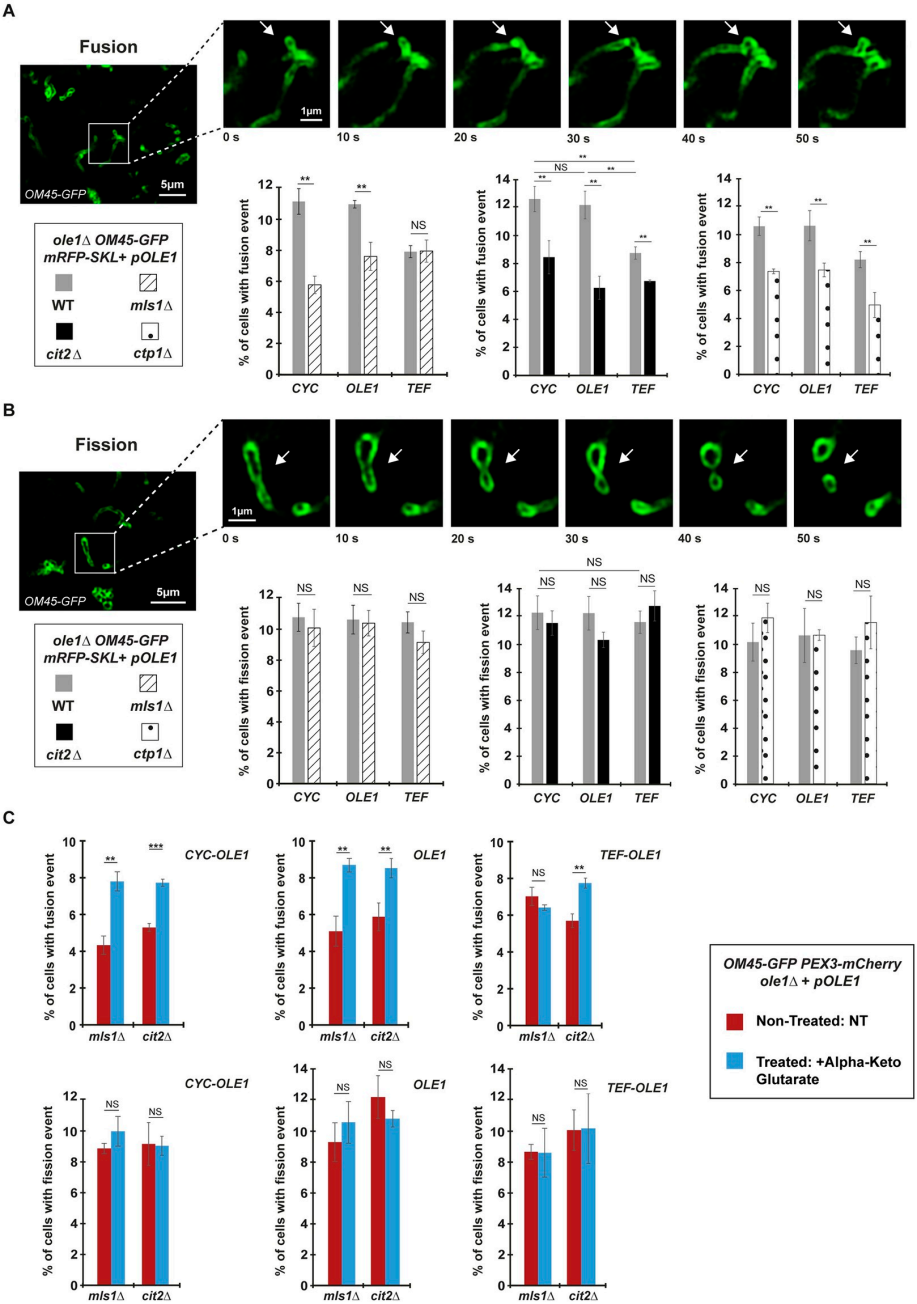
Synthesis and mitochondrial import of peroxisomal citrate regulates mitochondrial fusion. **(A and B)** Top images: Examples of time lapse acquisitions with 10-s intervals (Total duration of 3–5 min) of mitochondrial fusion (A) and fission (B) events (indicated by white arrows) by SIM with cells genomically labeled for *OM45-GFP* (*MCY1936*); scale bars, 5 μm (left fields) or 1 μm (right zooms). Bottom graphs: Percentage of cells with fusion (A) and fission (B) events in *WT* (gray columns), *mls1Δ* (dashed columns), *cit2Δ* (black columns), or *ctp1Δ* (doted columns) strains. Error bars represent the SEM from 3 independent experiments. **P < 0.05 (one-way analysis of variance (ANOVA)). NS, not significant. **(C)** Percentage of cells with fusion (top) and fission (bottom) events in *mls1Δ* and *cit2Δ* strains, Non-Treated (red) or Treated (blue) with alpha-ketoglutarate. Error bars represent the SEM from 3 independent experiments. **P < 0.05 (one-way analysis of variance (ANOVA)). NS, not significant. More than 100 cells per sample were analyzed. Underlying data for quantifications can be found in S1 Data. GFP, green fluorescent protein; SIM, structured illumination microscopy.

The role of Fzo1-mediated PerMit contacts in transferring peroxisomal Citrate into mitochondria to feed the TCA cycle and stimulate the *ΔΨ* likely impacts numerous mitochondrial functions. Among those, mitochondrial fusion is established to rely on the mitochondrial membrane potential [4,44,45]. We consequently asked whether aberrant mitochondrial morphology in *mls1Δ* and *cit2Δ* cells (Figs 5 and 6) could be explained by a specific defect in mitochondrial fusion efficiency. To evaluate this possibility, we noticed that time lapse acquisitions by structured illumination microscopy (SIM) over short durations of 3 to 5 min provides a mean to achieve clear visualization of 1 to 2 mitochondrial fusion (S1 Video) or fission (S2 Video) events within 10% to 12% of cells labeled with OM45-GFP (see Materials and methods). Given the gain in resolution achieved by SIM, these distinct mitochondrial dynamics events are not only caught within tubular or fragmented networks but also within aggregated mitochondria (S6A Fig). We thus employed this approach to quantify the amount of *CYC*, *OLE1*, and *TEF WT* cells with mitochondrial fusion or fission events (Fig 7A and 7B). Consistent with our mitochondrial morphology data (Fig 5B and 5D), we observed an appropriate equilibrium between fusion and fission events frequency in 11% to 12% of *CYC-OLE1* and *OLE1 WT* cells (Fig 7A and 7B; *WT CYC* and *WT OLE1*). In the *TEF-OLE1 WT* condition, cells with fusion events slightly decreased at 8% (Fig 7A; *WT TEF*), whereas fission frequency was unchanged (Fig 7B; *WT TEF*) which agrees with the partial loss of tubular mitochondria when *OLE1* is overexpressed (Fig 5B and 5D).

We pursued this analysis in the same set of cells that we inactivated for *MLS1*, *CIT2*, or *CTP1*. In these mutants, fission frequency was not significantly modified as compared to *WT* conditions in either *CYC-OLE1*, *OLE1*, or *TEF-OLE1* cells (Fig 7B). Upon low expression of *OLE1* and decreased Fzo1-mediated PerMit contacts, the fusion frequency diminished significantly from 11% to 12% in *WT* to 6% to 8% in all mutant cells (Fig 7A; *CYC; mls1Δ*, *cit2Δ*, *ctp1Δ*). A similar decrease in fusion frequency was observed in all *OLE1* mutant cells (Fig 7A; *OLE1; mls1Δ*, *cit2Δ*, *ctp1Δ*). In contrast, inactivation of *MLS1* did not affect mitochondrial fusion in the *TEF-OLE1* condition where Fzo1-mediated PerMit contacts are the highest (Fig 7A; *TEF*; *mls1Δ*). However, inhibition of either peroxisomal citrate synthesis in *cit2Δ* cells or transport to mitochondria in *ctp1Δ* cells induced significantly decreased fusion frequency as compared to WT cells (Fig 7A; *TEF*; *cit2Δ* and *ctp1Δ*). Notably, we confirmed all the effects of *MLS1* inactivation on mitochondrial fusion by more classic in vivo mitochondrial fusion assays (S6B Fig), thus validating our approach to investigate mitochondrial dynamics by time lapse acquisitions through SIM.

Similar to the rescue of mitochondrial morphology (Fig 6B) or *ΔΨ* through preCox4-mCherry import (Fig 6D), we tested the effect of providing exogenous substrates of the TCA cycle on mitochondrial fusion and fission. Exogenous addition of alpha-ketoglutarate in *mls1Δ* and *cit2Δ* cells did not impact mitochondrial fission frequency but rescued mitochondrial fusion in CYC, OLE1, and TEF conditions (Fig 7C). This further confirms that all defects in mitochondrial morphology, *ΔΨ* or mitochondrial fusion seen when Fzo1-mediated PerMit contacts decrease or when mitochondrial transit of peroxisomal citrate is affected can be bypassed by providing substrates to the TCA cycle.

**Fig 8:**
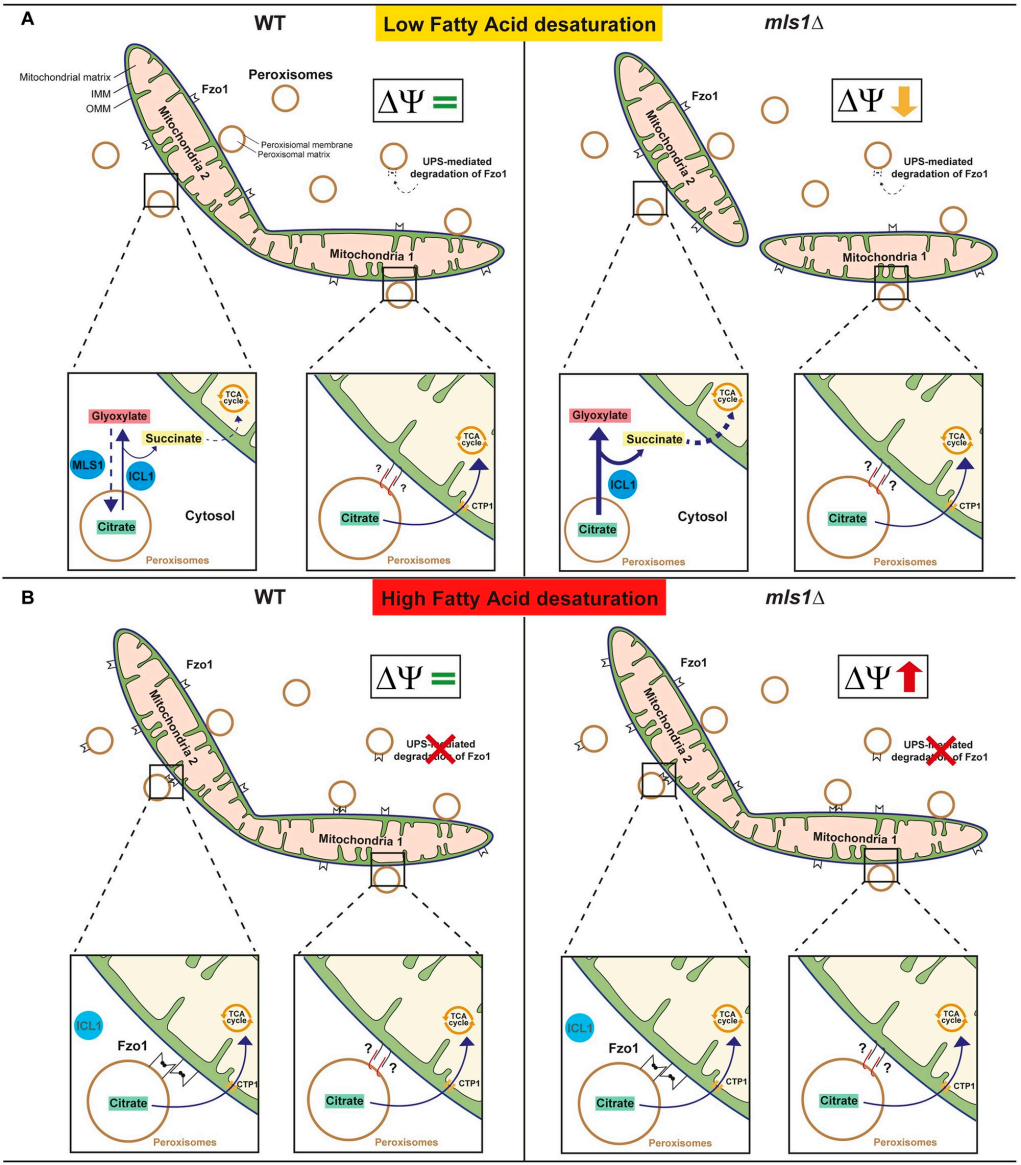
Model of mitochondrial fusion regulation by Fzo1-mediated PerMit contacts. **(A)** Upon low FA desaturation, Fzo1 undergoes increased Mdm30-mediated degradation which limits its accumulation on peroxisomes and precludes formation of Fzo1-mediated PerMit contacts. Other PerMit contacts mediated by distinct factors either identified (Pex34, for instance) or yet to be identified, would promote the transfer of citrate generated by Cit2 to the mitochondrial network thus feeding the TCA cycle and maintaining the mitochondrial membrane potential (*ΔΨ*) required for productive mitochondrial fusion. On the other hand, citrate generated by free peroxisomes would enter the Glyoxylate cycle thereby generating succinate that can transit to mitochondria. Inactivation of MLS1 would result in accumulation of Glyoxylate and Succinate, resulting in excessive import of Succinate, imbalance within the TCA cycle, drop in the *ΔΨ* and inhibition of mitochondrial fusion. **(B)** Upon high FA desaturation, Mdm30-mediated degradation of Fzo1 decreases which promotes accumulation of the mitofusin on peroxisomes and favors the formation of Fzo1-mediated PerMit contacts. This would result in increased transfer of citrate into mitochondria thereby stimulating the *ΔΨ* and counteracting the negative impact that desaturation of FA and phospholipids may have on mitochondrial fusion. Inactivation of *MLS1* under high FA desaturation does not have any effect on mitochondrial fusion as compared to low FA desaturation because of the protection provided by the increase in Fzo1-mediated PerMit contacts that disrupt peroxisomal citrate processing by Icl1 and redirect it toward mitochondria to feed the TCA cycle and stimulate the *ΔΨ*, thereby maintaining efficient mitochondrial fusion. FA, fatty acid; TCA, tricarboxylic acid.

Our observations thus demonstrate that Fzo1 naturally localizes to peroxisomes to associate with mitochondrial Fzo1. This peroxisomal localization of Fzo1 is regulated by Mdm30 and FA desaturation. We find that upon high FA desaturation, Fzo1 accumulates on peroxisomes to facilitate the transfer of peroxisomal citrate to mitochondria where citrate feeds the TCA cycle thereby maintaining the *ΔΨ* and efficient mitochondrial fusion (Fig 8).

## Discussion

Since peroxisomes are an essential hub for FAs metabolism, the potential role of Fzo1 as a PerMit tether [10] echoed our initial discovery that mitochondrial fusion is governed by a balance between Fzo1 degradation and FA desaturation [14]. However, beside a common involvement of Mdm30, the possibility that the 2 pathways could be intricately tied required further investigation. Moreover, numerous fundamental questions remained. How does FA desaturation regulate mitochondrial fusion? Does Fzo1 mediate PerMit contacts in physiological conditions and if so, how is it regulated? Last but not least, what purpose could be served by Fzo1-mediated PerMit contacts?

In the current study, we provide conclusive answers to most, if not all, of these questions. We demonstrate that Fzo1 naturally localizes to peroxisomes (Fig 1) and contributes to about 10% of overall PerMit Contacts in WT cells (Fig 2). The amount of Fzo1 localized at peroxisomes and its propensity to mediate contacts with mitochondria is conditioned by degradation or stabilization of Fzo1 and this rate of Mdm30-mediated turnover is governed by the status of FA desaturation (Fig 3). Our results indicate that peroxisomal Fzo1 associates with mitochondrial Fzo1 to tether peroxisomes to mitochondria (Fig 2E). Fzo1-mediated PerMit contacts allow the transfer of peroxisomal citrate to mitochondria (Figs 5 and 6). This transfer of citrate feeds the TCA cycle to enhance the mitochondrial *ΔΨ* (Fig 6) thereby stimulating efficient mitochondrial fusion as FA desaturation increases (Fig 7). Each of these findings will now be discussed separately.

### The impact of Mdm30 and FA desaturation on Fzo1-mediated PerMit contacts

Similar to its preferred localization at mitochondria, our data infer that the localization of Fzo1 at peroxisomes is natural (Figs 1 and 2). Yet, the amount of Fzo1 at peroxisomes is controlled by the status of FA desaturation and Mdm30-mediated degradation (Fig 3). We have shown previously that upon high FA desaturation, the ubiquitin protease Ubp2 trims the ubiquitin chains added by Mdm30 on Fzo1, which results in stabilization of the mitofusin [14]. This explains the accumulation of Fzo1 at peroxisomes and increased PerMit contacts upon Ole1 overexpression (Fig 3E). Conversely, Mdm30 promotes ubiquitination and degradation of Ubp2 upon low FA desaturation, which results in un-antagonized and increased Mdm30-mediated turnover of Fzo1 [14]. In consequence, peroxisomal localization of Fzo1 decreases upon low expression of Ole1 which results in decreased PerMit contacts (Fig 3E). We confirmed this whole mechanism as this regulation through FA desaturation was abolished upon inactivation of *MDM30* or *UBP2* with increased or decreased PerMit contacts, respectively (Fig 3G and 3H).

### A specific peroxisomal “reset” to investigate the function of Fzo1-mediated PerMit contacts

Inactivation of Fzo1 was efficient to evaluate its contribution to overall PerMit contacts (Fig 2). Yet, this strategy inhibits mitochondrial fusion and induces strong defects in mitochondrial homeostasis precluding analyzing the function of Fzo1-PerMit contacts specifically. Likewise, overexpressing the mitofusin or blocking its degradation to increase Fzo1-PerMit contacts results in an identical perturbation of mitochondrial fusion. In parallel, the finding that Fzo1-mediated PerMit contacts are not constitutive but are formed as FA desaturation increases (Fig 3) imposed distinguishing between the effects of FA desaturation variations from those of Fzo1-mediated PerMit contacts formation. These distinct roadblocks turned out circumvented by the identification of a specific “reset” in peroxisomal function able to induce perturbations of mitochondrial homeostasis unless Fzo1-PerMit contacts were formed. We identified this “reset” as inactivation of the glyoxylate cycle enzyme Mls1 (Figs 4 and 5).

### Fzo1-mediated PerMit contacts and mitochondrial fusion stimulation as FA desaturation increases

In WT cells, we observe that low FA desaturation does not impact mitochondrial dynamics as opposed to high desaturation (Fig 5B), which induces a decrease in mitochondrial fusion (Fig 7A). This results in a significant decrease in tubular mitochondria (Fig 5B, WT cells). The impact of high FA desaturation on mitochondrial fusion is not caused by a decrease in the mitochondrial *ΔΨ* (Fig 6C, WT cells) and is not rescued by exogenous TCA substrates upon deletion of *MLS1* (Fig 7C, *TEF-OLE1*). Whether it could be linked to the remodeling of the phospholipidome upon high FA desaturation [29] will require further investigation in future studies. Nonetheless, this effect easily explains the requirement for Fzo1-mediated PerMit contacts to maintain efficient mitochondrial fusion when FA desaturation increases.

Inhibiting the synthesis of peroxisomal citrate induced a specific decrease in mitochondrial fusion in low but also in high FA desaturation conditions (Fig 7A). This effect upon high FA desaturation is particularly relevant as this is the condition where Fzo1-mediated PerMit contacts are the highest (Fig 3E and S5D Fig) and in which mitochondrial fusion is protected against inactivation of *MLS1* (Fig 7A). This implies that synthesis of peroxisomal citrate in proximity to mitochondria is required to maintain efficient mitochondrial fusion. Consistent with this, inactivation of *CTP1*, a specific mitochondrial transporter of citrate, induced the same effects as inactivating the synthesis of peroxisomal citrate (Fig 7A and S5C Fig). Our data demonstrate that once transported to mitochondria, citrate enters the TCA cycle (Figs 6B, 6D and 7C), thereby maintaining an efficient mitochondrial membrane potential (Fig 6D) which ultimately stimulates mitochondrial fusion to counteract the effects of high FA desaturation (Fig 7C).

### Mitochondrial fusion inhibition under low FA desaturation and inactivation of *MLS1*

While Fzo1-mediated PerMit contacts are rare and may not even occur under low FA desaturation, other PerMit contacts mediated by distinct factors may be able to allow the mitochondrial transport of peroxisomal citrate (Fig 8A), as was demonstrated previously for PerMit contacts mediated by Pex34 [10]. Yet, inactivation of *MLS1* induced strong perturbations on mitochondrial morphology, mitochondrial membrane potential, and mitochondrial fusion (Figs 5B, 6C and 7A). This observation raises the possibility that the transfer of material between peroxisomes and mitochondria is perturbed. In this regard, Icl1 produces Glyoxylate and Succinate from Isocitrate (Fig 5A). Glyoxylate is used by Mls1 to generate Malate while Succinate can be transported to mitochondria [35]. Upon inactivation of *MLS1*, accumulation of Succinate into mitochondria may compete with the transport of citrate, which would induce imbalance in the TCA cycle, decreased *ΔΨ*, and hampered mitochondrial fusion (Fig 8A). In line with this possibility, inactivation of *ICL1* and abrogation of Succinate synthesis totally reversed the effects induced by the absence of Mls1 (Fig 5C and 5D). On the opposite, accumulation of Fzo1-mediated PerMit contacts upon high FA desaturation would bypass Icl1 and thus the accumulation of Succinate, instead increasing the transfer of citrate to stimulate mitochondrial fusion (Fig 8B).

### The role of Acb1 in the regulation of Fzo1-mediated PerMit contacts

Besides *MLS1*, *ACB1* was the other hit of our genetic screen (Fig 4), suggesting that the proteins encoded by both genes are involved in the same pathway. While we do provide conclusive interpretations of the effects linked to *MLS1* inactivation, we can only speculate about the role of Acb1 at this stage. Acb1 is established to bind activated acyl chains, i.e., acyl chains conjugated with Coenzyme A, with very high affinity [33,46]. Acb1 has consequently been proposed to transport activated acyl chains to acyl-CoA consuming processes [31,33]. Among these processes, a role in transport of FAs into peroxisomes would be consistent with a function in peroxisomal citrate synthesis through β-oxidation. Further investigation will be required to clarify this potential role of Acb1 in transport of FAs into peroxisomes. Yet, the established capacity of Acb1 to bind acyl chains already suggests that β-oxidation of FAs is likely a main source of peroxisomal Acetyl-coA for citrate synthesis and stimulation of mitochondrial fusion by Fzo1-mediated PerMit contacts.

### Mitochondrial contacts with other organelles: A conserved function between Fzo1 and Mfn2?

Fzo1 localizes to peroxisomes (Fig 1) and has an apparent capacity to interact with mitochondrial Fzo1 (Fig 2), which results in contacts between peroxisomes and mitochondria. This feature seems conserved in mammalian cells where the human mitofusins Mfn1 and Mfn2 were recently shown to regulate PerMit contacts [11]. Moreover, peroxisomes regulate mitochondrial dynamics in mammalian cells [47]. Intriguingly, Mfn2 fragments also localize to ER membranes [8,48]. Its interaction with mitochondrial Mfn1 and Mfn2 promotes contacts between ER and mitochondrial membranes [8]. These Mfn2-dependent ER–mitochondria contacts have been attributed several functions [49,50]. However, as far as we know, their role in stimulation of mitochondrial fusion has not been investigated. Yet, mitochondrial fusion has been found to be significantly more rapid at sites of contact with the ER (12,5 sec with contact versus 21,9 sec without contact, on average) [51]. The finding that Fzo1-mediated PerMit contacts regulate mitochondrial fusion may thus open novel perspectives regarding the function of ER–mitochondria or PerMit contacts mediated by Mfns and in the physiopathology of the Charcot Marie Tooth type II A disease caused by Mfn2 mutations [52].

## Material and methods

### Yeast strains, growth conditions, and plasmid constructions

The *S*. *cerevisiae* strains and plasmids used in this study are listed in S1 and S2 Tables, respectively. Standard methods were used for growth, transformation, and genetic manipulation of *S*. *cerevisiae* [53]. Complete media or minimal synthetic media [0,67% (w/v) yeast nitrogen base without amino acids (Difco, Voigt Global Distribution, Lawrence, KS), supplemented with 0.1 g/L of each amino acid and nucleic acid base component (Sigma Aldrich), except those used for selection] were supplemented with the following carbon sources: 2% dextrose (SD; YPD for complete media), 2% glycerol (SG; YPG for complete media), 2% Raffinose (SR), or 0,2% Oleic Acid (YPO) (previously dissolved in pure ethanol) supplemented with 1% Tergitol [54,55]. In the indicated strains (see S1 Table), *FZO1*, *PEX3*, *OM45*, *OLE1*, and other genes were chromosomally deleted or C-terminally tagged using conventional homologous recombination approaches [56,57].

The integrative plasmids YIplac128-*MITO-BFP* and YIplac128-*mRFP-SKL* were constructed as follows: the mt-BFP sequence was amplified by PCR from the *pRS426-ADH1-mtBFP* plasmid (MC459) [58] and the mRFP-SKL sequence was amplified from pRS316-mRFP-SKL (MC374) [59]. The resulting products were inserted into the YIplac128 plasmid (Leu2/INT) digested with *Kpn*I or into the YIplac128-*MITO-GFP* (MC363) [60] digested with XhoI and NcoI with the In-Fusion HD Cloning Kit (Clontech, 638909) to yield YIplac128-mRFP-SKL (MC547) and YIplac128-*MITO-BFP* (MC460), respectively. These 2 plasmids were sequenced before use and then digested with *Eco*RV (restriction site in *LEU2*) and yeast cells were transformed with 0.5 μg of digested DNA to generate strains in which *RFP-SKL* or *MITO-BFP* was integrated into the *LEU2* locus.

The p*RS414-OLE1* plasmids and p*RS416-OLE1-9MYC* plasmids were constructed as follows: the *OLE1* and *OLE-9Myc* coding sequences were amplified by PCR from the genomic DNA of strain WT W303 (*MCY553*) or W303 *OLE1-9Myc* (*MCY1043*), respectively. The resulting products were inserted into the pRS416 (*URA3*, CEN) plasmid or into the pRS414 (*TRP1*, CEN) plasmid containing different promoters (CYC, TEF, ADH, MET25) [61] digested with the *EcoRI* and *ClaI* enzymes using the In-Fusion HD Cloning Kit (Clontech, 638909) to yield p*RS416-OLE1-9MYC*, p*RS414-OLE1-9MYC*, and p*RS414-OLE1* plasmids with different promoters. The *pRS414-prom Ole1-OLE1* plasmid was obtained by PCR amplification of the coding sequence of Ole1 flanked by 0.5 kb of the promoter region and 0.5 kb of the terminator of strain WT W303 (*MCY553*). The resulting product was inserted into an empty pRS414 (*TRP1*, CEN) plasmid (MC383) [62], after digestion by *SmaI*, using the In-Fusion HD Cloning Kit (Clontech, 638909) to yield p*RS414-prom Ole1-OLE1* (MC543).

### Generation of *FZO1*, *MDM30*, and *OLE1* shuffle strains

*FZO1* and *MDM30* null cells tend to lose their mitochondrial DNA because of decreased mitochondrial fusion efficiency [30]. A plasmid-shuffle strategy was thus employed to ensure a reliable genetic analysis of *mdm30Δ* and *fzo1Δ* cells as described in [14]. Unlike *FZO1* and *MDM30*, the *OLE1* gene is essential in *S*. *cerevisiae* [16]. Consequently, wild-type W303 cells were transformed with *pRS416-OLE1-9MYC* (*OLE1* shuffle plasmid) prior to the chromosomal deletion of *OLE1* to generate *OLE1* shuffle strains and keep the cells alive. *OLE1* inactivation was verified by replica-plating *OLE1* shuffle strains on 5′-fluoroorotic acid (5-FOA) plates in the absence of an additional plasmid expressing *OLE1*. Loss of the uracil *OLE1* shuffle plasmid resulted in 100% lethality which confirmed the absence of *OLE1*.

To yield strains used in this study, *FZO1*, *MDM30*, and *OLE1* shuffle strains were then transformed with plasmids of interest listed in S2 Table. Ten colonies were systematically isolated on SD selective media lacking uracil and replica-plated on corresponding SG and 5-FOA plates. Strains grown on 5-FOA plates and cured from shuffling plasmids were in turn replica-plated on SD and SG selective plates containing uracil. The glycerol growth phenotypes of strains covered by or cured from the shuffling plasmids were reproducibly observed in 100% of clones tested after 1 to 3 days of growth at 30 °C. Representative colonies were used in subsequent experiments.

### Quantification of PerMit contacts

Peroxisome-mitochondria (PerMit) contact sites were determined by sorting peroxisomes into 2 categories: those Attached and those Non-Attached (Figs 1A, 2C and 3D). Using Structured Illumination microscopy (see SIM section below), we reached a resolution range of about 120 nm. We quantified the distance between attached peroxisomes and mitochondria using FWHM (full width at half maximum). A line across peroxisomes and mitochondria was drawn to measure the distance between the curve points at the peak half maximum level. We analyzed more than 100-line profiles which included peroxisomes both in contact and in close proximity with mitochondria. The distance for peroxisomes that were not in contact with mitochondria ranged from 300 to 180 nm. In contrast, the distance for peroxisomes that appeared in contact with mitochondria ranged between 120 to less than 20 nm. Notably, we frequently observed a complete overlap between the intensity profile of peroxisomes and mitochondria. This means that the actual distance between the corresponding membranes is far below the measured mean intensities and thus falls below the 80 nm top limit which has been usually suggested for other contacts [63]. These contacts sites also verify the proposed remaining characteristics of a membrane contact site [63]: Fusion events between peroxisomes and mitochondria were not observed, a distinct set of proteins, in these case Fzo1, is described as tethering the 2 organelles (Shai and colleagues and this study) and finally, these contacts serve a specific function such as metabolites transfer (this study) which allows to safely assume formation of bona fide PerMit contact site.

### Transmission electron microscopy and immunolabeling

*FZO1-GFP mdm30Δ* cells (MCY1417) grown in Dextrose-containing complete media (YPD) and *FZO1-GFP WT* cells (MCY1415) grown in Oleate-containing complete media (YPO) were lightly chemically fixed with 4% of paraformaldehyde for 1 h at room temperature followed by overnight incubation at 4 °C. Fixed samples were resuspended in PBS 1× with 10% gelatin (Sigma G2500) and cryoprotected in 2.3 M sucrose at 4 °C before freezing in liquid nitrogen. Cells were sectioned by cryo-ultramicrotomy (60 nm thickness) using an UC6 ultramicrotome Leica and retrieved cryo-sections were thawed. Thin sections were then immunolabeled with anti-GFP diluted at 1:100 and PAG 10 nm. Sections were stained and imaged using a transmission electron microscope Technai TEM 120kV equipped with a 4k × 4k Eagle camera Thermo Fisher.

### Protein extracts and immunoblotting

The 1 OD_600nm_ of cells (1 ml) grown in SD media were collected during the growth exponential phase (OD_600nm_ = 0.5–1) and total protein extracts were prepared using the NaOH/trichloroacetic acid lysis method [64]. Briefly, lysis was performed with 10% (vol/vol) of 1 M NaOH on ice for 10 min before protein precipitation with 5% (final) trichloroacetic acid (TCA) on ice for 10 min. Proteins were then pelleted and resuspended in Sample Buffer (13.33 mM Tris-HCL (pH 6.8), 1.6 mM EDTA, 1.6% SDS, 40 mM DTT, 8% Glycerol, 0.016% Bromophenol Blue, 333 mM Tris-Base) and heated at 70 °C for 10 min. Proteins were separated on SDS-PAGE (8% or 10% Acrylamide) and transferred onto nitrocellulose membranes (Amersham Hybond-ECL; GE Healthcare). The primary antibodies used for immunoblotting were: monoclonal anti-Pgk1 (1/20,000, AbCam, ab113687), monoclonal anti-HA (1/1,000, 12CA5, Invitrogen, 71–5500), monoclonal anti-Por1 (1/1,000, AbCam, ab110326), monoclonal anti-Myc (1/1,000, 9E10, Invitrogen, R950-25), and polyclonal anti-Fzo1 (1/1,000, generated by Covalab). Primary antibodies were detected by secondary anti-mouse or anti-rabbit antibodies conjugated to horseradish peroxidase (HRP, 1/5,000, Sigma-Aldrich, 12–348 and A5278)—or anti-mouse IgG for IP (ab131368) and Goat anti-Mouse by Jackson ImmunoResearch (115-035-003) for IPs in Fig 1D—followed by incubation with the Clarity Western ECL Substrate (Bio-Rad). Immunoblotting images were acquired with a Gel Doc XR+ (Bio-Rad) before treatment and quantification with the Image Lab 6.0 software (Bio-Rad). The cytosolic protein PGK1 was used as a loading control to normalize loading of other proteins relative to the WT conditions. Data reported are the mean and SEM (error bars) of 3 independent experiments.

### Cellular fractionation assay

Cells were grown overnight in SD media to exponential phase (OD_600nm_ = 0.5–1) and 500 ODs were collected by centrifugation at 1,500×*g* for 5 min before resuspension in 0.1 M Tris-HCl pH = 9.4–50 mM β-mercapto-ethanol (1 ml per 20 ODs of cells) and incubated for 1 h at 30 °C to weaken the cell wall [65,66]. Following centrifugation, cells were washed with 1.2 M sorbitol and resuspended in 1.2 M sorbitol (1 ml per 50 ODs) supplemented with Zymolyase (Zymo Research; Orange, CA) (100U/50 OD) to induce cell wall digestion and generate spheroplasts. After a 1 h 30 incubation at 30 °C, spheroplasts were collected at 1,500 g and washed with 1.2 M sorbitol to finally be resuspended in ice-cold Homogenization Buffer [10 mM Tris-HCl (pH 7.4), 0.6 M sorbitol, 1 mM EDTA, 0.2% (w/v) BSA + protease inhibitor pills (1 pill in 50 ml buffer; Protease Inhibitor Cocktail; Sigma-Aldrich) and 1 mM Pefabloc (Sigma-Aldrich)]. Spheroplasts were disrupted by douncing on ice (100 times with a medium size glass dounce of 15 ml) and the resulting homogenate cleared by centrifugation at 3,000×*g* for 5 min. The cleared homogenate (total fraction) was then subjected to centrifugation at 10,170×*g* for 10 min, yielding a soluble fraction in the supernatant (Sup) and a mitochondrial-enriched membrane fraction (Pellet). The soluble fraction was subsequently subjected to an additional centrifugation at 10,170×*g* for 5 min to clear the supernatant from any residual mitochondrial contamination. Aliquots of each fraction were collected proteins were and precipitated with addition of 5% (final) TCA and incubation on ice for 30 min prior to resuspension in sample buffer, boiling, electrophoresis and immunoblotting.

### Structured illumination microscopy (SIM)

SIM was used to quantify PerMit contacts in vivo, where indicated. Strains were grown in dextrose-containing medium at 30 °C to mid-log phase and 1 ml of culture was centrifuged at 1,500g for 3 min in a 1.5 ml Eppendorf tube. After resuspension of cell pellets into 50 μl of YPD media, 5 μl of cell suspension was loaded on 25 mm coverslip (CG15XH, THORLABS) and placed in a magnetic chamber (#CM-B25-1, Quorum Technologies). Yeast cells were immobilized using a YPD pad placed over the cell suspension, which helps to spread the cells homogenously into a single layer suitable for imaging. Super-Resolution (SR) images of yeast cells were acquired using an SIM Zeiss LSM 780 Elyra microscope (Carl Zeiss, Germany) controlled by the Zen software. The microscope was equipped with an oil immersion 100× Plan-Apochromat objective with a 1.46 numerical aperture and an additional 1.6× lens. For detection, an EMCCD Andor Ixon 887 1K camera was used. One SR-SIM image was reconstructed from 9 images acquired from 3 different phases and 3 different angles. Acquisition parameters were adapted to optimize the signal to noise ratio according to yeast strains. SIM images were processed with ZEN software and then channel alignment was performed using 100 nm TetraSpeck fluorescent beads (T7279, Invitrogen) embedded in the same conditions as the sample. SIM images were analyzed and quantified using ImageJ (National Institute of Health) open-source software.

### Confocal microscopy

Confocal microscopy was used to quantify PerMit contacts in vivo, where indicated. Confocal cross-section images were acquired using a Spinning-disk UltraView VOX (Perkin Elmer) system equipped with a confocal scanning head (CSU X1; Yokogawa), a 100× 1.4 NA oil immersion objective, and EMCCD cameras (ImageEM C9100; Hamamatsu Photonics) controlled by Volocity software. The GFP and mCherry channel images were acquired using 488 and 561 lasers with 20% and 15% of laser intensity, respectively, and a maximum of 300 ms of exposure time. Image post-processing was performed using ImageJ (National Institute of Health) open-source software.

### Conventional fluorescence microscopy

Fluorescence microscopy was carried out with a Zeiss Axio Observer.Z1 microscope (Carl Zeiss S.A.S.) with a ×63 oil immersion objective equipped with the following filter sets: FITC (Filter set 10-Alexa 489, Excitation BP 450/490, Beam Splitter FT 510, Emission BP 515–565) for GFP, HC (Filter set F36-508 Chroma, Excitation BP 562–40, Beam Splitter HC-BS 593, Emission BP 641/75) for RFP, and DAPI (Excitation BP 365–12, Beam Splitter FT 395, Emission LP 397-∞) for BFP. Cell contours were visualized with DIC (differential interference contrast) optics. Images were acquired with an ORCA-Flash 4.0 charge-coupled device camera (Hamamatsu). Images were treated and analyzed with ZEN 3.1 (Blue edition) and ImageJ (National Institute of Health).

### In vitro imaging of PerMit contacts

Cells with integrated mito-BFP in which Fzo1 and/or Pex3 are genomically tagged with GFP or mCherry at their C-terminus were grown to exponential phase and subjected to cellular fractionation assays (see above). The isolated mitochondrial fractions were fixed with 2 volumes of 8% formaldehyde in PBS 1× for at least 20 min at room temperature and then stained with 1× MitoLite blue FX490 (AAT-Bioquest) for 30 min at 4 °C to increase mitochondrial staining in blue. Cells were washed and resuspended in PBS 1×. Cover slips were coated beforehand by incubation for 5 min in 100 μl poly-D-Lysine at 0.1% (w/v in water). After removal of excess poly-D-Lysine by suction, coated coverslips were left to dry for 90 min. Following washing of the coverslips twice with PBS 1×, 100 μl of the final mitochondrial suspension was placed on the slips for 5 min then washed again with PBS to remove excess membranes. Imaging of PerMit contacts in the mitochondria-enriched fractions was achieved by conventional fluorescence microscopy. Contacts between peroxisomes and mitochondria were determined by the proximity of the red signal (Pex3-mCherry) with the green (Fzo1-GFP) and blue signal (mitolite and mito-BFP) which indicated a likely attachment between the 2 organelles. Mitochondrial aggregates that were labeled with FZO1-GFP and mt-BFP sometime looked blurry because image acquisition was essentially performed focusing on peroxisomes labeled with mCherry. Data reported are the mean and SEM (error bars) of 3 independent experiments.

### Native immunoprecipitation of peroxisomes

Cells grown to exponential phase were subjected to fractionation as described above. Aliquots of Total, Pellet, and Supernatants (pre-IP lysate) fractions were diluted and heated in sample buffer for subsequent immunoblotting. The remaining supernatant was collected after the 2 centrifugations at 10,170×*g* while the membrane pellet enriched in mitochondria was discarded. The supernatant was then diluted 2-fold in homogenization buffer supplemented with protease inhibitor but without any detergent. Each supernatant half was incubated overnight on spinning wheel (12 rpm) at 4 °C with ROCHE Anti-HA Affinity matrix (Sigma 11815016001). The next day, the beads were washed 3 times with detergent-free homogenization buffer and heated in sample buffer before resolution by SDS-PAGE and analysis by immunoblotting as described above and with indicated antibodies.

### RFP-Trap peroxisome immunoprecipitation assay

In this assay, strains in which Fzo1 and/or Pex3 are C-terminally tagged at the genome with GFP and mCherry, respectively, were employed (*MCY1667*, *MCY1580*, *MCY1675*, *MCY1673*, *MCY1551*, *MCY1677*); 200 to 250 ODs of cells grown at exponential phase were first subjected to fractionation as described above. The resulting supernatant was diluted 2-fold (25 OD/ml) in homogenization buffer supplemented with protease inhibitors but without detergent and processed for native IP of peroxisomes. The diluted supernatant split in 2 halves was incubated overnight with (IP+) RFP-Trap Magnetic Agarose beads (rtma, ChromoTek) or with (IP-) binding control magnetic Agarose beads (bmab, ChromoTek) at a concentration of 1 μl of beads/5 OD cells. The next day, the beads were collected using a magnetic rack (GE Magrack 6) and washed 3 times for 5 min each with homogenization buffer (with or without 0.6% TritonX100 as indicated) on spinning wheel at 4 °C. The beads were subsequently fixed with 3.7% formaldehyde for at least 20 min at room temperature, followed by imaging in fluorescence mounting medium (Dako) and analysis by conventional fluorescence microscopy. Specific Fzo1-GFP signal was first analyzed by determining the percentage of beads with peroxisomes (red fluorescence) which displayed green fluorescence (Figs 1F and 2B, left graph). In parallel, the average green signal intensity per bead was determined by quantifying green fluorescence intensity on the surface of the bead divided by the total number of beads analyzed. To extract fluorescence intensity data, a dedicated macro script was written in ImageJ. First, the background fluorescence from each fluorescent channel was determined by creating an ROI (region of interest) outside the area occupied by the beads for which we measured the mean intensity. This mean background intensity was then subtracted from the entire field of view. In turn, bead segmentation was performed on the transmission channel after smoothening the image twice and applying the minimum filter to blur (low intensity in 7 pixel radius) followed by the Gaussian blur filter (sigma value 5). In addition, an automatic default threshold was applied, and the image was converted into a binary mask. A fill hole and watershed binary operation was performed to separate beads touching each other. Finally, the analyze particle plugin with size (10,000 to 20,000 pixels) and circularity (0.7 to 1) filter was used to quantify beads. Using the ROI manager plugin, each bead location was added to the ROI manager, and the fluorescence intensity was measured in that region in each background-free image. The green fluorescence intensities obtained were in turn normalized to the intensity value of the *mdm30Δ* mutant (Fig 1G) or the S201N mutant (Fig 2B, right graph). An average of 155 beads was analyzed for the WT/Mdm30D experiment (add fig number) and 128 beads for the WT/S201N experiment (add figure number). Data reported are the mean and SEM (error bars) from 3 independent experiments.

### Peroxisome/mitochondria ex vivo mixing assays

Peroxisome immunoprecipitation using RFP Traps (see above) was the first step of the mixing assays. Peroxisomes from *mdm30Δ* strains in which Pex3 is either un-tagged (*MCY1842*) or C-terminally tagged at the genome with a mCherry epitope (*MCY1551*) were pulled down using RFP-Trap magnetic beads. The next day, after washing with homogenization buffer, the beads were incubated with purified mt-GFP tagged mitochondria that were prepared the same day. Mitochondria labeled with mt-GFP were purified from WT (*MCY1843*) or *pex34Δ* (*MCY1978*) cells which express either a WT copy of *FZO1* (*MC250*) or the GTPase domain mutant (*S201N*) of Fzo1 (*MC544*). These cells grown at exponential phase were subjected to fractionation as described above, yet, in this case, the supernatant was discarded and the membrane pellet enriched in mitochondria was resuspended in homogenization buffer (1 OD/μl). The mitochondrial pellet was then split in 2 halves that were further diluted 2-fold in homogenization buffer before incubation with each set of beads overnight at 4 °C on spinning wheel (12 rpm). On the following day and after 3 consecutive washing cycles with homogenization buffer on spinning wheel for 5 min, the beads were imaged by conventional fluorescence microscopy. Mitochondrial attachment to RFP (RFP+) beads and control (RFP−) beads appeared as clear and distinctive puncta and was assessed in at least 100 beads by conventional fluorescence microscopy. Specific mitochondrial attachment indicates the number of green mitochondria attached to the beads divided by the total number of beads counted. The percentage of attachment to the control RFP− beads was subtracted from the percentage of attachment to RFP+ beads resulting in the specific attachment percentage reported in the figure (Fig 2E). Data reported are the mean and SEM (error bars) from 3 independent experiments.

### Spot assays

Cultures grown overnight in SD medium were pelleted, resuspended at OD_600_ = 1, and serially diluted (1:10) 5 times in water. Three microliters of the dilutions were spotted on Dextrose, Glycerol, Oleic Acid plates (with the appropriate amino acid selection), and grown for 2 to 4 days (Dextrose) or 3 to 6 days (Glycerol and Oleic Acid) at 23, 30, or 37 °C.

### High-throughput genetic screen

The rescue of *mdm30Δ* cells by the extra copy of *FZO1* prompted us to perform a genetic screen to search for gene deletions that could abolish this rescue. This screen was carried through synthetic genetic array (SGA) techniques [67] using the *Mat alpha SGA* ready strain *yMS721* and a *Mat a G418* selection yeast deletion library [68] that are built in the *BY* genetic yeast background. In this background, the respiratory growth defect of *mdm30Δ* cells is only detected at 37 °C on glycerol media [30]. After confirming the rescue by the *FZO1* extra copy is also seen in this background and at this temperature, *yMS721* was transformed by the extra *FZO1* copy plasmid (MC250) to yield the *MCY1513* strain (S1 Table). *MDM30* was subsequently deleted in *MCY1513* to generate the *MCY1528* strain. *WT* (*MCY1513*) and *mdm30Δ* (*MCY1528*) strains were mated with the *Mat a* yeast deletion library [68]. The zygotes obtained after mating were sporulated and, after selection, the resulting haploid strains containing both the extra *FZO1* plasmid as well as the deletion of *MDM30* and the deletion of the library were grown on glycerol 1536 plate format for 7 days at 37 °C. To manipulate libraries in 1536-colony high-density format, a RoToR bench top colony arrayer (Singer Instruments) was used. In this primary screen, the positive hits were defined as candidates that present a growth defect in the *mdm30Δ* context but that are unaffected in the *WT* control. Taking advantage of the identical disposition of deletion mutants on *WT* and *mdm30Δ* plates, the screening of the hits was performed using a colorization approach. Briefly, *WT* colonies were colored in red while *mdm30Δ* colonies were colored in green. Superimposition of *WT* red plates over *mdm30Δ* green plates resulted in a vast majority of yellow colonies. The potential hits corresponded to red colonies with significantly reduced green coloration reflecting a growth defect of *mdm30Δ* as compared to *WT*. This approach yielded a dozen of potential hits that were subsequently verified in a secondary screen carried out in the *W303* background.

In this secondary screen, deletions of candidate genes identified in the primary screen were introduced in the *mdm30Δ* shuffle strain MCY970. The resulting strains were transformed with the *FZO1* extra copy plasmid (*MC250*) or by an empty vector (*MC219*) before curation of the *MDM30* shuffle plasmid on 5-FOA media. The resulting *MDM30* negative and *MDM30* positive strains were processed for spot assays on Dextrose, Glycerol, and Oleate media at 23, 30, and 37 °C, respectively. This secondary screen yielded only 2 high confidence hits. Inactivation of *ACB1* or *MLS1* induced significant inhibition of the respiratory rescue of *mdm30Δ* cells by the extra copy of *FZO1* but had more limited effects on *MDM30* positive cells.

### Mitochondrial network morphology

Mitochondrial morphology was scored in cells genomically expressing OM45-GFP or mt-GFP as indicated. Strains were grown in dextrose containing medium at 30 °C (unless indicated otherwise) to mid-log phase and fixed with 3.7% formaldehyde for at least 20 min at room temperature. Morphology phenotypes were assessed in at least 100 cells by conventional fluorescence microscopy. Data reported are the mean and SEM (error bars) from 3 independent experiments.

### Assessing preCox4-mCherry import to mitochondria

Import of nuclear-encoded proteins into the mitochondrial matrix requires a normal mitochondrial membrane potential (ΔΨ) [69]. Thus, we used the chimeric fusion protein preCox4-mCherry that includes the inner mitochondrial membrane targeting pre-sequence of Cox4 fused to mCherry as a readout to monitor the mitochondrial membrane potential in vivo [43]. In our experiments, we designed the strain *MCY2080* carrying OM45-GFP as a control mitochondrial marker and pre-COX4-mCherry which was integrated at an intergenic region near the centromere of chromosome IV. While pre-COX4-mCherry requires the ΔΨ for its import into mitochondria, OM45 is an outer membrane protein for which import is not affected by changes in the ΔΨ [70]. Prior to imaging, cells were grown to mid-log phase at 30 °C in SD medium and supplemented when required with 10 mM citrate (citric acid—Merck, C2404), 10 mM alpha-ketoglutarate (alpha-ketoglutaric acid—Merck,75890), or 10 mM glutamate (L-glutamic acid—Merck, G5889). The pH of the media was adjusted using few drops of KOH 10 M solution upon addition of citrate or alpha-ketoglutarate. Cells were subsequently fixed with 3.7% formaldehyde for at least 20 min at room temperature. Using conventional fluorescent microscopy, we counted cells which exhibited a diffuse cytoplasmic localization of preCox4-mCherry, reflecting defects in pre-COX4-mCherry import and in turn a drop in the ΔΨ, while the mitochondrial localization of OM45-GFP was unaffected. The localization of preCox4-mCherry was assessed in at least 100 cells. Data reported are the mean and SEM (error bars) from 3 independent experiments.

### Time lapse fluorescence imaging and quantification of fusion and fission events

Strains were grown in dextrose-containing medium at 30 °C to mid-log phase, and samples were prepared as described above in the SIM or Confocal microscopy sections of the Material and methods. Time-lapse images were acquired for 3 to 5 min with 5 to 10 s time interval depending on the experiments. The live analysis by SIM achieves a resolution of 120 nm that resolves fusion and fission events within tubular, fragmented but also aggregated mitochondrial networks (see S6A Fig). The number of cells with at least 1 fusion or fission event over the 3 to 5 min acquisition timeframe of the movies was quantified to yield the “% of cells with fusion event” or the “% of cells with fission event.” Using this approach on restricted periods as compared to the yeast lifespan, cells with more than 2 fusion or fission events were never observed. More precisely, mitochondrial networks within the vast majority of cells (86% to 96%) did not show any fusion or fission event. Only 1% of cells had either 2 fusion or 2 fission events, whereas 6% to 15% of cells had at least 1 fusion or fission event. In the graphs from Fig 7 depicting the % of cells with fusion or fission events, the possible 1% of cells with 2 fusion or fission events is embedded in the overall % of cells with fusion or fission events. Multiple videos were analyzed yielding a total of at least 100 cells in each experiment. All data reported are the mean and SEM (error bars) of 3 independent experiments.

### In vivo mitochondrial fusion assays

Mitochondrial fusion in zygotes was examined essentially as described [71]. Indicated MATa and MATα cells (see S1 Table) were respectively transformed with pYeL1-mtGFP and pYeL1-mtRFP that allow expression of mito-GFP or mito-RFP under control of the GAL10 promoter. For each fusion assay, haploid cells of opposing mating types were grown overnight in SR media complemented with 2% Galactose. Next morning, expression of mtGFP and mtRFP were repressed for 3 h by addition of 2% Glucose in cultures. MATa and MATα cells were then mixed in SD media and mated during 4 h before analysis by fluorescence microscopy. Mitochondrial fusion was quantified in 25 large-budded zygotes per strain in 3 separate experiments. Data reported are the mean and SEM of all experiments.

## Supporting information

S1 Fig **(A)** Dextrose (Glucose) and Glycerol spot assays at 23, 30 and 37 °C of *WT* (*MCY1589*) and *FZO1-GFP* tagged strains (*MCY1671*) genomically labeled for *mito-BFP* and the corresponding percentage of cells with tubular mitochondria at 30 °C. Note that the GFP C-terminal tagging does not affect the function of Fzo1. **(B)** Transmission electron microscopy micrographs from *mdm30Δ* cells grown in Dextrose-containing media and WT cells grown in Oleate-containing media. Green arrowheads indicate mitochondria. Red arrowheads indicate circular structures with clear lumen that correspond to peroxisomes. Orange squares on left micrographs highlight the regions zoomed in on right images. Scale bars correspond to 500 nm. Note that peroxisomes proliferate and are detected in Oleate-containing media but not in Dextrose-containing media. **(C, D, and E)** Micrographs showing Fzo1-GFP immuno-staining on mitochondria (C) and peroxisomes (D and E) from WT cells grown in Oleate-containing media. Scale bars correspond to 200 nm. Note that Fzo1-GFP immuno-staining appears as the black dots seen on mitochondria labeled in green or peroxisomes labeled in red. Underlying data for quantifications can be found in S1 Data.
(TIF)

S2 Fig **(A)** Schematic representation of native Immuno-Precipitation of peroxisomes from *WT* and *mdm30Δ* cells genomically labeled for *PEX3-mCherry*, *FZO1-GFP*, and *mt-BFP* (*MCY1667*, *MCY1591*, *MCY1675* and *MCY1673*, *MCY1597*, *MCY1677* cured from the *MDM30* shuffle plasmid). Cells were processed for fractionation assays to yield whole cell (Total), cytosol (Sup), and membrane (Pellet) fractions. Cytosol fractions were then split in 2 halves and incubated O.N. with mock (−) or RFP (+) Trap beads in the absence of detergent to pull-down Pex3-mCherry native peroxisomes specifically. After washing, beads were analyzed with DIC or fluorescence microscopy for detection of Fzo1 (GFP), Pex3 (RFP), and mitochondria (BFP). **(B)** DIC and fluorescence microscopy analysis of mock beads after washing (see also Fig 1E for RFP Trap beads). Scale bars correspond to 10 μm. Note that GFP (green), RFP (red), or BFP (gray) signals are not detected, indicating that peroxisomes (Pex3-mCherry), mitochondria (mt-BFP), or Fzo1-GFP do not bind nonspecifically to the beads. **(C)** Same experiment as in Fig 1F but washing of RFP Trap beads was performed either in the absence (NT) or in the presence (T) of Triton detergent. The graph shows the percentage of beads with BFP (blue), mCherry (red), or GFP (green) signal in each condition. Note that Fzo1-GFP is detected in the NT condition, in the absence of BFP signal and thus in the absence of mitochondria, but that this Fzo1-GFP is lost in the presence of detergent. This confirms that Fzo1 is embedded in peroxisomes from *WT* and *mdm30Δ* cells. **(D)** Percentage of peroxisomal attachment (mCherry signals) to mitochondria (GFP/BFP signals) in Mitochondrial Enriched Fractions from *WT* (blue bars) and *FZO1-S201N* (red bars) cells labeled for both *PEX3-mCherry*, mito-BFP, and *FZO1-GFP* (*MCY1771* and *MCY1772*). Error bars represent the SEM from 3 independent experiments. * *P* = 0.05 (one-way analysis of variance (ANOVA)). Underlying data for quantifications can be found in S1 Data.
(TIF)

S3 Fig **(A)** Schematic representation of ex vivo PerMit contact assays (Panel I). DIC and fluorescence microscopy analysis of RFP Trap beads after overnight incubation with the cytosolic fractions of *mdm30Δ mito-GFP* cells either genomically labeled or unlabeled for *PEX3-mCherry* (*MCY1842* and *MCY1847* cured from the *MDM30* shuffle plasmid). Note the negative signal for GFP, confirming that mitochondria are absent from cytosolic fractions and do not pull-down with peroxisomes. **(B)** Ex vivo PerMit contact assays (Panel III). DIC and fluorescence microscopy analysis of untagged peroxisomes RFP Trap beads from S1A (bottom row) after overnight incubation with the membrane fractions of *WT FZO1* and *FZO1 S201N* cells genomically labeled for *mito-GFP* (*MCY1843* transformed with *pRS314-FZO1* (*MC250*) or *pRS314-FZO1-S201N* (*MC544*)). Note the positive signal for GFP that reflects the nonspecific binding of mitochondria to RFP Traps and that was quantified and subtracted from the specific binding shown in Fig 2E. **(C, D, and E)** Preparation and characterization of *OLE1* shuffle strains. **(C)** *ole1Δ* cells covered by a *pOLE1-9MYC* shuffle plasmid with *URA3* selection were transformed with an empty vector or with *pOLE1* plasmids under control of *OLE1*, *CYC*, or *TEF* promoters with *TRP1* selection. Resulting double transformants were patched on Synthetic Dextrose media without Uracil and Tryptophan (SD -U-T) and replica-plated on Synthetic Glycerol media without Uracil and Tryptophan (SG -U-T) or on 5-FOA media without Tryptophan (5-FOA -TRP) to initiate curation of the *pOLE1-9MYC* shuffle plasmid with *URA3* selection. Note that after a second replicate on 5-FOA media without Tryptophan, the growth of the empty vector strain was abolished as expected since *OLE1* is essential for viability. **(D)** Whole cell extracts of *ole1Δ* strains shuffled with *MET25*, *CYC*, *ADH*, or *TEF pOLE1-9MYC* plasmids (*MCY1798*, *MCY1797*, *MCY1796*, *MCY1795*) were processed for western blotting with anti-Myc, anti-PGK, and anti-Porin. Molecular weights in kDa are indicated on the right. Note that the stronger the promoter (*TEF* > *ADH* > *MET25* > *CYC*), the highest the expression of Ole1-9Myc. **(E)** Dextrose, Glycerol, and Oleate spot assays at 23, 30, and 37 °C of *ole1Δ* strains (*MCY1781*) shuffled with *OLE1*, *CYC*, or *TEF pOLE1* plasmids (*MC540*, *MC541*, *MC543*). Besides on Dextrose at 23 °C, the relative growth of *OLE1*, *CYC-OLE1*, or *TEF-OLE1* cells is not significantly different. **(F)** Average number of Peroxisomes per cell in *CYC-OLE1* (yellow bars), *OLE1* (blue bars), and *TEF-OLE1* (red bars) cells from Fig 3D. Error bars represent the SEM from 3 independent experiments. NS, not significant. Note that regardless of Ole1 expression, the average number of peroxisomes per cell is equivalent and does not depend on FA desaturation. Importantly, some cells such as those seen in Fig 3D showed 4 to 6 peroxisomes while others had 2, 1, or no peroxisomes that could be detected, resulting in an average of 2 in all conditions. Underlying data for quantifications can be found in S1 Data. Uncropped scans are depicted in S1 Raw Images.
(TIF)

S4 Fig **(A)** Dextrose, Glycerol, and Oleate spot assays at 30 °C of *ole1Δ mdm30Δ* strains (*MCY1959*) transformed with *pRS314-FZO1* (*MC250*) or an empty vector (*MC219*) and shuffled with *OLE1*, *CYC*, or *TEF pOLE1* plasmids *(MC540*, *MC541*, *MC543*). Note that the extra copy of *FZO1* not only rescues the growth of *mdm30Δ* cells on glycerol but also on Oleate, suggesting a stimulation of peroxisomal function. **(B)** Examples of false positive hits of the primary screen that were characterized in the secondary screen. Dextrose and glycerol spot assays at 30 °C of *MDM30* (*MCY970*), *MDM30 atp12Δ* (*MCY1616*), and *MDM30 erv29Δ* (*MCY1610*) shuffling strains transformed with *pRS314-FZO1* (*MC250*) or an empty vector (*MC219*) and covered by (*mdm30Δ + MDM30*) or cured from (*mdm30Δ*) the *MDM30* shuffle plasmid. Absence of *ATP12* (a factor required for assembly of the ATP synthase) abolishes the respiratory rescue of *mdm30Δ* cells by *FZO1* but also blocks the respiratory growth of *MDM30* positive cells. Absence of *ERV29* (a factor involved in COPII vesicles formation) does not affect the respiratory rescue of *mdm30Δ* cells by *FZO1*. **(C)** Confirmed candidates after the secondary screen. Dextrose, Glycerol, and Oleate spot assays at 23, 30 and 37 °C of *MDM30* (*MCY970*), *MDM30 acb1Δ* (*MCY1612*), and *MDM30 mls1Δ* (*MCY1649*) shuffling strains transformed with *pRS314-FZO1* (*MC250*) or an empty vector (*MC219*) and covered by (*mdm30Δ + MDM30*) or cured from (*mdm30Δ*) the *MDM30* shuffle plasmid. Note that the absence of *ACB1* affects the respiratory rescue of *mdm30Δ* cells by *FZO1* at higher temperatures (30 and 37 °C), whereas the absence of *MLS1* does so at lower temperatures (23 and 30 °C). Interestingly, the absence of *ACB1* also affects the growth rescue of *mdm30Δ* cells by *FZO1* on Oleate at 23 and 30 °C. As expected, absence of *MLS1* abolishes the growth of all cells on Oleate media. **(D)** Dextrose, Glycerol, and Oleate spot assays at 23, 30 and 37 °C of *MDM30* (*MCY970*), *MDM30 mls1Δ* (*MCY1649*), *MDM30 icl1Δ* (*MCY1909*), and *MDM30 mls1Δ icl1Δ* (*MCY1911*) shuffling strains transformed with *pRS314-FZO1* (*MC250*) or an empty vector (*MC219*) and covered by (*mdm30Δ + MDM30*) or cured from (*mdm30Δ*) the *MDM30* shuffle plasmid. Compare the growth of *mls1Δ* and *mls1Δ icl1Δ* cells on glycerol media and note that the respiratory growth rescue of *mdm30Δ* cells by the extra copy of *FZO1* is lost at 23 and 30 °C in the absence of *MLS1* but is totally recovered upon deletion of *ICL1*.
(TIF)

S5 Fig **(A)** PerMit contacts in *mls1Δ* cells. *ole1Δ* strains genomically labeled for *OM45-GFP* and *RFP-SKL* and inactivated for *MLS1* were shuffled with *OLE1*, *CYC*, or *TEF pOLE1* plasmids (*MCY1980*, *MCY1987*) and processed for whole cells imaging. The graph depicts the percentage of attachment of peroxisomes (RFP signals) to mitochondria (GFP signals) in *CYC-OLE1* (yellow bars), *OLE1* (blue bars), and *TEF-OLE1* (red bars) cells. Error bars represent the SEM from 3 independent experiments. **P < 0.05, ***P < 0.005 (one-way analysis of variance (ANOVA)). NS, not significant. Note that MLS1 inactivation does not affect the response of PerMit contacts to FA desaturation. **(B)** Percentage of cells with tubular mitochondria at 30 °C from *OLE1* (*WT*), *OLE1 mls1Δ* (*mls1Δ*), *OLE1 icl1Δ* (*icl1Δ*), and *OLE1 mls1Δ icl1Δ* (*mls1Δ icl1Δ*) shuffling strains genomically labeled for *mito-GFP* and shuffled with *OLE1*, *CYC*, or *TEF pOLE1* plasmids (*MCY1835*, *MCY1989*, *MCY2002*, *MCY2003*). Error bars represent the SEM from 3 independent experiments. **P < 0.05 (one-way analysis of variance (ANOVA)). NS, not significant. More than 100 cells per sample were analyzed. Note that the inactivation of *ICL1* in *mls1Δ* cells not only restores the tubular morphology in the absence of *MLS1* in the *CYC-OLE1* condition but also significantly improves the morphology of the mitochondrial network in the *TEF-OLE1* condition. **(C)** Right: Examples of cells genomically labeled for *mt-GFP* (*MCY1835*) with tubular mitochondrial networks; scale bar, 5 μm. Left: Percentage of cells with tubular mitochondria from *OLE1* (*WT*) and *OLE1 ctp1Δ* (*ctp1Δ*) shuffling strains genomically labeled for *mt-GFP* and shuffled with *OLE1*, *CYC*, or *TEF pOLE1* plasmids (*MCY1835*, *MCY2036*). Error bars represent the SEM from 3 independent experiments. **P < 0.05 (one-way analysis of variance (ANOVA)). More than 100 cells per sample were analyzed. Note the significant decrease in tubular mitochondria upon inactivation of *CTP1* in all conditions, including TEF-*OLE1*. This indicates that inhibiting the entry of citrate into mitochondria induces the same effects as inhibiting the synthesis of peroxisomal citrate (see Fig 5E). **(D and E)** PerMit contacts in *cat2Δ* and *cit2Δ* cells. *ole1Δ* strains inactivated for *CAT2* (C, *cat2Δ*) or *CIT2* (D, *cit2Δ*) genomically labeled for *OM45-GFP* and *RFP-SKL* were shuffled with *OLE1*, *CYC*, or *TEF pOLE1* plasmids (*MCY2054*, *MCY2052*) and processed for whole cell imaging by confocal microscopy. The graph depicts the percentage of proximity and non-proximity of peroxisomes (RFP signals) to mitochondria (GFP signals) in *CYC-OLE1* (yellow bars), *OLE1* (blue bars), and *TEF-OLE1* (red bars) cells. Error bars represent the SEM from 3 independent experiments. **P < 0.05 (one-way analysis of variance (ANOVA)). Note that inactivation of either *CAT2* or *CIT2* does not modify the amount of PerMit contact in *CYC-OLE1*, *OLE1*, or *TEF-OLE1* conditions as compared to *WT* cells (see Fig 3F). Underlying data for quantifications can be found in S1 Data.
(TIF)

S6 Fig **(A)** Time lapse acquisitions with 10 s intervals of aggregated mitochondrial networks by SIM with cells genomically labeled for *OM45-GFP* (*MCY1936*); shown are examples of aggregated network with fusion (Top) or fission (Middle) events (indicated by white arrows) or without any fusion or fission event (Bottom). Scale bar 1 μm. **(B)** In vivo mitochondrial fusion assays after mating between *ole1Δ* or *ole1Δ mls1Δ* haploid cells of opposing mating types (MCY2124 to MCY2132 and MCY2142 to MCY2150) containing *CYC-OLE1*, *OLE1*, or *TEF-OLE1* plasmids and expressing either mito-RFP or mito-GFP, respectively. (Top) Examples of zygotes with mitochondrial networks totally fused (blue), partially fused (red), or not fused (green); scale bar, 5 μm. (Bottom) Percentage of indicated zygotes with total (blue), partial (red), or no (green) mitochondrial fusion obtained from indicated cells. Error bars represent the SEM from 3 independent experiments, and 25 zygotes were analyzed per sample. Note that inactivation of *MLS1* induce notably delayed mitochondrial fusion upon low (*CYC-OLE1*) or normal (*OLE1*) FA desaturation as compared to WT zygotes. In contrast, the effects of *MLS1* inactivation on mitochondrial fusion are more limited upon high (*TEF-OLE1*) FA desaturation. Taken together, these results thus confirm the overall data obtained by time-lapse SIM acquisitions (Fig 7A). Underlying data for quantifications can be found in S1 Data.
(TIF)

S1 Table Table of *S*. *cerevisiae* strains used in this study. (DOCX)

S2 Table Table of plasmids used in this study. (DOCX)

S1 Data Data underlying quantification of imaging experiments. (XLSX)

S1 Raw Images Uncropped western blot data. (PDF)

S1 Video Mitochondrial fusion event (see Fig 7A). Fusion event analyzed by time-lapse SIM in *OM45-GFP* labeled cells. Total duration 1 min with 1 acquisition every 10 s. Scale bar 1 μm. Playback speed 1 frame per second.
(MP4)

S2 Video Mitochondrial fission event (see Fig 7B). Fission event analyzed by time-lapse structured illumination microscopy (SIM) in *OM45-GFP* labeled cells. Total duration 1 min with 1 acquisition every 10 s. Scale bar, 1 μm. Playback speed 1 frame per second.
(MP4)

## Abbreviations

BFP: blue fluorescent protein
DIC: differential interference contrast
DRP: dynamin-related protein
ER: endoplasmic reticulum
FA: fatty acid
FWHM: full width at half maximum
GFP: green fluorescent protein
HRP: horseradish peroxidase
iEM: immuno-electron microscopy
RFP: red fluorescent protein
ROI: region of interest
SGA: synthetic genetic array
SIM: structured illumination microscopy
TCA: tricarboxylic acid
UFA: unsaturated fatty acid
UPS: ubiquitin–proteasome system
