# Supplementary figures and images for: Mitofusin-mediated contacts between mitochondria and peroxisomes regulate mitochondrial fusion
Source: PLoS Biol. 2024 Apr 26;22(4):e3002602. doi: 10.1371/journal.pbio.3002602 (PMC11078399; doi:10.1371/journal.pbio.3002602)

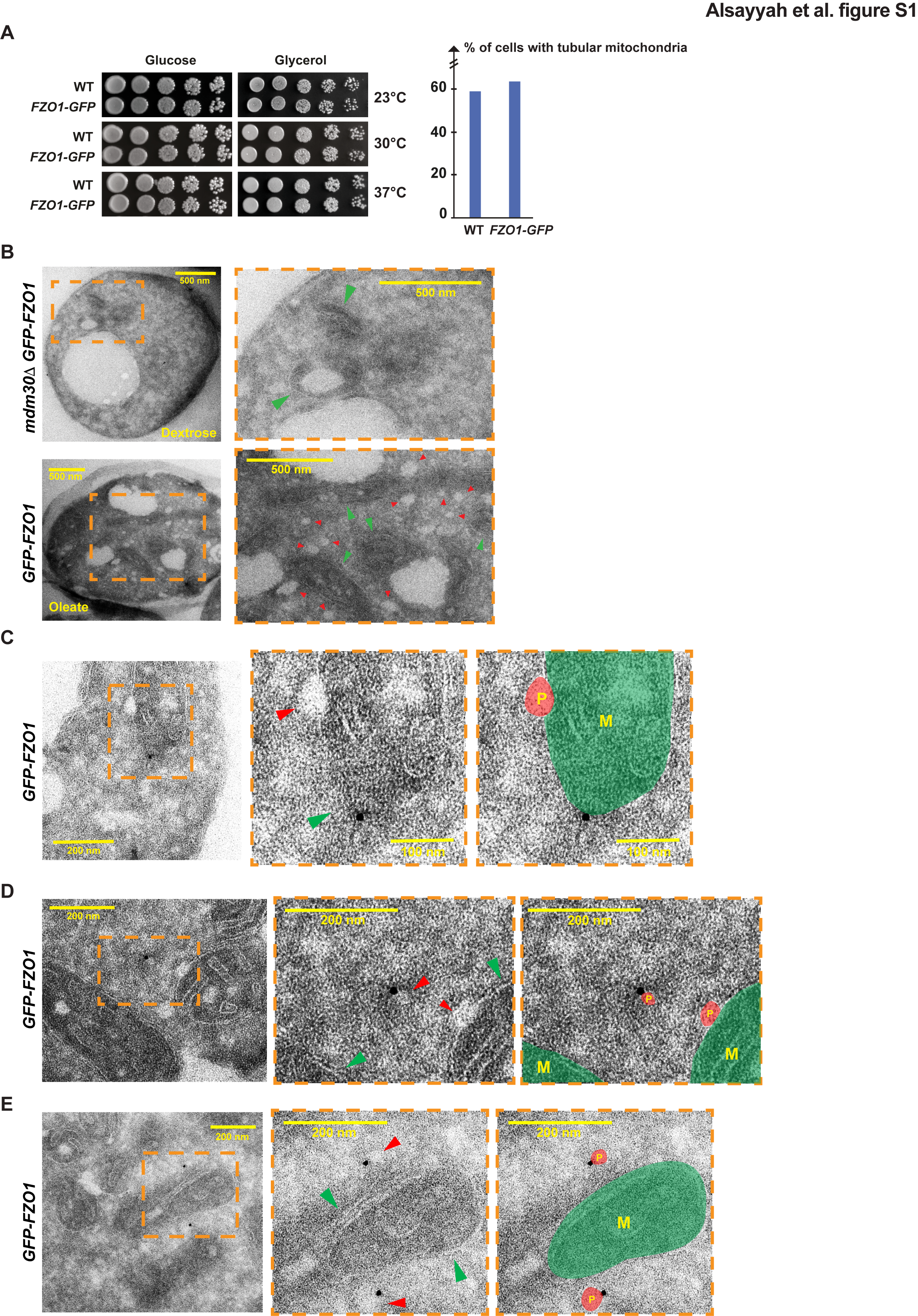

Supplement: S1 Fig — (A) Dextrose (Glucose) and Glycerol spot assays at 23, 30 and 37 °C of WT (MCY1589) and FZO1-GFP tagged strains (MCY1671) genomically labeled for mito-BFP and the corresponding percentage of cells with tubular mitochondria at 30 °C. Note that the GFP C-terminal tagging does not affect the function of Fzo1. (B) Transmission electron microscopy micrographs from mdm30Δ cells grown in Dextrose-containing media and WT cells grown in Oleate-containing media. Green arrowheads indicate mitochondria. Red arrowheads indicate circular structures with clear lumen that correspond to peroxisomes. Orange squares on left micrographs highlight the regions zoomed in on right images. Scale bars correspond to 500 nm. Note that peroxisomes proliferate and are detected in Oleate-containing media but not in Dextrose-containing media. (C, D, and E) Micrographs showing Fzo1-GFP immuno-staining on mitochondria (C) and peroxisomes (D and E) from WT cells grown in Oleate-containing media. Scale bars correspond to 200 nm. Note that Fzo1-GFP immuno-staining appears as the black dots seen on mitochondria labeled in green or peroxisomes labeled in red. Underlying data for quantifications can be found in S1 Data. (TIF) [file pbio.3002602.s001.tif]

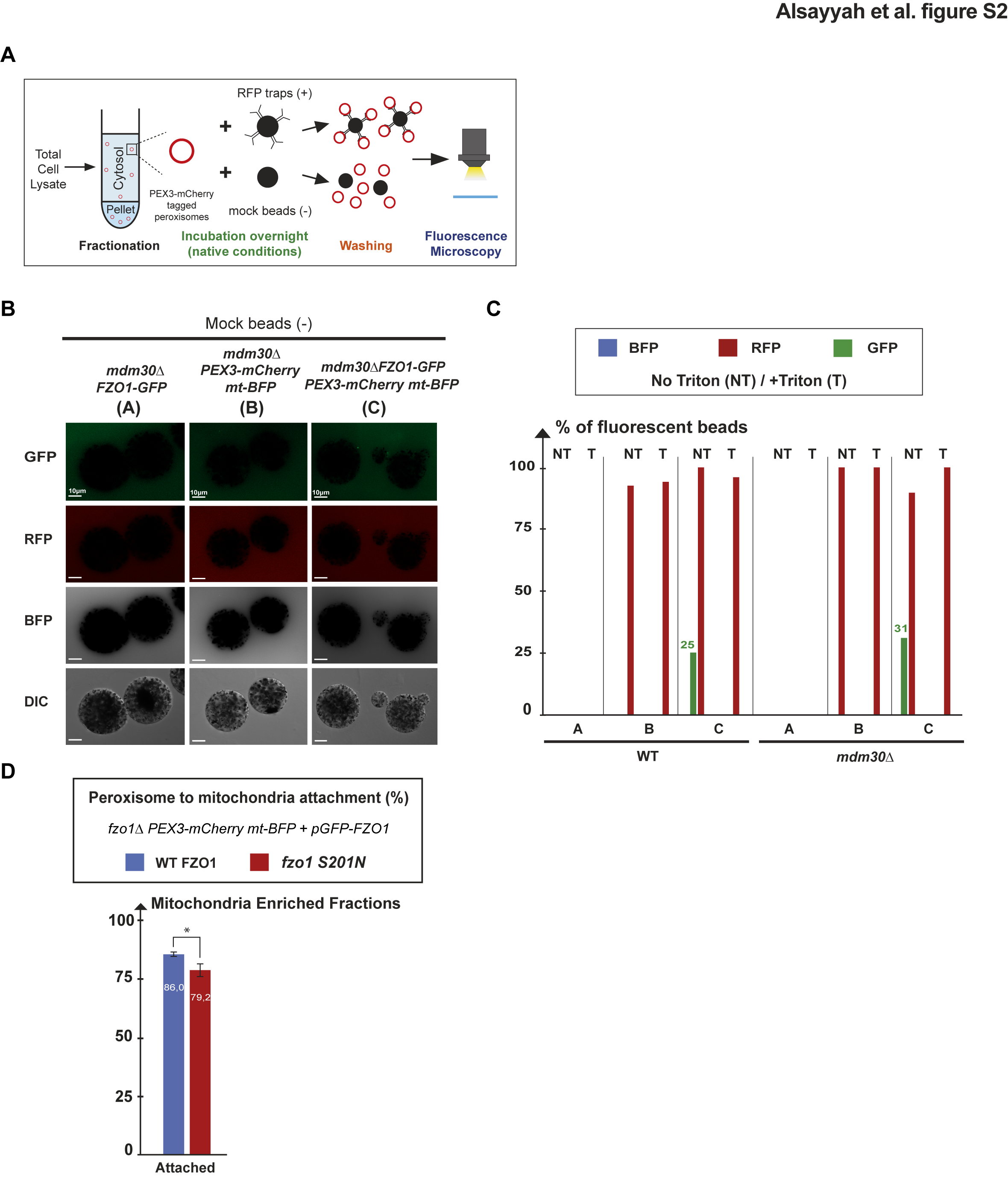

Supplement: S2 Fig — (A) Schematic representation of native Immuno-Precipitation of peroxisomes from WT and mdm30Δ cells genomically labeled for PEX3-mCherry, FZO1-GFP, and mt-BFP (MCY1667, MCY1591, MCY1675 and MCY1673, MCY1597, MCY1677 cured from the MDM30 shuffle plasmid). Cells were processed for fractionation assays to yield whole cell (Total), cytosol (Sup), and membrane (Pellet) fractions. Cytosol fractions were then split in 2 halves and incubated O.N. with mock (−) or RFP (+) Trap beads in the absence of detergent to pull-down Pex3-mCherry native peroxisomes specifically. After washing, beads were analyzed with DIC or fluorescence microscopy for detection of Fzo1 (GFP), Pex3 (RFP), and mitochondria (BFP). (B) DIC and fluorescence microscopy analysis of mock beads after washing (see also Fig 1E for RFP Trap beads). Scale bars correspond to 10 μm. Note that GFP (green), RFP (red), or BFP (gray) signals are not detected, indicating that peroxisomes (Pex3-mCherry), mitochondria (mt-BFP), or Fzo1-GFP do not bind nonspecifically to the beads. (C) Same experiment as in Fig 1F but washing of RFP Trap beads was performed either in the absence (NT) or in the presence (T) of Triton detergent. The graph shows the percentage of beads with BFP (blue), mCherry (red), or GFP (green) signal in each condition. Note that Fzo1-GFP is detected in the NT condition, in the absence of BFP signal and thus in the absence of mitochondria, but that this Fzo1-GFP is lost in the presence of detergent. This confirms that Fzo1 is embedded in peroxisomes from WT and mdm30Δ cells. (D) Percentage of peroxisomal attachment (mCherry signals) to mitochondria (GFP/BFP signals) in Mitochondrial Enriched Fractions from WT (blue bars) and FZO1-S201N (red bars) cells labeled for both PEX3-mCherry, mito-BFP, and FZO1-GFP (MCY1771 and MCY1772). Error bars represent the SEM from 3 independent experiments. * P = 0.05 (one-way analysis of variance (ANOVA)). Underlying data for quantifications can be found in S1 Data. (TIF) [file pbio.3002602.s002.tif]

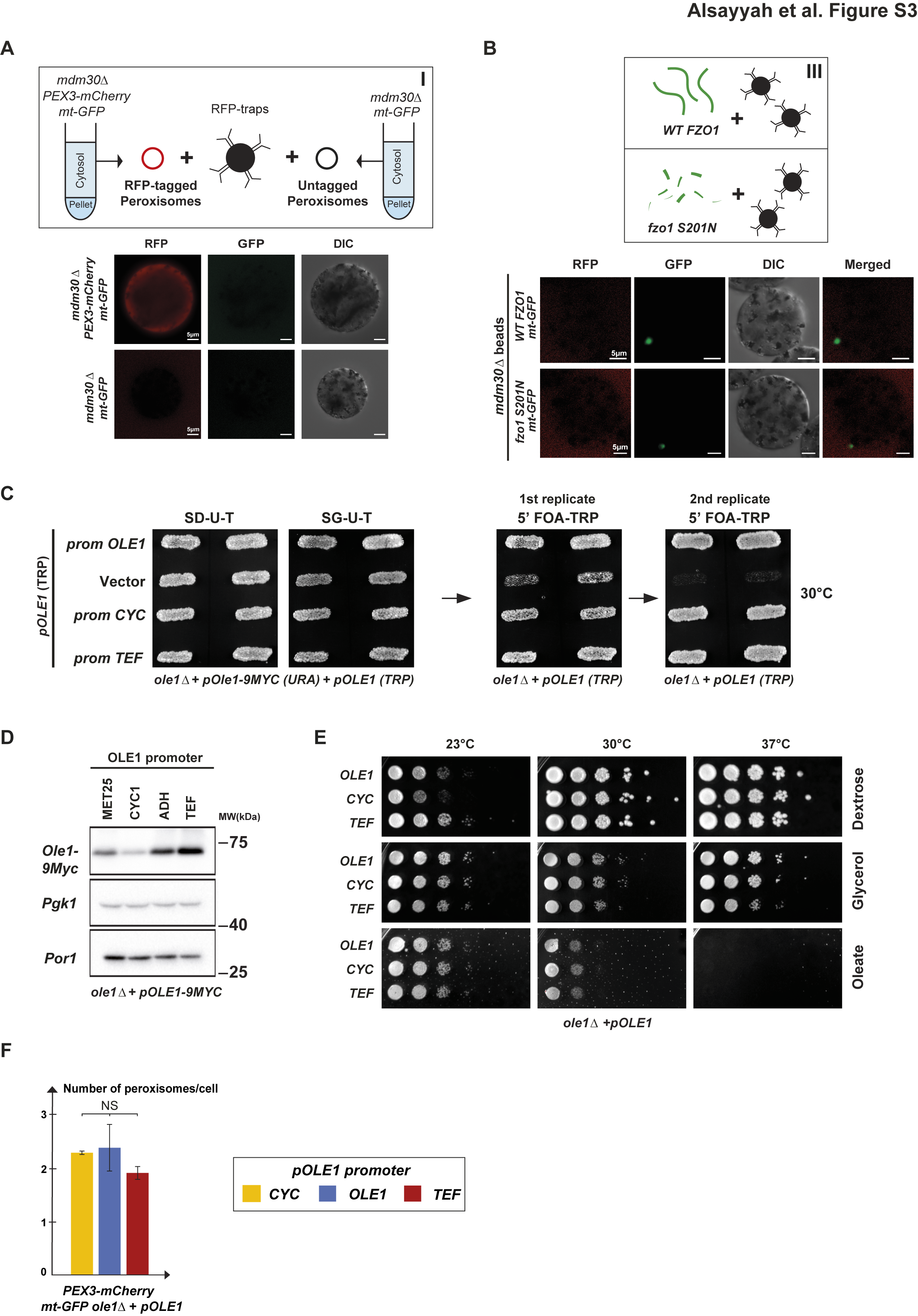

Supplement: S3 Fig — (A) Schematic representation of ex vivo PerMit contact assays (Panel I). DIC and fluorescence microscopy analysis of RFP Trap beads after overnight incubation with the cytosolic fractions of mdm30Δ mito-GFP cells either genomically labeled or unlabeled for PEX3-mCherry (MCY1842 and MCY1847 cured from the MDM30 shuffle plasmid). Note the negative signal for GFP, confirming that mitochondria are absent from cytosolic fractions and do not pull-down with peroxisomes. (B) Ex vivo PerMit contact assays (Panel III). DIC and fluorescence microscopy analysis of untagged peroxisomes RFP Trap beads from S1A (bottom row) after overnight incubation with the membrane fractions of WT FZO1 and FZO1 S201N cells genomically labeled for mito-GFP (MCY1843 transformed with pRS314-FZO1 (MC250) or pRS314-FZO1-S201N (MC544)). Note the positive signal for GFP that reflects the nonspecific binding of mitochondria to RFP Traps and that was quantified and subtracted from the specific binding shown in Fig 2E. (C, D, and E) Preparation and characterization of OLE1 shuffle strains. (C) ole1Δ cells covered by a pOLE1-9MYC shuffle plasmid with URA3 selection were transformed with an empty vector or with pOLE1 plasmids under control of OLE1, CYC, or TEF promoters with TRP1 selection. Resulting double transformants were patched on Synthetic Dextrose media without Uracil and Tryptophan (SD -U-T) and replica-plated on Synthetic Glycerol media without Uracil and Tryptophan (SG -U-T) or on 5-FOA media without Tryptophan (5-FOA -TRP) to initiate curation of the pOLE1-9MYC shuffle plasmid with URA3 selection. Note that after a second replicate on 5-FOA media without Tryptophan, the growth of the empty vector strain was abolished as expected since OLE1 is essential for viability. (D) Whole cell extracts of ole1Δ strains shuffled with MET25, CYC, ADH, or TEF pOLE1-9MYC plasmids (MCY1798, MCY1797, MCY1796, MCY1795) were processed for western blotting with anti-Myc, anti-PGK, and anti-Porin. Molecular weights [file pbio.3002602.s003.tif]

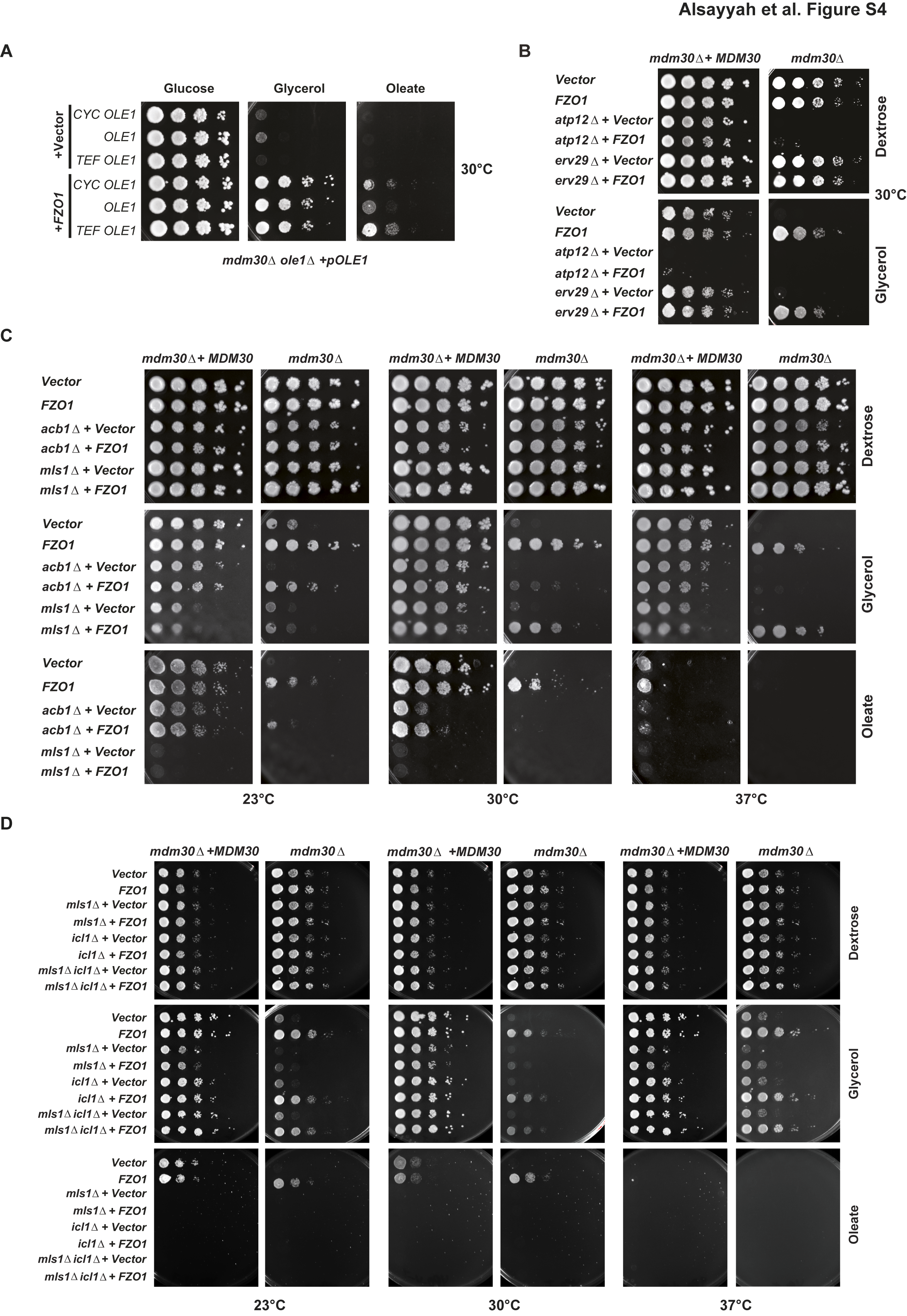

Supplement: S4 Fig — (A) Dextrose, Glycerol, and Oleate spot assays at 30 °C of ole1Δ mdm30Δ strains (MCY1959) transformed with pRS314-FZO1 (MC250) or an empty vector (MC219) and shuffled with OLE1, CYC, or TEF pOLE1 plasmids (MC540, MC541, MC543). Note that the extra copy of FZO1 not only rescues the growth of mdm30Δ cells on glycerol but also on Oleate, suggesting a stimulation of peroxisomal function. (B) Examples of false positive hits of the primary screen that were characterized in the secondary screen. Dextrose and glycerol spot assays at 30 °C of MDM30 (MCY970), MDM30 atp12Δ (MCY1616), and MDM30 erv29Δ (MCY1610) shuffling strains transformed with pRS314-FZO1 (MC250) or an empty vector (MC219) and covered by (mdm30Δ + MDM30) or cured from (mdm30Δ) the MDM30 shuffle plasmid. Absence of ATP12 (a factor required for assembly of the ATP synthase) abolishes the respiratory rescue of mdm30Δ cells by FZO1 but also blocks the respiratory growth of MDM30 positive cells. Absence of ERV29 (a factor involved in COPII vesicles formation) does not affect the respiratory rescue of mdm30Δ cells by FZO1. (C) Confirmed candidates after the secondary screen. Dextrose, Glycerol, and Oleate spot assays at 23, 30 and 37 °C of MDM30 (MCY970), MDM30 acb1Δ (MCY1612), and MDM30 mls1Δ (MCY1649) shuffling strains transformed with pRS314-FZO1 (MC250) or an empty vector (MC219) and covered by (mdm30Δ + MDM30) or cured from (mdm30Δ) the MDM30 shuffle plasmid. Note that the absence of ACB1 affects the respiratory rescue of mdm30Δ cells by FZO1 at higher temperatures (30 and 37 °C), whereas the absence of MLS1 does so at lower temperatures (23 and 30 °C). Interestingly, the absence of ACB1 also affects the growth rescue of mdm30Δ cells by FZO1 on Oleate at 23 and 30 °C. As expected, absence of MLS1 abolishes the growth of all cells on Oleate media. (D) Dextrose, Glycerol, and Oleate spot assays at 23, 30 and 37 °C of MDM30 (MCY970), MDM30 mls1Δ (MCY1649), MDM30 icl1Δ (MCY1909), and MDM30 mls1Δ icl1Δ (MCY1911) sh [file pbio.3002602.s004.tif]

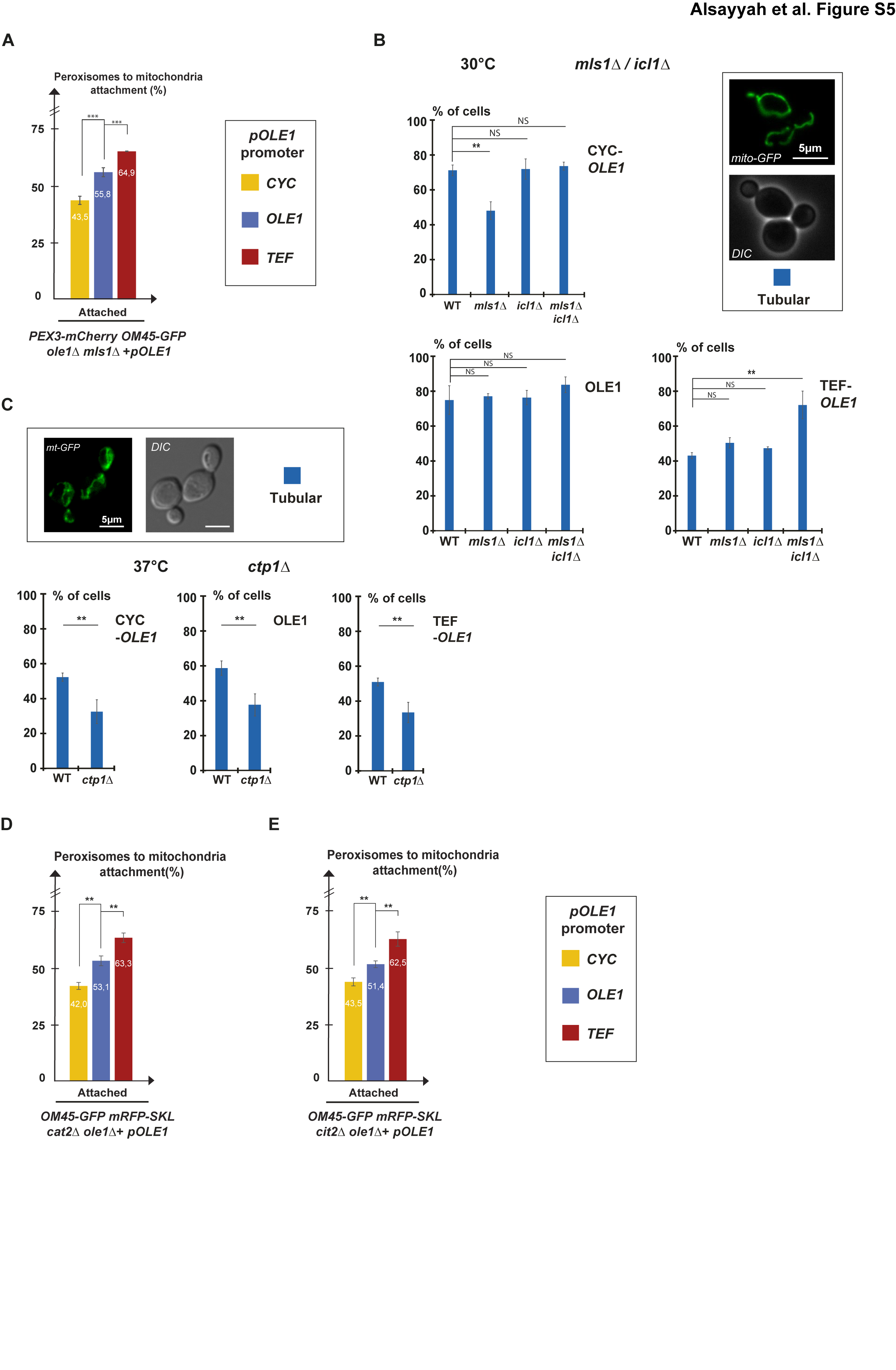

Supplement: S5 Fig — (A) PerMit contacts in mls1Δ cells. ole1Δ strains genomically labeled for OM45-GFP and RFP-SKL and inactivated for MLS1 were shuffled with OLE1, CYC, or TEF pOLE1 plasmids (MCY1980, MCY1987) and processed for whole cells imaging. The graph depicts the percentage of attachment of peroxisomes (RFP signals) to mitochondria (GFP signals) in CYC-OLE1 (yellow bars), OLE1 (blue bars), and TEF-OLE1 (red bars) cells. Error bars represent the SEM from 3 independent experiments. **P < 0.05, ***P < 0.005 (one-way analysis of variance (ANOVA)). NS, not significant. Note that MLS1 inactivation does not affect the response of PerMit contacts to FA desaturation. (B) Percentage of cells with tubular mitochondria at 30 °C from OLE1 (WT), OLE1 mls1Δ (mls1Δ), OLE1 icl1Δ (icl1Δ), and OLE1 mls1Δ icl1Δ (mls1Δ icl1Δ) shuffling strains genomically labeled for mito-GFP and shuffled with OLE1, CYC, or TEF pOLE1 plasmids (MCY1835, MCY1989, MCY2002, MCY2003). Error bars represent the SEM from 3 independent experiments. **P < 0.05 (one-way analysis of variance (ANOVA)). NS, not significant. More than 100 cells per sample were analyzed. Note that the inactivation of ICL1 in mls1Δ cells not only restores the tubular morphology in the absence of MLS1 in the CYC-OLE1 condition but also significantly improves the morphology of the mitochondrial network in the TEF-OLE1 condition. (C) Right: Examples of cells genomically labeled for mt-GFP (MCY1835) with tubular mitochondrial networks; scale bar, 5 μm. Left: Percentage of cells with tubular mitochondria from OLE1 (WT) and OLE1 ctp1Δ (ctp1Δ) shuffling strains genomically labeled for mt-GFP and shuffled with OLE1, CYC, or TEF pOLE1 plasmids (MCY1835, MCY2036). Error bars represent the SEM from 3 independent experiments. **P < 0.05 (one-way analysis of variance (ANOVA)). More than 100 cells per sample were analyzed. Note the significant decrease in tubular mitochondria upon inactivation of CTP1 in all conditions, including TEF-OLE1. This indicates that in [file pbio.3002602.s005.tif]

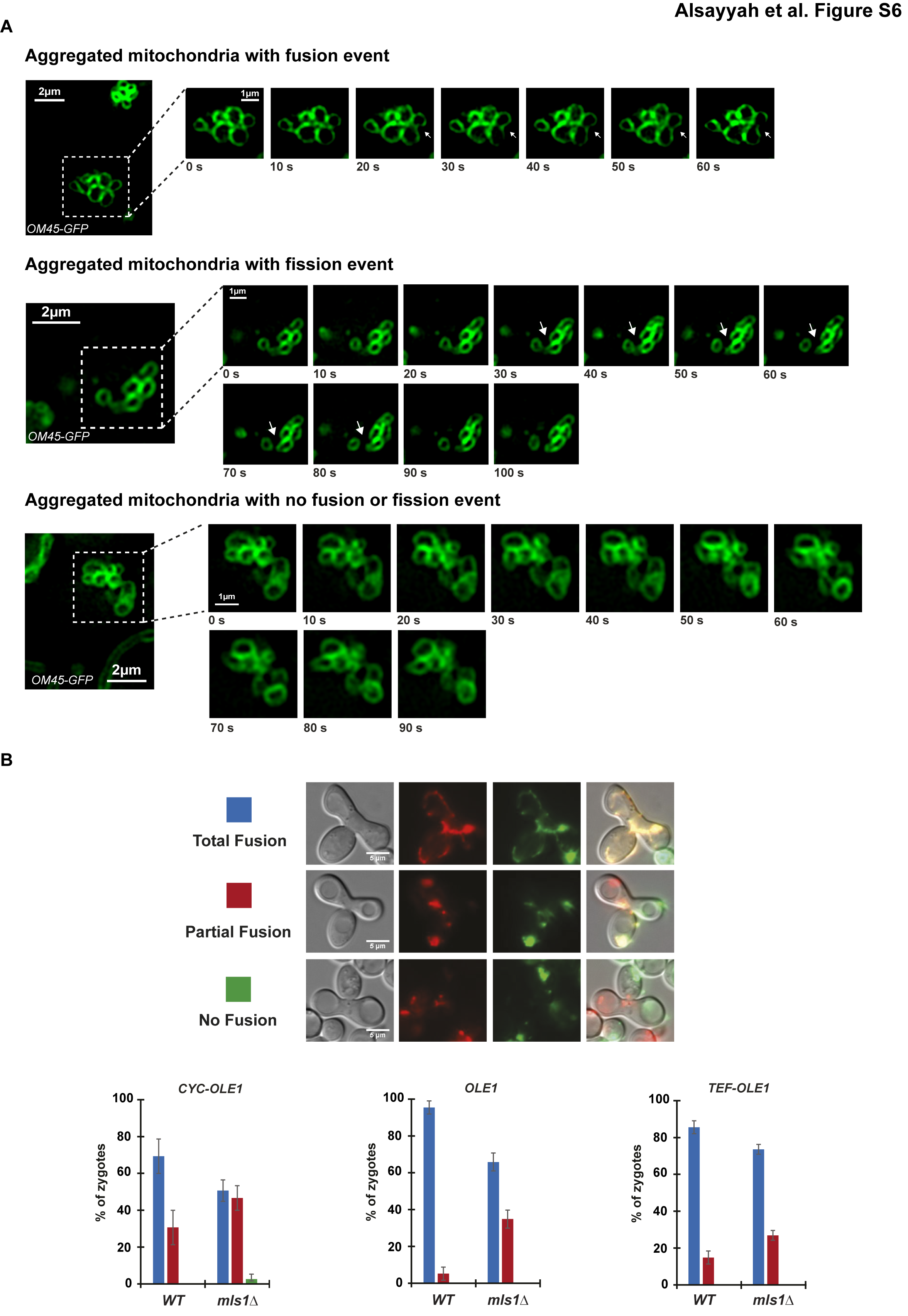

Supplement: S6 Fig — (A) Time lapse acquisitions with 10 s intervals of aggregated mitochondrial networks by SIM with cells genomically labeled for OM45-GFP (MCY1936); shown are examples of aggregated network with fusion (Top) or fission (Middle) events (indicated by white arrows) or without any fusion or fission event (Bottom). Scale bar 1 μm. (B) In vivo mitochondrial fusion assays after mating between ole1Δ or ole1Δ mls1Δ haploid cells of opposing mating types (MCY2124 to MCY2132 and MCY2142 to MCY2150) containing CYC-OLE1, OLE1, or TEF-OLE1 plasmids and expressing either mito-RFP or mito-GFP, respectively. (Top) Examples of zygotes with mitochondrial networks totally fused (blue), partially fused (red), or not fused (green); scale bar, 5 μm. (Bottom) Percentage of indicated zygotes with total (blue), partial (red), or no (green) mitochondrial fusion obtained from indicated cells. Error bars represent the SEM from 3 independent experiments, and 25 zygotes were analyzed per sample. Note that inactivation of MLS1 induce notably delayed mitochondrial fusion upon low (CYC-OLE1) or normal (OLE1) FA desaturation as compared to WT zygotes. In contrast, the effects of MLS1 inactivation on mitochondrial fusion are more limited upon high (TEF-OLE1) FA desaturation. Taken together, these results thus confirm the overall data obtained by time-lapse SIM acquisitions (Fig 7A). Underlying data for quantifications can be found in S1 Data. (TIF) [file pbio.3002602.s006.tif]

Figure 1D

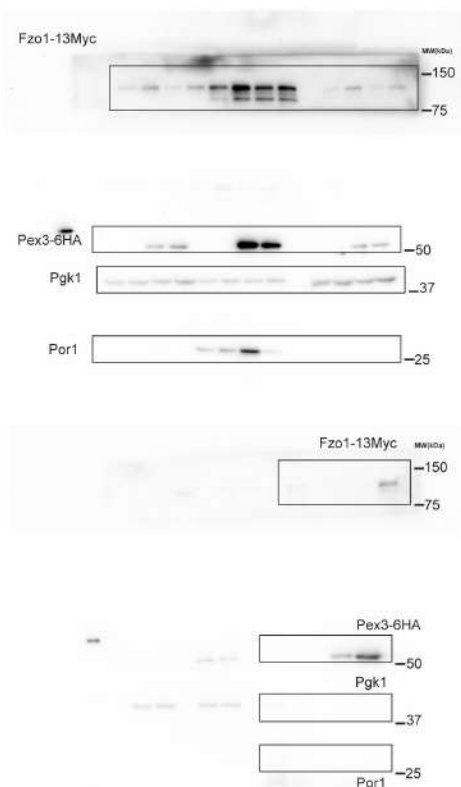

Figure 2A

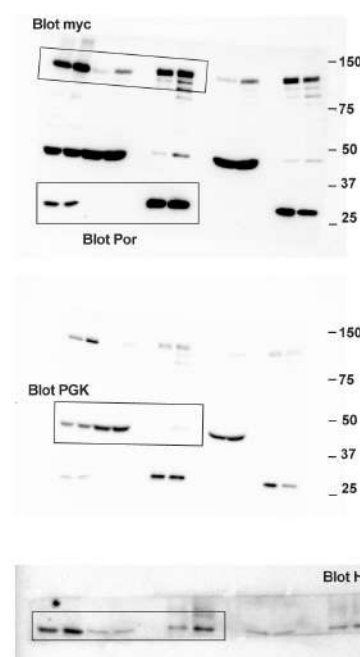

Figure 3C

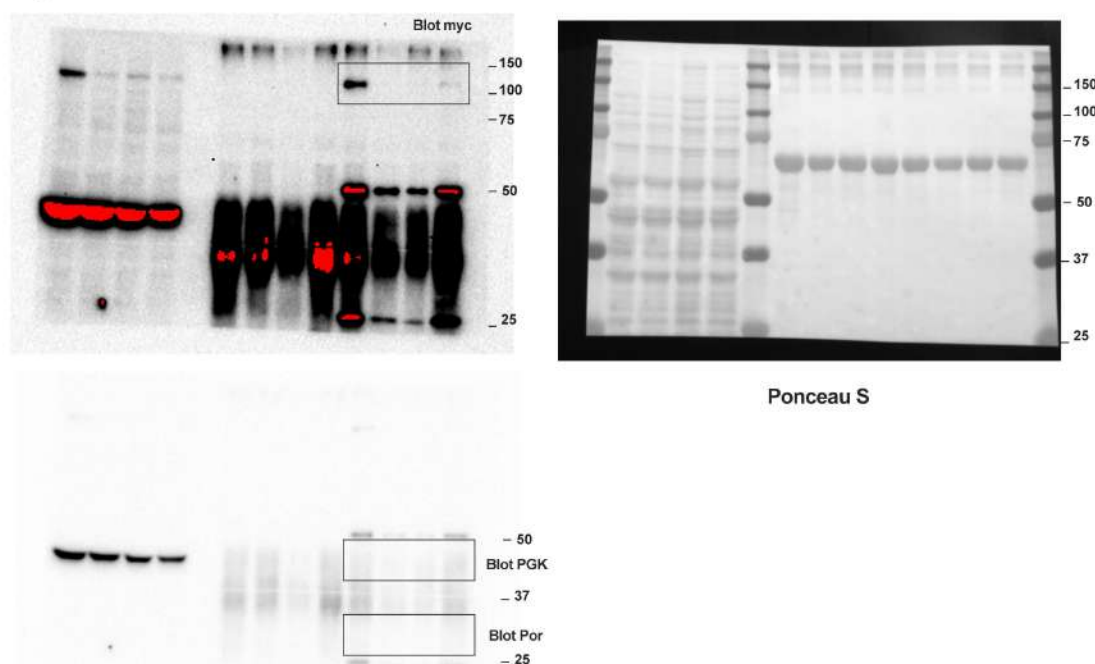

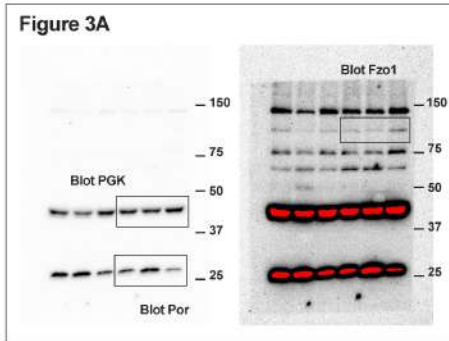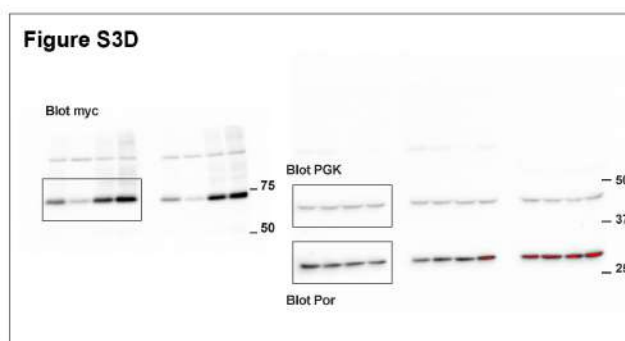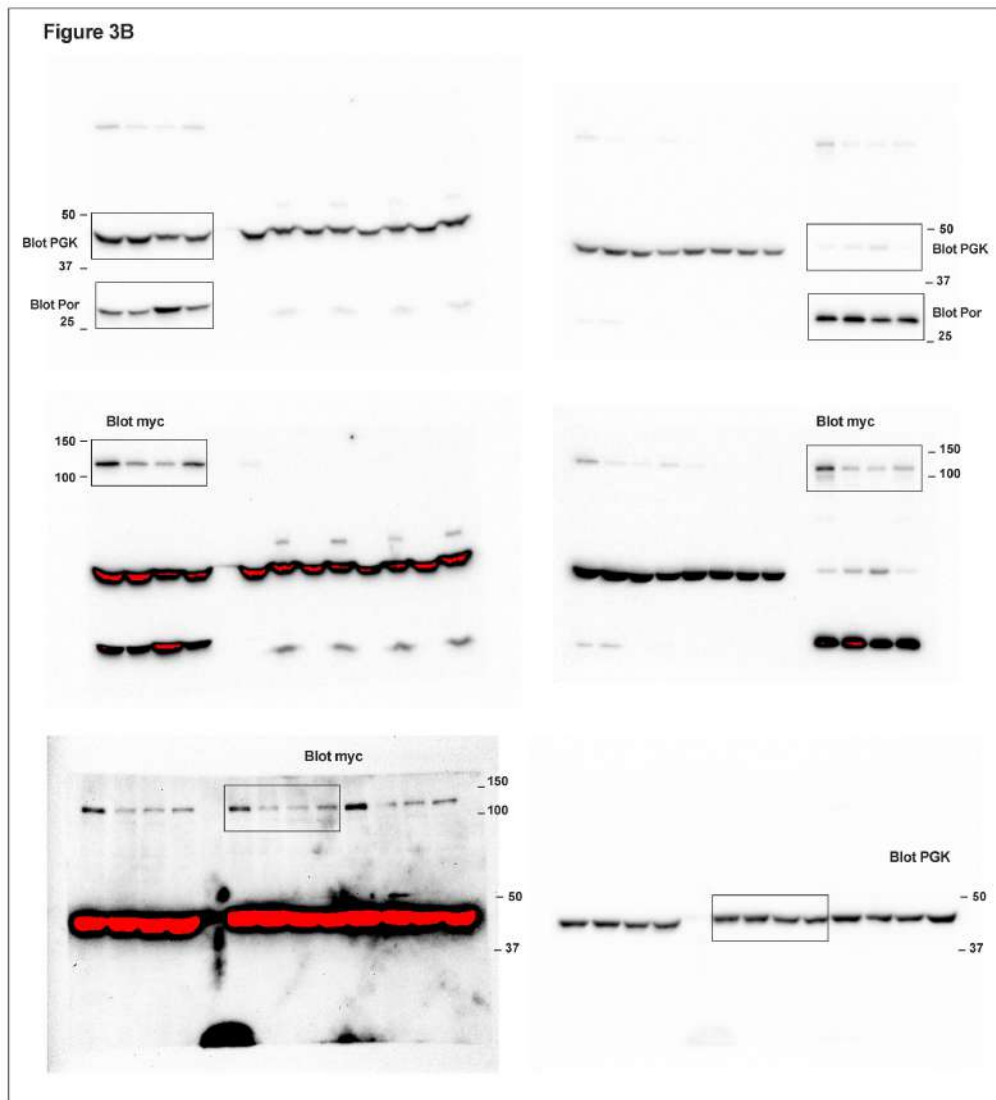

Supplement: S1 Raw Images — (PDF) [file pbio.3002602.s010.pdf]
