# Supplementary material for: Mitofusin-mediated contacts between mitochondria and peroxisomes regulate mitochondrial fusion
Source: PLoS Biol. 2024 Apr 26;22(4):e3002602. doi: 10.1371/journal.pbio.3002602 (PMC11078399; doi:10.1371/journal.pbio.3002602)
Supplement: S1 Table — (DOCX) [file pbio.3002602.s007.docx]

**Supplementary Table 1: S. cerevisiae strains used in this study.**

| **Name** | **Parental strains** | **Genotype** | **Occurrence**  **In the study** | **Reference** |
| --- | --- | --- | --- | --- |
| W303 WT (MCY553) | W303 | *Mata ura3-1 trp1-1 leu2-3,112 his3-11,15 can1-100 RAD5 ADE2* | Used to build FZO1  and MDM30  shuffle strains | Gift from T. Teixeira |
| W303 WT (MCY554) | W303 | *MATα ura3-1 trp1-1 leu2-3,112 his3-11,15 can1-100 RAD5 ADE2* |  | Gift from T. Teixeira |
| *FZO1-GFP (MCY1667)* | W303 (MCY553) | *Mata ura3-1 trp1-1 leu2-3,112 his3-11,15 can1-100 RAD5 ADE2 FZO1-EGFP::KANMX6* | 1E, 1F, 1G, 1H,S2A, S2B, S2C | This study |
| *PEX3-mCherry mt-BFP (MCY 1591)* | W303 (MCY553) | *Mata ura3-1 trp1-1 leu2-3,112 his3-11,15 can1-100 RAD5 ADE2 PEX3-mCherry::NAT mt-BFP::LEU2* | 1E, 1F, 1G, 1H,S2A, S2B, S2C | This study |
| *FZO1-GFP PEX3-mCherry mt-BFP (MCY1675)* | W303 (MCY553) | *Mata ura3-1 trp1-1 leu2-3,112 his3-11,15 can1-100 RAD5 ADE2 PEX3-mCherry::NAT mt-BFP::LEU2 FZO1-EGFP::KANMX6* | 1A,1B,1E,1F, 1G,1H, S1A,S2A, S2B, S2C | This study |
| *mdm30∆ FZO1-GFP +pMDM30 (MCY1673)* | W303 (MCY970) | *Mata ura3-1 trp1-1 leu2-3,112 his3-11,15 can1-100 RAD5 ADE2 MDM30::LoxP FZO1-EGFP::KANMX6 + PRS316-MDM30* | 1E, 1F, 1G,1H,S2A, S2B, S2C | This study |
| *mdm30∆ PEX3-mCherry mt-BFP*  *+pMDM30*  *(MCY 1597)* | W303 (MCY970) | *Mata ura3-1 trp1-1 leu2-3,112 his3-11,15 can1-100 RAD5 ADE2 MDM30::KANMX6 PEX3-mCherry::NAT mt-BFP::LEU2 + PRS316-MDM30* | 1E, 1F,1G,1H, S2A, S2B, S2C | This study |
| *mdm30∆ FZO1-GFP PEX3-mCherry mt-BFP +pMDM30 (MCY1677)* | W303 (MCY970) | *Mata ura3-1 trp1-1 leu2-3,112 his3-11,15 can1-100 RAD5 ADE2 MDM30::LoxP PEX3-mCherry::NAT mt-BFP::LEU2 FZO1-EGFP::KANMX6 + PRS316-MDM30* | 1A,1B,1E,1F,1G,1H, S2A, S2B, S2C | This study |
| *FZO1-13MYC PEX3-6HA (MCY1488)* | W303 (MCY553) | *Mata ura3-1 trp1-1 leu2-3,112 his3-11,15 can1-100 RAD5 ADE2 FZO1-13MYC::HIS5+ PEX3-6HA::NAT* | 1D,3B,3C | This study |
| *mdm30∆ FZO1-13MYC PEX3-6HA (MCY1490)* | W303 (MCY971) | *MATα ura3-1 trp1-1 leu2-3,112 his3-11,15 can1-100 RAD5 ADE2 MDM30::KANMX6 FZO1-13MYC::HIS5 PEX3-6HA::NAT* | 1D,3B,3C | This study |
| *fzo1∆ + pFZO1 (URA) (MCY572)* | W303 | *MATα ura3-1 trp1-1 leu2-3,112 his3-11,15 can1-100 RAD5 ADE2 FZO1::LEU2 +PRS416-FZO1* |  | [1] |
| *fzo1∆ PEX3-6HA +pFZO1-13MYC (MCY1779)* | W303 (MCY572) | *MATα ura3-1 trp1-1 leu2-3,112 his3-11,15 can1-100 RAD5 ADE2 FZO1::LEU2 PEX3-6HA::Nat +PRS414-FZO1-13MYC* | 2A | This study |
| *fzo1∆ PEX3-6HA +pFZO1S201N-13MYC (MCY1780)* | W303 (MCY572) | *MATα ura3-1 trp1-1 leu2-3,112 his3-11,15 can1-100 RAD5 ADE2 Pex3-6HA::NAT FZO1::LEU2 +PRS414-FZO1S201N-13MYC* | 2A | This study |
| *fzo1∆ +pFzo1-URA (MCY1569)* | W303 | *Mata FZO1::LoxP ura3-1 trp1-1 leu2-3,112 his3-11,15 can1-100 RAD5 ADE2 +PRS416-FZO1* |  | This study |
| *fzo1∆ PEX3-mCherry mt-BFP + pGFP-Link-FZO1 prom ADH (MCY1802)* | W303 (MCY1569) | *Mata FZO1::LoxP ura3-1 trp1-1 leu2-3,112 his3-11,15 can1-100 RAD5 ADE2 PEX3-mCherry::NAT mt-BFP::LEU2 +PRS414-GFP-FZO1* | 2B | This study |
| *fzo1∆ PEX3-mCherry mt-BFP +pGFP-Link-FZO1S201N prom ADH (MCY1803)* | W303 (MCY1569) | *Mata FZO1::LoxP ura3-1 trp1-1 leu2-3,112 his3-11,15 can1-100 RAD5 ADE2 PEX3-mCherry::NAT mt-BFP::LEU2 +PRS414-GFP-FZO1S201N* | 2B | This study |
| *fzo1∆ mt-BFP + pGFP-Link-FZO1 prom ADH (MCY1804)* | W303 (MCY1569) | *Mata FZO1::LoxP ura3-1 trp1-1 leu2-3,112 his3-11,15 can1-100 RAD5 ADE2 mt-BFP::LEU2 +PRS414-GFP-FZO1* | 2B | This study |
| *fzo1∆ mt-BFP + pGFP-Link-FZO1S201N prom ADH (MCY1805)* | W303 (MCY1569) | *Mata FZO1::LoxP ura3-1 trp1-1 leu2-3,112 his3-11,15 can1-100 RAD5 ADE2 mtBFP::LEU2 +PRS414-GFP-FZO1S201N* | 2B | This study |
| *fzo1∆ PEX3-mCherry mt-BFP + pGFP-Link-FZO1 (MCY1771)* | W303 (MCY1569) | *Mata FZO1::LoxP ura3-1 trp1-1 leu2-3,112 his3-11,15 can1-100 RAD5 ADE2 PEX3-mCherry::NAT mt-BFP::LEU2 +PRS414-GFP-FZO1* | 2C,S2D | This study |
| *fzo1∆ PEX3-mCherry mt-BFP +pGFP-Link-FZO1S201N (MCY1772)* | W303 (MCY1569) | *Mata FZO1::LoxP ura3-1 trp1-1 leu2-3,112 his3-11,15 can1-100 RAD5 ADE2 Pex3-mCherry::NAT mt-BFP::LEU2 + PRS414-GFP-FZO1S201N* | 2C,S2D | This study |
| *mdm30∆ mt-GFP +pMDM30 (MCY1842)* | W303 (MCY970) | *Mata ura3-1 trp1-1 leu2-3,112 his3-11,15 can1-100 RAD5 ADE2 MDM30::KANMX6 mt-GFP::LEU2 + PRS316-MDM30* | 2E,2F, S3A, S3B | This study |
| *fzo1∆ mt-GFP +pFZO1 (URA) (MCY1843)* | W303 (MCY1569) | *Mata FZO1::LoxP ura3-1 trp1-1 leu2-3,112 his3-11,15 can1-100 RAD5 ADE2 mt-GFP::LEU2 +PRS416-FZO1* | 2E,2F, S3B | This study |
| *mdm30∆ PEX3-mCherry mt-GFP +pMDM30 (MCY1847)* | W303 (MCY1842) | *Mata ura3-1 trp1-1 leu2-3,112 his3-11,15 can1-100 RAD5 ADE2 MDM30::KANMX6 mt-GFP::LEU2 Pex3-mCherry::NAT + PRS316-MDM30* | 2E,2F, S3A | This study |
| *ole1∆ + pOLE1-9MYC (MCY1781)* | W303 (MCY553) | *Mata ura3-1 trp1-1 leu2-3,112 his3-11,15 can1-100 RAD5 ADE2 OLE1::KANMX6 + PRS416-CYC-OLE1-9MYC* | S3E | This study |
| *ole1∆ mt-GFP PEX3-mCherry + pOLE1 prom OLE1 (MCY1861)* | W303 (MCY1781) | *Mata ura3-1 trp1-1 leu2-3,112 his3-11,15 can1-100 RAD5 ADE2 OLE1::KANMX6 mt-GFP::LEU2 PEX3-mCherry::NAT + PRS414-OLE1-OLE1* | 3A,3D,S3F,3E | This study |
| *ole1∆ mt-GFP PEX3-mCherry + pOLE1 prom CYC (MCY1863)* | W303 (MCY1781) | *Mata ura3-1 trp1-1 leu2-3,112 his3-11,15 can1-100 RAD5 ADE2 OLE1::KANMX6 mt-GFP::LEU2 PEX3-mCherry::NAT + PRS414-CYC-OLE1* | 3A,3D, S3F,3E | This study |
| *ole1∆ mt-GFP PEX3-mCherry + pOLE1 prom TEF (MCY1865)* | W303 (MCY1781) | *Mata ura3-1 trp1-1 leu2-3,112 his3-11,15 can1-100 RAD5 ADE2 OLE1::KANMX6 mt-GFP::LEU2 PEX3-mCherry::NAT + PRS414-TEF-OLE1* | 3A,3D, S3F,3E | This study |
| *ole1∆ FZO1-13MYC PEX3-6HA + pOLE1-9MYC (MCY1785)* | W303 (MCY1488) | *Mata ura3-1 trp1-1 leu2-3,112 his3-11,15 can1-100 RAD5 ADE2 FZO1-13MYC::HIS5 PEX3-6HA::NAT OLE1::KANMX6 + PRS416-ADH OLE1-9MYC* | 3B,3C | This study |
| *mdm30∆ ole1∆ OM45-GFP RFP-SKL +pOLE1-9MYC (MCY1959)* | W303 (MCY1490) | *MATα ura3-1 trp1-1 can1-100 RAD5 ADE2 OLE1::KAN OM45-EGFP::HIS RFP-SKL::LEU MDM30::NAT + PRSRS416-CYC-OLE1-9MYC* | 3E, S4A | This study |
| *mdm30∆ +pMDM30 (MCY970)* | W303 | *Mata ura3-1 trp1-1 leu2-3,112 his3-11,15 can1-100 MDM30::KANMX6 + PRS316-MDM30* | 4B,4D,4E,5C, S4B, S4C, S4D | [1] |
| alpha SGA ready strain yMS721 (MCY1510) | BY4741 | *MATα his3Δ1 leu2Δ0 met15Δ0ura3Δ0 can1Δ::STE2pr-spHIS5 lyp1Δ::STE3pr-LEU2* |  | gift from M. Schuldiner |
| *yMS721 +pFZO1 (MCY1513)* | BY4741 (MCY1510) | *MATα his3Δ1 leu2Δ0 met15Δ0ura3Δ0 can1Δ::STE2pr-spHIS5 lyp1Δ::STE3pr-LEU2 + PRS416-FZO1* | 4C | This study |
| *yMS721 mdm30∆ +pFZO1 (MCY1528)* | BY4741 (MCY1510) | *MATα his3Δ1 leu2Δ0 met15Δ0ura3Δ0 can1Δ::STE2pr-spHIS5 lyp1Δ::STE3pr-LEU2 MDM30::NAT + PRS416-FZO1* | 4C | This study |
| *mdm30∆ acb1∆ +pMdm30 (MCY1612)* | W303 (MCY970) | *Mata ura3-1 trp1-1 leu2-3,112 his3-11,15 can1-100 RAD5 ADE2 MDM30::KANMX6 ACB1::NAT + PRS316-MDM30* | 4D,S4C | This study |
| *mdm30∆ mls1∆ +pMDM30 (MCY1649)* | W303 (MCY970) | *Mata ura3-1 trp1-1 leu2-3,112 his3-11,15 can1-100 RAD5 ADE2 MDM30::KANMX6 MLS1::NAT + PRS316-MDM30* | 4E,5C, S4C, S4D | This study |
| *ole1∆ OM45-GFP RFP-SKL +pOLE1-9MYC (MCY1899)* | W303 (MCY1781) | *Mata ura3-1 trp1-1 leu2-3,112 his3-11,15 can1-100 RAD5 ADE2 OLE1::KANMX6 OM45-EGFP::HIS5 RFP-SKL::LEU2 + PRS416-CYC-OLE1-9MYC* | 5B,7A,7B, S6A | This study |
| *ole1∆ mls1∆ OM45-GFP RFP-SKL +pOLE1-9MYC (MCY1987)* | W303 (MCY1899) | *Mata ura3-1 trp1-1 leu2-3,112 his3-11,15 can1-100 RAD5 ADE2 OLE1::KANMX6 OM45-EGFP::HIS5 RFP-SKL::LEU2 MLS1::NAT +PRS416-CYC-OLE1-9MYC* | S5A, 5B, 7A, 6B, 7C | This study |
| *mdm30∆ mls1∆ icl1∆ +pMDM30 (MCY1911)* | W303 (MCY970) | *Mata ura3-1 trp1-1 leu2-3,112 his3-11,15 can1-100 RAD5 ADE2 MDM30::KANMX6 MLS1::NAT ICL1::HIS5 +PRS316-MDM30* | 5C, S4D | This study |
| *ole1∆ mt-GFP +pOLE1-9MYC (MCY1835)* | W303 (MCY1781) | *Mata ura3-1 trp1-1 leu2-3,112 his3-11,15 can1-100 RAD5 ADE2 OLE1::KANMX6 mt-GFP::LEU2 + PRS416-CYC-OLE1-9MYC* | 5B,S5B | This study |
| *ole1∆ mls1∆ mt-GFP +pOLE1-9MYC (MCY1989)* | W303 (MCY1835) | *Mata ura3-1 trp1-1 leu2-3,112 his3-11,15 can1-100 RAD5 ADE2 OLE1::KANMX6 mt-GFP::LEU2 MLS1::NAT + PRS416-CYC-OLE1-9MYC* | 5B, S5B | This study |
| *ole1∆ icl1∆ mt-GFP +pOLE1-9MYC (MCY2002)* | W303 (MCY1835) | *Mata ura3-1 trp1-1 leu2-3,112 his3-11,15 can1-100 RAD5 ADE2 OLE1::KANMX6 mt-GFP::LEU2 ICL1::NAT + PRS416-CYC-OLE1-9MYC* | 5B, S5B | This study |
| *ole1∆ mls1∆ icl1∆ mt-GFP +pOLE1-9MYC (MCY2003)* | W303 (MCY1835) | *Mata ura3-1 trp1-1 leu2-3,112 his3-11,15 can1-100 RAD5 ADE2 OLE1::KANMX6 mt-GFP::LEU2 MLS1::NAT ICL1::HIS5 +p416-CYC-OLE1-9MYC* | 5B, S5B | This study |
| *mdm30∆ atp12∆ +pMDM30 (MCY1616)* | W303 (MCY970) | *Mata ura3-1 trp1-1 leu2-3,112 his3-11,15 can1-100 RAD5 ADE2 MDM30::KANMX6 ATP12::NAT +PRS316-MDM30* | S4B | This study |
| *mdm30∆ erv29∆ +pMDM30 (MCY1610)* | W303 (MCY970) | *Mata ura3-1 trp1-1 leu2-3,112 his3-11,15 can1-100 RAD5 ADE2 MDM30::KANMX6 ERV29::NAT +PRS316-MDM30* | S4B | This study |
| *mdm30∆ icl1∆ +pMDM30 (MCY1909)* | W303 (MCY970) | *Mata ura3-1 trp1-1 leu2-3,112 his3-11,15 can1-100 RAD5 ADE2 MDM30::KANMX6 ICL1::HIS5 +PRS316-MDM30* | 5C, S5D | This study |
| *ole1∆ + pOLE1-9MYC (MCY1783)* | W303 (MCY553) | *Mata ura3-1 trp1-1 leu2-3,112 his3-11,15 can1-100 RAD5 ADE2 OLE1::KANMX6 + PRS416-ADH-OLE1-9MYC* | S3C | This study |
| *mdm30∆ +pMDM30 (MCY971)* | W303 | *MATα ura3-1 trp1-1 leu2-3,112 his3-11,15 can1-100 MDM30::KANMX6 + PRS316-MDM30* | 4A | [1] |
| *ole1∆ FZO1-13MYC PEX3-6HA +pOLE1-9MYC prom CYC (MCY1797)* | W303 (MCY1781) | *Mata ura3-1 trp1-1 leu2-3,112 his3-11,15 can1-100 RAD5 ADE2 OLE1::KANMX6 + PRS414-CYC-OLE1-9MYC* | S3D | This study |
| *ole1∆ FZO1-13MYC PEX3-6HA +pOLE1-9MYC prom MET25 (MCY1798)* | W303 (MCY1781) | *Mata ura3-1 trp1-1 leu2-3,112 his3-11,15 can1-100 RAD5 ADE2 OLE1::KANMX6 + PRS414-MET25-OLE1-9MYC* | S3D | This study |
| *ole1∆ FZO1-13MYC PEX3-6HA +pOLE1-9MYC prom ADH (MCY1796)* | W303 (MCY1781) | *Mata ura3-1 trp1-1 leu2-3,112 his3-11,15 can1-100 RAD5 ADE2 OLE1::KANMX6 + PRS414-ADH-OLE1-9MYC* | S3D | This study |
| *ole1∆ FZO1-13MYC PEX3-6HA +pOLE1-9MYC prom TEF (MCY1795)* | W303 (MCY1781) | *Mata ura3-1 trp1-1 leu2-3,112 his3-11,15 can1-100 RAD5 ADE2 OLE1::KANMX6 + PRS414-TEF-OLE1-9MYC* | S3D | This study |
| *ole1∆ cit2∆ mt-GFP +pOLE1-9MYC (MCY2023)* | W303 (MCY1835) | *Mata ura3-1 trp1-1 leu2-3,112 his3-11,15 can1-100 RAD5 ADE2 OLE1::KANMX6 CIT2::NAT mt-GFP::LEU2 + PRS416-CYC OLE1-9MYC* | 6A | This study |
| *ole1∆ cat2∆ mt-GFP +pOLE1-9MYC (MCY2032)* | W303 (MCY1835) | *Mata ura3-1 trp1-1 leu2-3,112 his3-11,15 can1-100 RAD5 ADE2 OLE1::KANMX6 CAT2::HIS5 mt-GFP::LEU2 +PRS416-CYC-OLE1-9MYC* | 6A | This study |
| *ole1∆ OM45-GFP RFP-SKL + pOLE1 prom CYC (MCY1936)* | W303 (MCY1899) | *Mata ura3-1 trp1-1 leu2-3,112 his3-11,15 can1-100 RAD5 ADE2 OLE1::KANMX6 OM45-EGFP::HIS5 RFP-SKL::LEU2 +p414-CYC-OLE1* | 7A,7B | This study |
| *ole1∆ OM45-GFP RFP-SKL + pOLE1 prom OLE1 (MCY1935)* | W303 (MCY1899) | *Mata ura3-1 trp1-1 leu2-3,112 his3-11,15 can1-100 RAD5 ADE2 OLE1::KANMX6 OM45-EGFP::HIS5 RFP-SKL::LEU2 +p414-OLE1-OLE1* | 7A,7B | This study |
| *ole1∆ OM45-GFP RFP-SKL + pOLE1 prom TEF (MCY1944)* | W303 (MCY1899) | *Mata ura3-1 trp1-1 leu2-3,112 his3-11,15 can1-100 RAD5 ADE2 OLE1::KANMX6 OM45-EGFP::HIS5 RFP-SKL::LEU2 +p414-TEF-OLE1* | 7A,7B | This study |
| *fzo1∆ pex34∆ mt-GFP +pFZO1 (URA) (MCY1978)* | W303 (MCY1843) | *Mata FZO1::LoxP ura3-1 trp1-1 leu2-3,112 his3-11,15 can1-100 RAD5 ADE2 mt-GFP::LEU2 PEX34::NAT +PRS416-FZO1* | 2F,2E | This study |
| *ole1∆ pex34∆ OM45-GFP RFP-SKL +pOLE1-9MYC (MCY2101)* | W303 (MCY1899) | *Mata ura3-1 trp1-1 leu2-3,112 his3-11,15 can1-100 RAD5 ADE2 OLE1::KANMX6 PEX34::NAT OM45-EGFP::HIS5 RFP-SKL::LEU2 + PRS416-CYC-OLE1-9MYC* | 3F | This study |
| *ole1∆ ubp2∆ OM45-GFP RFP-SKL +pOLE1-9MYC (MCY2091)* | W303 (MCY1899) | *Mata ura3-1 trp1-1 leu2-3,112 his3-11,15 can1-100 RAD5 ADE2 OLE1::KANMX6 UBP2::NAT OM45-EGFP::HIS5 RFP-SKL::LEU2 + PRS416-CYC-OLE1-9MYC* | 3H | This study |
| *ole1∆ ctp1∆ OM45-GFP RFP-SKL +pOLE1-9MYC (MCY2056)* | W303 (MCY1899) | *Mata ura3-1 trp1-1 leu2-3,112 his3-11,15 can1-100 RAD5 ADE2 OLE1::KANMX6 CTP1::NAT OM45-EGFP::HIS5 RFP-SKL::LEU2 + PRS416-CYC-OLE1-9MYC* | 7A,7B | This study |
| *ole1∆ cit2∆ OM45-GFP RFP-SKL +pOLE1-9MYC (MCY2052)* | W303 (MCY1899) | *Mata ura3-1 trp1-1 leu2-3,112 his3-11,15 can1-100 RAD5 ADE2 OLE1::KANMX6 CIT2::NAT OM45-EGFP::HIS5 RFP-SKL::LEU2 + PRS416-CYC-OLE1-9MYC* | 7A,7B, S5E | This study |
| *ole1∆ cat2∆ OM45-GFP RFP-SKL +pOLE1-9MYC (MCY2054)* | W303 (MCY1899) | *Mata ura3-1 trp1-1 leu2-3,112 his3-11,15 can1-100 RAD5 ADE2 OLE1::KANMX6 CAT2::NAT OM45-EGFP::HIS5 RFP-SKL::LEU2 + PRS416-CYC-OLE1-9MYC* | S5D | This study |
| *ole1∆ ctp1∆ mt-GFP +pOLE1-9MYC (MCY2036)* | W303 (MCY1835) | *Mata ura3-1 trp1-1 leu2-3,112 his3-11,15 can1-100 RAD5 ADE2 OLE1::KANMX6 CTP1::NAT mt-GFP::LEU2 + PRS416-CYC OLE1-9MYC* | S5C | This study |
| *ole1∆ preCOX4-mCherry OM45-GFP+ pOLE1-9MYC (MCY2080)* | W303 (MCY1781) | *Mata ura3-1 trp1-1 leu2-3,112 his3-11,15 can1-100 RAD5 ADE2 OLE1::KANMX6 preCOX4-mCherry::HphMX6 OM45-EGFP::HIS5 + PRS416-CYC-OLE1-9MYC* | 6C, 6D | This study |
| *ole1∆ mls1∆ preCOX4-mCherry OM45-GFP+ pOLE1-9MYC (MCY2084)* | W303 (MCY1781) | *Mata ura3-1 trp1-1 leu2-3,112 his3-11,15 can1-100 RAD5 ADE2 OLE1::KANMX6 preCOX4-mCherry::HphMX6 MLS1::NAT OM45-EGFP::HIS5 + PRS416-CYC-OLE1-9MYC* | 6C, 6D, 7C | This study |
| *fzo1∆ PEX3-mCherry mt-BFP mgm1∆ + pGFP-Link-FZO1 (MCY2112)* | W303 (MCY1569) | *Mata FZO1::LoxP ura3-1 trp1-1 leu2-3,112 his3-11,15 can1-100 RAD5 ADE2 PEX3-mCherry::NAT mt-BFP::LEU2 MGM1::KAN +PRS414-GFP-FZO1* | 2C | This study |
| *ole1∆ cit2∆ preCOX4-mCherry OM45-GFP+ pOLE1-9MYC (MCY2088)* | W303 (MCY1781) | *Mata ura3-1 trp1-1 leu2-3,112 his3-11,15 can1-100 RAD5 ADE2 OLE1::KANMX6 preCOX4-mCherry::HphMX6 CIT2::NAT OM45-EGFP::HIS5 + PRS416-CYC-OLE1-9MYC* | 6C | This study |
| *ole1∆ + pRS414-CYC OLE1 + pYeL1-mtGFP (MCY2124)* | W303 (MCY554) | *MATα ura3-1 trp1-1 leu2-3,112 his3-11,15 can1-100 RAD5 ADE2 OLE1::KANMX6 + pRS414-CYC OLE1 + pYeL1-mtGFP* | S6B | This study |
| *ole1∆ + pRS414-TEF OLE1 + pYeL1-mtGFP (MCY2125)* | W303 (MCY554) | *MATα ura3-1 trp1-1 leu2-3,112 his3-11,15 can1-100 RAD5 ADE2 OLE1::KANMX6 + pRS414-TEF OLE1 + pYeL1-mtGFP* | S6B | This study |
| *ole1∆ + pRS414-OLE1 OLE1 + pYeL1-mtGFP (MCY2126)* | W303 (MCY554) | *MATα ura3-1 trp1-1 leu2-3,112 his3-11,15 can1-100 RAD5 ADE2 OLE1::KANMX6 + pRS414-OLE1 OLE1 + pYeL1-mtGFP* | S6B | This study |
| *ole1∆ + pRS414-OLE1 OLE1 + pYeL1-mtRFP (MCY2127)* | W303 (MCY553) | *MATa ura3-1 trp1-1 leu2-3,112 his3-11,15 can1-100 RAD5 ADE2 OLE1::KANMX6 + pRS414-OLE1 OLE1 + pYeL1-mtRFP* | S6B | This study |
| *ole1∆ + pRS414-CYC OLE1 + pYeL1-mtRFP (MCY2128)* | W303 (MCY553) | *MATa ura3-1 trp1-1 leu2-3,112 his3-11,15 can1-100 RAD5 ADE2 OLE1::KANMX6 + pRS414-CYC OLE1 + pYeL1-mtRFP* | S6B | This study |
| *ole1∆ + pRS414-TEF OLE1 + pYeL1-mtRFP (MCY2129)* | W303 (MCY553) | *MATa ura3-1 trp1-1 leu2-3,112 his3-11,15 can1-100 RAD5 ADE2 OLE1::KANMX6 + pRS414-TEF OLE1 + pYeL1-mtRFP* | S6B | This study |
| *mls1∆ ole1∆ + pRS414-CYC OLE1 + pYeL1-mtRFP (MCY2130)* | W303 (MCY553) | *MATa ura3-1 trp1-1 leu2-3,112 his3-11,15 can1-100 RAD5 ADE2 MLS1::NAT OLE1::KANMX6 + pRS414-CYC OLE1 + pYeL1-mtRFP* | S6B | This study |
| *mls1∆ ole1∆ + pRS414-OLE1 OLE1 + pYeL1-mtRFP (MCY2131)* | W303 (MCY553) | *MATa ura3-1 trp1-1 leu2-3,112 his3-11,15 can1-100 RAD5 ADE2 MLS1::NAT OLE1::KANMX6 + pRS414-OLE1 OLE1 + pYeL1-mtRFP* | S6B | This study |
| *mls1∆ ole1∆ + pRS414-TEF OLE1 + pYeL1-mtRFP (MCY2132)* | W303 (MCY553) | *MATa ura3-1 trp1-1 leu2-3,112 his3-11,15 can1-100 RAD5 ADE2 MLS1::NAT OLE1::KANMX6 + pRS414-TEF OLE1 + pYeL1-mtRFP* | S6B | This study |
| *mls1∆ ole1∆ + pRS414-CYC OLE1 + pYeL1-mtGFP (MCY2142)* | W303 (MCY554) | *MATα ura3-1 trp1-1 leu2-3,112 his3-11,15 can1-100 RAD5 ADE2 MLS1::NAT OLE1::KANMX6 + pRS414-CYC OLE1 + pYeL1-mtGFP* | S6B | This study |
| *mls1∆ ole1∆ + pRS414-TEF OLE1 + pYeL1-mtGFP (MCY2143)* | W303 (MCY554) | *MATα ura3-1 trp1-1 leu2-3,112 his3-11,15 can1-100 RAD5 ADE2 MLS1::NAT OLE1::KANMX6 + pRS414-TEF OLE1 + pYeL1-mtGFP* | S6B | This study |
| *mls1∆ ole1∆ + pRS414-OLE1 OLE1 + pYeL1-mtGFP (MCY2144)* | W303 (MCY554) | *MATα ura3-1 trp1-1 leu2-3,112 his3-11,15 can1-100 RAD5 ADE2 MLS1::NAT OLE1::KANMX6 + pRS414-OLE1 OLE1 + pYeL1-mtGFP* | S6B | This study |
| *cit2∆ ole1∆ + pRS414-CYC OLE1 + pYeL1-mtGFP (MCY2145)* | W303 (MCY554) | *MATα ura3-1 trp1-1 leu2-3,112 his3-11,15 can1-100 RAD5 ADE2 CIT2::NAT OLE1::KANMX6 + pRS414-CYC OLE1 + pYeL1-mtGFP* | S6B | This study |
| *cit2∆ ole1∆ + pRS414-TEF OLE1 + pYeL1-mtGFP (MCY2146)* | W303 (MCY554) | *MATα ura3-1 trp1-1 leu2-3,112 his3-11,15 can1-100 RAD5 ADE2 CIT2::NAT OLE1::KANMX6 + pRS414-TEF OLE1 + pYeL1-mtGFP* | S6B | This study |
| *cit2∆ ole1∆ + pRS414-OLE1 OLE1 + pYeL1-mtGFP (MCY2147)* | W303 (MCY554) | *MATα ura3-1 trp1-1 leu2-3,112 his3-11,15 can1-100 RAD5 ADE2 CIT2::NAT OLE1::KANMX6 + pRS414-OLE1 OLE1 + pYeL1-mtGFP* | S6B | This study |
| *cit2∆ ole1∆ + pRS414-CYC OLE1 + pYeL1-mtRFP (MCY2148)* | W303 (MCY553) | *MATa ura3-1 trp1-1 leu2-3,112 his3-11,15 can1-100 RAD5 ADE2 CIT2::NAT OLE1::KANMX6 + pRS414-CYC OLE1 + pYeL1-mtRFP* | S6B | This study |
| *cit2∆ ole1∆ + pRS414-TEF OLE1 + pYeL1-mtRFP (MCY2149)* | W303 (MCY553) | *MATa ura3-1 trp1-1 leu2-3,112 his3-11,15 can1-100 RAD5 ADE2 CIT2::NAT OLE1::KANMX6 + pRS414-TEF OLE1 + pYeL1-mtRFP* | S6B | This study |
| *cit2∆ ole1∆ + pRS414-OLE1 OLE1 + pYeL1-mtRFP (MCY2150)* | W303 (MCY553) | *MATa ura3-1 trp1-1 leu2-3,112 his3-11,15 can1-100 RAD5 ADE2 CIT2::NAT OLE1::KANMX6 + pRS414-OLE1 OLE1 + pYeL1-mtRFP* | S6B | This study |
| W303 *mt-BFP* (MCY1589) | W303 (MCY553) | *Mata ura3-1 trp1-1 leu2-3,112 his3-11,15 can1-100 RAD5 ADE2 mt-BFP::LEU2* | S1A | This study |
| *mt-BFP* FZO1-GFP (MCY1671) | W303 (MCY1589) | *Mata ura3-1 trp1-1 leu2-3,112 his3-11,15 can1-100 RAD5 ADE2 mt-BFP::LEU2 FZO1-EGFP::KANMX6* | S1A | This study |
| *FZO1-13MYC (MCY1119)* | W303 (MCY553) | *Mata ura3-1 trp1-1 leu2-3,112 his3-11,15 can1-100 RAD5 ADE2 FZO1-13Myc-HIS5+* | 1D | This study |
| *mdm30∆ FZO1-13MYC (MCY1131)* | W303 (MCY971) | *MATα ura3-1 trp1-1 leu2-3,112 his3-11,15 can1-100 RAD5 ADE2 mdm30::KANMX FZO1-13Myc-His5+* | 1D | This study |
| *FZO1-GFP (*MCY1415) | W303 (MCY553) | *Mata ura3-1 trp1-1 leu2-3,112 his3-11,15 can1-100 RAD5 ADE2 FZO1::EGFP-HISMX6* | S1B,S1C,S1D,S1E | This study |
| *mdm30∆ FZO1-GFP (*MCY1417) | W303 (MCY553) | *Mata ura3-1 trp1-1 leu2-3,112 his3-11,15 can1-100 RAD5 ADE2 mdm30::KANMX FZO1:EGFP-HIS3MX6* | S1B | This study |

**Reference**

1. Cavellini L, Meurisse J, Findinier J, Erpapazoglou Z, Belgareh-Touze N, Weissman AM, et al. An ubiquitin-dependent balance between mitofusin turnover and fatty acids desaturation regulates mitochondrial fusion. Nat Commun. 2017/06/14 ed. 2017;8: 15832. doi:10.1038/ncomms15832
