# Supplementary material for: Mitofusin-mediated contacts between mitochondria and peroxisomes regulate mitochondrial fusion
Source: PLoS Biol. 2024 Apr 26;22(4):e3002602. doi: 10.1371/journal.pbio.3002602 (PMC11078399; doi:10.1371/journal.pbio.3002602)
Supplement: S2 Table — (DOCX) [file pbio.3002602.s008.docx]

**Supplementary Table 2: Plasmids used in this study.**

| **Name** | **Description** | **Occurrence in the study** | **Reference** |
| --- | --- | --- | --- |
| *pRS314 (MC219)* | CEN, *TRP1*, Amp | 4A,4B,4D, 4E, 6D, S3C, S4A, S4B, S4D | [1] |
| *pRS416-FZO1 (MC322)* | CEN, *FZO1 promoter FZO1*, URA3, Amp | FZO1 shuffling plasmid | [2] |
| *pRS314-FZO1 (MC250)* | CEN, *FZO1 promoter FZO1 TRP1*, Amp | 2E,2F,4A,4B, 4D,4E,5C, S3B, S4A, S4B, S4D | [3] |
| *pRS314-FZO1S201N (MC544)* | CEN, *FZO1 promoter FZO1S201N, TRP1*, Amp | 2E,2F,4B, S3B | [3] |
| *pRS316-MDM30 (MC331)* | CEN, *MDM30 promoter MDM30*, *URA3*, Amp | MDM30 shuffling plasmid | [4] |
| *pRS414-FZO1-13MYC (MC333)* | CEN, *FZO1 promoter FZO1-13MYC*, *TRP1*, Amp | 2A | [4] |
| *pRS414-FZO1-S201N-13MYC (MC389)* | CEN, *FZO1 promoter FZO1S201N*-*13MYC, TRP1*, Amp | 2A | this study |
| *pRS414-ADH GFP-link-Fzo1 (MC538)* | CEN, *ADH promoter GFP-link-FZO1, TRP1*, Amp | 2B | this study |
| *pRS414-ADH GFP-link-FZO1S201N (MC539)* | CEN, *ADH promoter GFP-link-FZO1S201N, TRP1*, Amp | 2B | this study |
| *pRS414-GFP-Link-FZO1 (MC261)* | CEN, *FZO1 promoter GFP-Link-FZO1*, *TRP1*, Amp | 2C, S2D | this study |
| *pRS414-GFP-Link-FZO1S201N (MC426)* | CEN, *FZO1 promoter GFP-Link-FZO1S201N, TRP1*, Amp | 2C, S2D | this study |
| *pRS416-CYC OLE1-9MYC (MC545)* | CEN, *CYC promoter OLE1-9MYC, URA3*, Amp | Ole1 shuffling plasmid | this study |
| *p416-ADH OLE1-9MYC (MC546)* | CEN, *ADH promoter OLE1-9MYC, URA3*, Amp | Ole1 shuffling plasmid | this study |
| *pRS414-MET25 OLE1-9MYC (MC536)* | CEN, *MET25 promoter Ole1-9MYC, TRP1*, Amp | S3D | this study |
| *pRS414-ADH OLE1-9MYC (MC 534)* | CEN, *ADH promoter OLE1-9MYC, TRP1*, Amp | S3D | this study |
| *pRS414-CYC OLE1-9MYC (MC535)* | CEN, *CYC promoter OLE1-9MYC, TRP1*, Amp | S3D | this study |
| *pRS414-TEF OLE1-9MYC (MC533)* | CEN, *TEF promoter OLE1-9MYC, TRP1*, Amp | S3D | this study |
| *pRS414-CYC OLE1 (MC540)* | CEN, *CYC promoter OLE1, TRP1,* Amp | 3A,3B,3C,3D, S2F,3E,3G, S5A,3F,3H,5B,5D,6A,  6B,6C,6D,7A,7B,7C, S3C, S3E, S4A, S5B, S5D, S5E, S5C, S6B | this study |
| *pRS414-TEF OLE1 (MC541)* | CEN, *TEF promoter OLE1, TRP1,* Amp | 3A,3B,3C,3D, S2F,3E,3G, S5A,3F,3H,5B,5D 6A,6B, 6C,6D,  7A,7B,7C, S3C, S3E, S4A, S5B,  S5D, S5E, S5C, S6B | this study |
| *pRS414-OLE1 OLE1 (MC543)* | CEN, *OLE1 promoter OLE1, TRP1,* Amp | 3A,3D, 3E,3G, S5A,3F,3H,5B,  7A,7B,5D,6A,6C,6D,7C, S3F, S3C, S3E, S4A, S3E, S5B, S5D, S5E, S5C, S6B | this study |
| *YIplac128 mito-GFP (MC363)* | *TEF promoter mito-GFP, LEU2/INT*, Amp | 2E,2F,3D, 3E,3G,5D,6A, S2C, S3F, S5B, S5C | - [5] |
| *YIplac128 mRFP-SKL (MC547)* | *FAA2 promoter mRFP-SKL, LEU2/INT*, Amp | Used for construction of *RFP-SKL* tagged strains | this study |
| *YIplac128 mito-BFP (MC460)* | *TEF promoter mito-BFP, LEU2/INT*, Amp | Used for construction of *mt-BFP* tagged strains | this study |
| *pYeL1-mtRFP (MC336)* | *CEN, GAL10 promoter-mtRFP, LEU2, Amp* | S6B | [4] |
| *pYeL1-mtGFP (MC337)* | CEN, GAL10 promoter-mtGFP, LEU2, Amp | S6B | [4] |

**Reference**

1. Sikorski RS, Hieter P. A system of shuttle vectors and yeast host strains designed for efficient manipulation of DNA in Saccharomyces cerevisiae. Genetics. 1989;122: 19–27.

2. Griffin EE, Chan DC. Domain interactions within Fzo1 oligomers are essential for mitochondrial fusion. J Biol Chem. 2006;281: 16599–16606. doi:10.1074/jbc.M601847200

3. Cohen MM, Amiott EA, Day AR, Leboucher GP, Pryce EN, Glickman MH, et al. Sequential requirements for the GTPase domain of the mitofusin Fzo1 and the ubiquitin ligase SCFMdm30 in mitochondrial outer membrane fusion. J Cell Sci. 2011/04/20 ed. 2011;124: 1403–10. doi:10.1242/jcs.079293

4. Cavellini L, Meurisse J, Findinier J, Erpapazoglou Z, Belgareh-Touze N, Weissman AM, et al. An ubiquitin-dependent balance between mitofusin turnover and fatty acids desaturation regulates mitochondrial fusion. Nat Commun. 2017/06/14 ed. 2017;8: 15832. doi:10.1038/ncomms15832

5. Belgareh-Touzé N, Cavellini L, Cohen MM. Ubiquitination of ERMES components by the E3 ligase Rsp5 is involved in mitophagy. Autophagy. 2017;13: 114–132. doi:10.1080/15548627.2016.1252889
